# Supplementary material for: Comparative effectiveness of cervical vs thoracic spinal-thrust manipulation for care of cervicogenic headache: A randomized controlled trial
Source: PLoS One. 2024 Mar 29;19(3):e0300737. doi: 10.1371/journal.pone.0300737 (PMC10980233; doi:10.1371/journal.pone.0300737)
Supplement: S1 File — (DOCX) [file pone.0300737.s003.docx]

**Title:** Pragmatic effects of spinal thrust manipulations on pain parameters: cervical spine versus thoracic spine in Cervicogenic headache – A prospective, single-blinded, randomized controlled study.

Gopal Nambi,^1*^ Mshari Alghadier^1^, Mudathir Mohamedahmed Eltayeb^2^, Osama R. Aldhafian^3^, Ayman K. Saleh^3,4^, Nesreen Alsanousi^5^, Alaa Jameel A. Albarakati^6^, Mohamed A. Omar^4^, Mohamed Nagah Ahmed Ibrahim^4^, Abdehamid A. Attallah^4^, Mohammed Abdelgwad Ismail^4^, Mohamed Elfeshawy^4^

^1^ Department of Health and Rehabilitation Sciences, College of Applied Medical Sciences, Prince Sattam bin Abdulaziz University, Al-Kharj, Saudi Arabia.

^2^ Department of Nursing, College of Applied Medical Sciences, University, Prince Sattam bin Abdulaziz University, Al-Kharj, Saudi Arabia.

^3^ Department of Surgery, College of Medicine, Prince Sattam bin Abdulaziz University, Al-Kharj, Saudi Arabia.

^4^ Department of Orthopedic surgery, Faculty of Medicine for Girls, Al-Azhar University, Cairo, Egypt.

^5^ Department of Biochemistry, College of Medicine, Prince Sattam bin Abdulaziz University, Al-Kharj, Saudi Arabia.

^5^ Department of Surgery, College of Medicine, Umm Al-Qura University, Al-Qunfudah Branch, Makkah, Saudi Arabia.

**Clinical trial registration:** Registered in Clinical trial registry, India **(**www.ctri.nic.in) with registration number: CTRI/2020/06/026092 and registered prospectively.

**Study Protocol**

**Background:** Cervicogenic headache (CgH) is a distinct form of headache and accounts for 17.8% of all headaches and the prevalence rate is between 0.4% and 20%. The prevalence rate of CgH is 0.21% in females and 0.13% in males and has various causative factors. It has a significant negative socioeconomic impact and is a burden on the community and public health. There is ample evidence supporting the use of different manipulative therapy techniques for Cervicogenic Headache (CgH). However, there is a scarcity in the best available technique in the treatment of CgH patients.

**Objective:** The objective of the study is to find and compare the pragmatic effects of cervical spine over thoracic spine manipulation and conventional physiotherapy in CgH patients.

**Design:** The trial was a parallel-group, prospective, single-blinded, randomized controlled trial. The required participants were screened by a physician at the University hospital between 1^st^ July 2020 and 31^st^ July 2022 in accordance with the Cervicogenic headache diagnostic criteria 11.2.1 from the ICHD-3 (International Classification of Headache Disorders). Ninety-six (N=96) participants who fulfilled the eligibility criteria were randomly allocated into three groups equally: the cervical spine manipulation (CSM; n=32), thoracic spine manipulation (TSM; n=32), and conventional physiotherapy (CPT; n=32) groups through a computer-generated simple random table and the allocation of the participants to each group was concealed using sealed envelopes. The computer did not generate the group until it was time to randomize each participant, ensuring that the allocation was concealed. No significant changes were made while the study was being carried out because it was designed as a follow-up to a pilot study and the 6-month follow-up data collection was completed on 31^st^ January 2023.

The research was conducted at #####, and the Department Ethical Committee (DEC) granted ethical approval under the reference number RHPT/019/042. The DEC accepted the study protocol as well as the informed consent forms. The study involved human participants which followed the instructions outlined in the Declaration of Helsinki (1964) and prospectively registered in clinical trial.gov.in CTRI/2020/06/026092 on June 24, 2020.

***Subjects***

Patients aged between 18-60 years, suffering from CgH (>3 months) were screened to include in the study. They were diagnosed based on the diagnostic criteria developed by the Cervicogenic Headache International Study Group (CHISG) by an orthopedic surgeon with twenty years of clinical experience in diagnosing and treating the CgH and it falls under the International classification of disease -10 (ICD-10) code of G44. 841. Patients with pain intensity ≥3 on a numerical pain rating scale (NPRS), CgH resulting from pain in the neck followed by headache, limited neck movements and neck stiffness and cervical spine disorders were allowed to participate in the study. Other primary headaches such as migraine and tension-type headaches (TTH), whiplash injuries, participants who show signs of the five ‘D’s’ (dizziness, drop attacks, dysarthria, dysphagia, diplopia) or who have signs of the three ‘N’s (nystagmus, nausea, other neurological symptoms (cord compression or nerve root involvement), contraindications to manipulative therapy (tumour, degenerative and inflammatory arthritis, osteoporosis, dislocation, fractures, and steroid intake), underwent previous head and neck surgeries, had physiotherapy or other complementary therapies in the last three months were excluded.

The list of participants was compiled from the #####, and requests were sent to the participants via personal e-mail. Two orthopaedic surgeons with ten to fifteen years of experience diagnosed the CgH patients after they consented to participate in the study. The research was carried out at the Department of Physical Therapy and Health Rehabilitation, #####, using the recommended study protocols.

***Intervention***

Certified physiotherapists having 10 – 15 years of experience in spinal manipulation for CgH provided the approved technique in each group. All the participants in the three groups had given their willingness to participate in the study after getting detailed information about the study protocol. The treatment consisted of a 10-minute hydro collator pack application to relax the muscles of the area around the neck and upper back. Following this, the participant’s neck muscles and joints were assessed for any musculoskeletal dysfunction. After that, the participants were given the manipulation techniques as per the directions of the study protocol. Standardized treatment techniques were used for all the group participants to reduce intervention bias. The procedures of intervention and follow-up measurements were recorded in standardized forms. During the study period, the participants were asked to refrain from taking any other type of intervention, they received the concerned interventions 3 times per week for 4 weeks.

2.3.1 Spinal manipulation therapy

Peterson and Bergman defined SMT as a high-velocity low-amplitude thrust (HVLAT) technique. Four experienced physiotherapists having experience in SMT performed this technique after evaluation of each participant by physical examination and palpation. The therapist located the sites of abnormal changes in each vertebra and then manipulated the area following the study's recommendations. If any participant reported any new red flag signs or showed no signs for manipulation, such as no pain or musculoskeletal dysfunction, then the procedure was not performed.

*2.3.1.1 Cervical spine manipulation (CSM)*

To perform the C1-C2 cervical spine manipulation (CSM) the participant was instructed to lie down in a face-up position with upper and lower extremities kept aside relaxed. The head was kept in a neutral position and the treating therapist stands at the patient's head side and holds the chin of the patient with the right side hand. The therapist left hand holds the posterior aspect of the head and does two to three free rotatory movements. Afterwards, the therapist did HVLAT technique in either the right or left direction based upon the symptoms informed by the patient. The manipulation was done first on the pain-free side and then on the painful side and the rotation range is limited by the target vertebra.

*2.3.1.2 Thoracic spine manipulation (TSM)*

To perform the T1-T2 thoracic spine manipulation (TSM) the patient lays down in a face-up position with his arms crossed across his chest in a vertical direction, with elbows in the top position. The therapist stands beside the participant and faces towards the him. The treating therapist's left hand held the patient's both elbows together to apply downward force, while the right hand was kept under the spinous process of the target vertebra's lower vertebra with the thenar eminence and middle phalanx of the middle finger. The vertical downward thrust (anterior to posterior) was applied to the target vertebra by the therapist's left hand, and the thrust was adjusted by the right hand through pronation and radial deviation. Manipulation was performed by lowering the knees to generate thrust force while keeping the therapist's spine straight and head up.

2.3.2 Conventional physiotherapy (CPT)

The participants of the CPT group received massage therapy for 15 minutes using Queen Helene, Cocoa Butter face & body cream, New York, USA. The patient lays down in a face-up position, with the posterior aspect of the head resting on a folded towel. The treating therapist stands by the patient's head side and uses the tips of the middle fingers of both hands to perform circular kneading on both sides of the C1 to C7 vertebra. This maneuver was repeated 3 times for each cervical vertebra, beginning from the C7 vertebra and working towards the C1 vertebra. The neck was then turned to the other side, and the same manoeuvre was repeated from insertion to origin on the sub-occipital and paravertebral muscles.

Participants of all three groups were asked to perform neck isometric exercises three times per day, every day. The patient was asked to keep his hand over his forehead and resist the forward movement of his neck for 10 seconds and the same movement was repeated 15 times. Similarly, the patient was asked to keep the hand on the posterior and lateral sides of the head, and resist the backward and sideways movements of the neck. Also, static active stretching exercises for the upper trapezius, levator scapulae, scalene, and sternocleidomastoid muscles were taught to the patients, which was maintained for the 30s with 3 repetitions. The patients were instructed to keep doing this set of exercises after 4 weeks of various intervention protocols and they were asked to maintain an exercise log book to check the treatment compliance.

***Outcome measures***

All the outcome measures were recorded by a blinded physiotherapist, and the scores were entered in a data sheet. The scores were measured at the beginning of the study, after 4-weeks, 8-weeks, and at 6-months.

2.4.1 Primary outcome

*CgH frequency:* It is a self-administered outcome variable where the patient enters his CgH pain experience in a medical log book every evening to find the number of painful days in 4-weeks.

2.4.2 Secondary outcome

*CgH pain intensity:* The pain intensity of CgH was assessed using an 11-point numerical pain rating scale (NPRS). Patients rated their typical level of pain status during the previous week on a 10 cm horizontal line, with one end 0 representing "no pain" and the other end 10 representing "worst pain imaginable."

*CgH disability:* The Headache Impact Test (HIT) questionnaire is a valid and reliable instrument to assess the level of disability in CgH patients. It consists of six items: pain, social functioning, role functioning, vitality, cognitive functioning, and psychological distress. The score categories are no or mild disability (49 or less), moderate disability (50–55), severe disability (56–59), and complete disability (60–78).

*NP frequency:* It is a self-administered outcome variable where the patient enters his neck pain experience in a medical log book every evening to find the number of painful days in 4-weeks.

*NP intensity:* The pain intensity of neck pain was assessed using an 11-point numerical pain rating scale (NPRS). Patients rated their average pain intensity over the past week on a 10 cm horizontal line, with one end 0 representing "no pain" and the other end 10 representing "worst pain imaginable."

*NP pressure threshold:* It is the lowest intensity at which a given stimulus is perceived as painful and it was measured using an instrument Algometer (Baseline, 22-pound dolorimeter, ID, USA). The tip was placed over the fixed points on the back of the neck and assessed through palpation. It is a reliable and valid tool for determining pain threshold.

*Cervical flexion–rotation test (FRT):* The cervical flexion–rotation test is done with the patient in a supine lying position. The therapist passively maintains the patient's neck into full flexion to relax the structures of the middle and lower cervical spine, then the patient's head is passively rotated in each direction while the flexed position is maintained and the range of motion is measured.

*Neck disability index (NDI):* It is a reliable and valid self-reported questionnaire with ten items scored on a 0 to 5 scale. The grades of disability are determined based on the scores obtained, which are as follows: 10-29% mild; 30-49% moderate; 50-69% severe; 70% or more is a complete disability.

*Quality of life:* The EQ-5D (Euro Qol 5D) is self-administered health related quality of life (HRQOL) questionnaire, which measures the five dimensions of quality of life. It includes mobility, self-care, usual activities, pain/discomfort, and anxiety/depression. It is used to assess the CgH patients' overall quality of life with a scale of 0 (worst) to 100 (best).

***Sample size***

For calculating the number of subjects to be include in the study, the primary outcome measure CgH frequency in days was taken into consideration based on previous pilot study which found the effect of spinal manipulation in the treatment of CgH, with 10 subjects in each group. Using the G-Power software (version 3.1.9.2; Franz Faul, University of Kiel, Germany), assuming a two-sided α = 0.05, and power (1-β = 0.80), to detect an effect size of 1.2 CgH days and mean difference of 4 CgH days (between groups) and a standard deviation of 0.5, an approximately 28 samples were required. In assuming 10% dropout, we enrolled 32 subjects in each group.

**Statistical analysis plan**

The normality of study participants’ demographic characteristics was analyzed through the Kolmogorov-Smirnov test. The outcome data were presented in the form of a mean and standard deviation with a 95% confidence interval. The effects of treatment at different time intervals were analyzed using a 3 × 4 linear mixed model analysis (LMM), with treatment groups (cervical spine manipulation, thoracic spine manipulation, and conventional physical therapy) and time intervals (baseline, four weeks, eight weeks, and at six months) and a statistical significance level of α=0.05. All the statistical tests were done using GraphPad-Prism (version 9.1), Boston, MA, USA.

**Informed Consent Form**

Name of patient: Age/Gender:

**Study Title:** “Pragmatic effects of spinal thrust manipulations on pain parameters: cervical spine versus thoracic spine in Cervicogenic headache – A prospective, single-blinded, randomized controlled study.”

**Introduction and Purpose of Study:** You have been selected to take part in a study “Pragmatic effects of spinal thrust manipulations on pain parameters: cervical spine versus thoracic spine in Cervicogenic headache – A prospective, single-blinded, randomized controlled study.”

**Study Information**

**Outline of Procedures:** At first consultation you will be screened and evaluated for suitability of the study. You are requested to attend 4 sessions a week for 4 weeks’ period. If you are taking any medication or undergoing any other form of treatment for your pain, you may be excluded from the study.

**Risks and Discomfort:** The treatment is safe and is unlikely to cause any adverse side effects. All treatments will be performed by qualified physiotherapist.

**Benefits of the study:** This study will assist the physiotherapy profession in expanding its knowledge of this condition and thus making future treatment of patients suffering from chronic low back pain more effective.

**Withdrawal from the Study:** You are free to withdraw at any stage with no negative repercussions to your health care.

**Remuneration:** Patients taking part in the study will not be offered any other form of remuneration for taking part in the study.

**Costs of Study:** Treatment for the duration of the research process will be free of charge. **Confidentiality:** All patient information and results will be kept confidential and can be shared for research purpose if required.

I have been explained about this research in which I agreed to participate. I know that I am giving this consent without any force. I can discontinue the study any time without any reason and that will not affect my treatment that I have been informed. My identity can be disclosed for any other follow up research.

**Signature of Patient Signature of Researcher**

I certify that I have explained to the participant about nature, purpose, potential benefits & possible risk of the indicated procedure. The information collected will be kept confidential.

**Signature of Witness Signature of Researcher**

**DECLARATIONS**

**Ethics approval and consent to participate:** The study obtained the study ethical approval from Department Ethical Committee (DEC), Prince Sattam bin Abdulaziz University, Al-Kharj, Saudi Arabia with the reference number of RHPT/019/042. Written consent was obtained from all the participants who involved in the study.

**Clinical trial registration:** CTRI/2020/06/026092 trial registered prospectively on 24/06/2020.

**Consent for publication:** No individual data is contained in this publication.

**Availability of data and materials:** Data is not publically available, but can be obtained from the corresponding author on request.

**Competing interest:** None declared

**Funding:** Self-funded study.

Acknowledgement: This study is supported via funding from Prince Sattam bin Abdulaziz University project number (PSAU/2023/R/1444).
