# Supplementary material for: Comparative effectiveness of cervical vs thoracic spinal-thrust manipulation for care of cervicogenic headache: A randomized controlled trial
Source: PLoS One. 2024 Mar 29;19(3):e0300737. doi: 10.1371/journal.pone.0300737 (PMC10980233; doi:10.1371/journal.pone.0300737)
Supplement: Attachment — Submitted filename: PONE-D-23-12627.pdf [file pone.0300737.s004.pdf]

PLOS ONE

Pragmatic effects of spinal thrust manipulations on pain parameters: cervical spine versus thoracic spine in Cervicogenic headache – A prospective, single-blinded, randomized controlled study.

--Manuscript Draft--

Manuscript Number:	PONE-D-23-12627
Article Type:	Clinical Trial
Full Title:	Pragmatic effects of spinal thrust manipulations on pain parameters: cervical spine versus thoracic spine in Cervicogenic headache – A prospective, single-blinded, randomized controlled study.
Short Title:	Manipulation techniques in Cervicogenic Headache.
Corresponding Author:	Gopal Nambi, PhD<br>Prince Sattam bin Abdulaziz University College of Applied Medical Sciences<br>Al Kharj, SAUDI ARABIA
Keywords:	Cervicogenic headache; cervical spine; thoracic spine; manipulation; physiotherapy
Abstract:	Background<br><br>There is ample evidence supporting the use of different manipulative therapy techniques for Cervicogenic Headache (CgH). However, there is a scarcity in the best available technique in the treatment of CgH patients. Therefore, the objective of the study is to find and compare the pragmatic effects of cervical spine over thoracic spine manipulation and conventional physiotherapy in CgH patients.<br><br>Design, Setting, Participants<br><br>It is a prospective, single-blinded, randomized controlled study conducted between July 2020 and January 2023 at the University hospital. N=96 eligible CgH participants were selected based on selection criteria and they were divided into cervical spine manipulation (CSM; n=32), thoracic spine manipulation (TSM; n=32) and conventional physiotherapy (CPT; n=32) groups and they were received respective treatment for four weeks. Primary (CgH frequency) and secondary (CgH pain intensity, CgH disability, neck pain frequency, neck pain intensity, neck pain threshold, flexion rotation test (right and left), neck disability index and quality of life) scores were measured. The effects of treatment at various intervals were analyzed with a 3 × 4 linear mixed model analysis (LMM), with treatment group (cervical spine manipulation, thoracic spine manipulation, and conventional physiotherapy) and time intervals (baseline, 4 weeks, 8 weeks, and 6 months) and the statistical significance level was set at P < 0.05.<br><br>Results<br><br>The baseline demographic and clinical variables did not show any significance difference (p>0.05). The reports of the CSM, TSM and CPT group were compared between the groups. Four weeks following training CSM group showed more significant changes in primary (CgH frequency) and secondary (CgH pain intensity, CgH disability, neck pain frequency, pain intensity, pain threshold, flexion rotation test (Right and left), neck disability index and quality of life) than TSM and CPT groups (p<0.001). The same gradual improvement can be seen in CSM group than TSM and CPT groups (p<0.001) in the above variables at 8 weeks and 6 months follow up.<br><br>Conclusion<br><br>The reports of the current randomized clinical study found that cervical spine manipulation resulted in significantly better improvements in pain parameters (intensity, frequency and threshold) functional disability and quality of life in CgH than thoracic spine manipulation and conventional physiotherapy.
Order of Authors:	Gopal Nambi, PhD

Powered by Editorial Manager® and ProduXion Manager® from Aries Systems Corporation

	Mshari Alghadier, PhD
	Mudathir Mohamedahmed Eltayeb, PhD
	Osama R. Aldhafian, MD
	Ayman K. Saleh, MD
	Nesreen Alsanousi, PhD
	Alaa Jameel A. Albarakati, MD
	Mohamed A. Omar, MD
	Mohamed Nagah Ahmed Ibrahim, MD
	Abdehamid A. Attallah, MD
	Mohammed Abdelgwad Ismail, MD
	Mohamed Elfeshawy, PhD
**Additional Information:**	
**Question**	**Response**
**Financial Disclosure**<br><br>Enter a financial disclosure statement that describes the sources of funding for the work included in this submission. Review the submission guidelines for detailed requirements. View published research articles from *PLOS ONE* for specific examples.<br><br>This statement is required for submission and **will appear in the published article** if the submission is accepted. Please make sure it is accurate.<br><br>**Unfunded studies**<br>Enter: *The author(s) received no specific funding for this work.*<br><br>**Funded studies**<br>Enter a statement with the following details:<br>• Initials of the authors who received each award<br>• Grant numbers awarded to each author<br>• The full name of each funder<br>• URL of each funder website<br>• Did the sponsors or funders play any role in the study design, data collection and analysis, decision to publish, or preparation of the manuscript?<br>• **NO** - Include this sentence at the end of your statement: *The funders had no role in study design, data collection and analysis, decision to publish, or preparation of the manuscript.*<br>• **YES** - Specify the role(s) played.	This study is supported via funding from Prince Sattam bin Abdulaziz University project number (PSAU/2023/R/1444).

* typeset	
**Competing Interests**<br><br>Use the instructions below to enter a competing interest statement for this submission. On behalf of all authors, disclose any competing interests that could be perceived to bias this work—acknowledging all financial support and any other relevant financial or non-financial competing interests.<br><br>This statement is required for submission and **will appear in the published article** if the submission is accepted. Please make sure it is accurate and that any funding sources listed in your Funding Information later in the submission form are also declared in your Financial Disclosure statement.<br><br>View published research articles from *PLOS ONE* for specific examples.<br><br>**NO authors have competing interests**<br><br>Enter: *The authors have declared that no competing interests exist.*<br><br>**Authors with competing interests**<br><br>Enter competing interest details beginning with this statement:<br><br>*I have read the journal's policy and the authors of this manuscript have the following competing interests: [insert competing interests here]*<br><br>* typeset	The authors have declared that no competing interests exist.
**Ethics Statement**<br><br>Enter an ethics statement for this submission. This statement is required if the study involved:	This study was designed and conducted in accordance with the principles of the Declaration of Helsinki and it has been approved by the Department Ethics Committee, Prince Sattam bin Abdulaziz University, Saudi Arabia with an ethical approval number RHPT/019/042. The participants were informed about the harms and benefits of the research through an information form. Subjects who consented to participate in the study were selected for the study and the written informed consent was obtained from

Powered by Editorial Manager® and ProduXion Manager® from Aries Systems Corporation

• Human participants<br>• Human specimens or tissue<br>• Vertebrate animals or cephalopods<br>• Vertebrate embryos or tissues<br>• Field research<br><br>Write "N/A" if the submission does not require an ethics statement.<br><br>General guidance is provided below. Consult the submission guidelines for detailed instructions. **Make sure that all information entered here is included in the Methods section of the manuscript.**	all subjects.

Powered by Editorial Manager® and ProduXion Manager® from Aries Systems Corporation

**Format for specific study types**<br><br>**Human Subject Research (involving human participants and/or tissue)**<br>• Give the name of the institutional review board or ethics committee that approved the study<br>• Include the approval number and/or a statement indicating approval of this research<br>• Indicate the form of consent obtained (written/oral) or the reason that consent was not obtained (e.g. the data were analyzed anonymously)<br><br>**Animal Research (involving vertebrate animals, embryos or tissues)**<br>• Provide the name of the Institutional Animal Care and Use Committee (IACUC) or other relevant ethics board that reviewed the study protocol, and indicate whether they approved this research or granted a formal waiver of ethical approval<br>• Include an approval number if one was obtained<br>• If the study involved *non-human primates*, add *additional details* about animal welfare and steps taken to ameliorate suffering<br>• If anesthesia, euthanasia, or any kind of animal sacrifice is part of the study, include briefly which substances and/or methods were applied<br><br>**Field Research**<br><br>Include the following details if this study involves the collection of plant, animal, or other materials from a natural setting:<br>• Field permit number<br>• Name of the institution or relevant body that granted permission	
**Data Availability**<br><br>Authors are required to make all data underlying the findings described fully available, without restriction, and from the time of publication. PLOS allows rare exceptions to address legal and ethical concerns. See the PLOS Data Policy and FAQ for detailed information.	Yes - all data are fully available without restriction

Powered by Editorial Manager® and ProduXion Manager® from Aries Systems Corporation

A Data Availability Statement describing where the data can be found is required at submission. Your answers to this question constitute the Data Availability Statement and **will be published in the article**, if accepted.<br><br>**Important:** Stating 'data available on request from the author' is not sufficient. If your data are only available upon request, select 'No' for the first question and explain your exceptional situation in the text box.<br><br>Do the authors confirm that all data underlying the findings described in their manuscript are fully available without restriction?	
**Describe where the data may be found in full sentences. If you are copying our sample text, replace any instances of XXX with the appropriate details.**<br><br>• If the data are **held or will be held in a public repository**, include URLs, accession numbers or DOIs. If this information will only be available after acceptance, indicate this by ticking the box below. For example: *All XXX files are available from the XXX database (accession number(s) XXX, XXX.).*<br>• If the data are all contained **within the manuscript and/or Supporting Information files**, enter the following: *All relevant data are within the manuscript and its Supporting Information files.*<br>• If neither of these applies but you are able to provide **details of access elsewhere**, with or without limitations, please do so. For example:<br><br>*Data cannot be shared publicly because of [XXX]. Data are available from the XXX Institutional Data Access / Ethics Committee (contact via XXX) for researchers who meet the criteria for access to confidential data.*<br><br>*The data underlying the results presented in the study are available from (include the name of the third party*	The data underlying the results presented in the study are available by contacting the corresponding author.

and contact information or URL).<br>• This text is appropriate if the data are owned by a third party and authors do not have permission to share the data.<br><br>* typeset	
Additional data availability information:	Tick here if your circumstances are not covered by the questions above and you need the journal’s help to make your data available.

Manuscript

Click here to access/download;Manuscript;Final manuscript.docx

Title: Pragmatic effects of spinal thrust manipulations on pain parameters: cervical spine versus thoracic spine in Cervicogenic headache – A prospective, single-blinded, randomized controlled study.

Running Title: Manipulation techniques in Cervicogenic Headache.

Gopal Nambi,[1*] Mshari Alghadier[1], Mudathir Mohamedahmed Eltayeb[2], Osama R. Aldhafian[3], Ayman K. Saleh[3,4], Nesreen Alsanousi[5], Alaa Jameel A. Albarakati[6], Mohamed A. Omar[4], Mohamed Nagah Ahmed Ibrahim[4], Abdehamid A. Attallah[4], Mohammed Abdelgwad Ismail[4], Mohamed Elfeshawy[4]

[1] Department of Health and Rehabilitation Sciences, College of Applied Medical Sciences, Prince Sattam bin Abdulaziz University, Al-Kharj, Saudi Arabia.

[2] Department of Nursing, College of Applied Medical Sciences, University, Prince Sattam bin Abdulaziz University, Al-Kharj, Saudi Arabia.

[3] Department of Surgery, College of Medicine, Prince Sattam bin Abdulaziz University, Al-Kharj, Saudi Arabia.

[4] Department of Orthopedic surgery, Faculty of Medicine for Girls, Al-Azhar University, Cairo, Egypt.

[5] Department of Biochemistry, College of Medicine, Prince Sattam bin Abdulaziz University, Al-Kharj, Saudi Arabia.

[5] Department of Surgery, College of Medicine, Umm Al-Qura University, Al-Qunfudah Branch, Makkah, Saudi Arabia.

*Corresponding Author:

Gopal Nambi, Associate Professor, Department of Physical Therapy and Health Rehabilitation, College of Applied Medical Sciences, Prince Sattam bin Abdulaziz University, Al-Kharj, Saudi Arabia.

E-mail: physio_gopal@rediffmail.com, Contact No: 00966 501878382

DECLARATIONS

Ethics approval and consent to participate: This study was designed and conducted in accordance with the principles of the Declaration of Helsinki and it has been approved by the Department Ethics Committee, Prince Sattam bin Abdulaziz University, Saudi Arabia with an ethical approval number RHPT/019/042. The participants were informed about the harms and

benefits of the research through an information form. Subjects who consented to participate in the study were selected for the study and the written **informed consent was obtained from all subjects.**

Clinical trial registration: Registered in Clinical trial registry, India (www.ctri.nic.in) with registration number: CTRI/2020/06/026092 and registered prospectively.

Consent for publication: No individual data is contained in this publication.

Availability of data and materials: Data is available.

Competing interest: None declared

Funding: This study is supported via funding from Prince Sattam bin Abdulaziz University project number (PSAU/2023/R/1444).

Author Contributions: Gopal Nambi (GN), Mshari Alghadier (MA), Mudathir Mohamedahmed Eltayeb (MM), Osama R. Aldhafian (OA), Ayman K. Saleh (AS), Nesreen Alsanousi (NA), Alaa Jameel A. Albarakati (AA), Mohamed A. Omar (MO), Mohamed Nagah Ahmed Ibrahim (MI), Abdehamid A. Attalah (AA), Mohammed Abdelgwad Ismail (MI) Mohamed Elfeshawy (ME).

Conceptualization: GN, MA, MM, OA, AS, NA, AA, MO, MI, AA, MI, ME. Data curation: MM, OA, AS, NA, AA, MO, MI, ME. Formal analysis: GN, MA, AS, NA, AA, MO, MI, MI, ME. Funding acquisition: GN, MA, MM, OA, MO, MI, AA, MI, ME. Investigation: GN, MA, MM, OA, AS, NA, AA, MO. Methodology: GN, MA, MM, OA, MO, MI, AA, MI. Project administration: MA, MM, AS, NA, AA, AA, MI, ME. Resources: MM, OA, AS, NA, AA, AA, MI, ME. Software: OA, AS, NA, AA, AA, MI, ME. Validation: MM, OA, AS, MO, MI, AA, MI, ME. Visualization: GN, MM, OA, AS, MO, MI, MI, ME. Writing—original draft: MM, OA, AS, NA, MI, AA, MI. Writing—review and editing: GN, MA, MM, OA, AS, NA, AA, MO, MI, AA, MI, ME.

Acknowledgments: This study is supported via funding from Prince Sattam bin Abdulaziz University project number (PSAU/2023/R/1444).

Abstract:

Background: There is ample evidence supporting the use of different manipulative therapy techniques for Cervicogenic Headache (CgH). However, there is a scarcity in the best available technique in the treatment of CgH patients. Therefore, the objective of the study is to find and compare the pragmatic effects of cervical spine over thoracic spine manipulation and conventional physiotherapy in CgH patients.

Design, Setting, Participants: It is a prospective, single-blinded, randomized controlled study conducted between July 2020 and January 2023 at the University hospital. N=96 eligible CgH participants were selected based on selection criteria and they were divided into cervical spine manipulation (CSM; n=32), thoracic spine manipulation (TSM; n=32) and conventional physiotherapy (CPT; n=32) groups and they were received respective treatment for four weeks. Primary (CgH frequency) and secondary (CgH pain intensity, CgH disability, neck pain frequency, neck pain intensity, neck pain threshold, flexion rotation test (right and left), neck disability index and quality of life) scores were measured. The effects of treatment at various intervals were analyzed with a 3 × 4 linear mixed model analysis (LMM), with treatment group (cervical spine manipulation, thoracic spine manipulation, and conventional physiotherapy) and time intervals (baseline, 4 weeks, 8 weeks, and 6 months) and the statistical significance level was set at $P < 0.05$.

Results: The baseline demographic and clinical variables did not show any significance difference ($p>0.05$). The reports of the CSM, TSM and CPT group were compared between the groups. Four weeks following training CSM group showed more significant changes in primary (CgH frequency) and secondary (CgH pain intensity, CgH disability, neck pain frequency, pain intensity, pain threshold, flexion rotation test (Right and left), neck disability index and quality of life) than TSM and CPT groups ($p=0.001$). The same gradual improvement can be seen in CSM group than TSM and CPT groups ($p=0.001$) in the above variables at 8 weeks and 6 months follow up.

Conclusion: The reports of the current randomized clinical study found that cervical spine manipulation resulted in significantly better improvements in pain parameters (intensity, frequency and threshold) functional disability and quality of life in CgH than thoracic spine manipulation and conventional physiotherapy.

Keywords: Cervicogenic headache; cervical spine; thoracic spine; manipulation; physiotherapy

Clinical trial registration: CTRI/2020/06/026092 trial registered prospectively on 24/06/2020.

1. INTRODUCTION

Globally, headache disorders affect approximately 66% of the population between the ages of 18 and 65 years at least once a year. 66% of men and 57% of women report headache once in their lifetime which reduces the quality of life, work productivity and costs to society.[1] Cervicogenic headache (CgH) is a distinct form of headache and accounts for 17.8% of all headaches and the prevalence rate is between 0.4% and 20%. The prevalence rate of CgH is 0.21% in females and 0.13% in males and has various causative factors.[2] It has a significant negative socioeconomic impact and is a burden on the community and public health. The cause of the headache is located in the neck region and the pain is made worse by movements of the head and neck.[3] The most accepted mechanism of CgH is found between the trigeminal nerve and C1 – C3 nerves in the trigemino-cervical nucleus.[4] It usually arises from musculoskeletal structures such as the cervical vertebra, intervertebral disc, or paravertebral muscles. The clinical features of CgH include unilateral headache, limited range of motion (ROM) at the neck, and radiating pain to the head or face region.[5]

Generally, CgH is diagnosed on the bases of a detailed history and clinical assessment.[6] Physical examinations typically reveal pain in the cervical region – neck pain (NP), decreased neck movements, upper quarter muscle tightness and loss of muscle characteristics.[7] The flexion rotation test (FRT) is a valid, reliable and accurate method for assessing cervical ROM, it is recognized as the diagnostic tool for CgH.[8] The management of CgH consists of pharmacological and non-pharmacological methods, in which the pharmacological means are associated with many side effects.[9] There are many non-pharmacological treatment modalities available such as; physical modalities, positional therapy, muscle strengthening exercises, ergonomic guidance and patient education etc.[10] It has been estimated that 34% of US citizens receive some sort of physiotherapy for CgH each year.[11]

In physiotherapy, joint mobilization and manipulation are the most commonly used treatment modality for treating CgH patients.[12] The two types of manipulation techniques used in CgH are cervical spine manipulation (CSM) and thoracic spine manipulation (TSM) and both of these techniques use high velocity, low amplitude thrusts (HVLAT). Some studies looked solely at the effects of manipulating the cervical spine in cases of Cervicogenic

headache.[5,13,14] McDevitt AW et al. found that thoracic spine manipulation alone significantly improved neck-related disability in CgH, but had no effect on headache-related disability but participants reported overall improvement of their condition.[15] Dunning JR et al. looked into the mixed effects of cervical and thoracic spine manipulation in CgH patients at the same time and found clinically better effects in pain parameters and functional disability at 3 months follow-up.[16] However, studies have shown that treating Cervicogenic headache with combined spinal manipulation (cervical and thoracic) or just the cervical spine or thoracic spine alone is effective. However, so far no studies have compared and investigated the individual effects of cervical spine manipulation, thoracic spine manipulation ~~and~~ conventional physiotherapy for treating CgH patients.

Numerous studies have supported the application of various manipulative therapy approaches for the treatment of CgH.[13-16] However there are no specific treatment protocol in relation to the type of manipulation technique that is most efficient. Nevertheless, there is a scarcity of evidence in comparing the individual effects of cervical ~~and~~ thoracic manipulation approaches in Cervicogenic headache, particularly regarding its clinical and functional aspects. Additionally, no studies have attempted to address the shortcomings and gaps observed in the existing literature on the management of CgH, such as a lack of comparison between manipulative therapy techniques, poor trial designs and quality and small sample sizes. Therefore, our study objective was to compare and investigate the pragmatic effects of cervical and thoracic manipulation techniques on patients with Cervicogenic headache. This randomized clinical trial hypothesized that there ~~is a~~ difference in primary and secondary outcome measures between cervical spine manipulation, thoracic spine manipulation, and conventional physiotherapy for the treatment of CgH patients.

2. MATERIALS AND METHODS

2.1 Study design

The trial was a parallel-group, prospective, single-blinded, randomized controlled trial. The required participants were screened by a physician at the University hospital between 1st July 2020 and 31st July 2022 in accordance with the Cervicogenic headache diagnostic criteria 11.2.1 from the ICHD-3 (International Classification of Headache Disorders).[17] Ninety-six (N=96) participants who fulfilled the eligibility criteria were randomly allocated into three groups equally: the cervical spine manipulation (CSM; n=32), thoracic spine manipulation

(TSM; n=32), and conventional physiotherapy (CPT; n=32) groups through a computer-generated simple random table and the allocation of the participants to each group was concealed using sealed envelopes. The computer did not generate the group until it was time to randomize each participant, ensuring that the allocation was concealed. No significant changes were made while the study was being carried out because it was designed as a follow-up to a pilot study and the 6-month follow-up data collection was completed on 31st January 2023.

The research was conducted at #####, and the Department Ethical Committee (DEC) granted ethical approval under the reference number RHPT/019/042. The DEC accepted the study protocol as well as the informed consent forms. The study involved human participants which followed the instructions outlined in the Declaration of Helsinki (1964) and prospectively registered in clinical trial.gov.in CTRI/2020/06/026092 on June 24, 2020.

2.2 Participants

Patients aged between 18-60 years, suffering from CgH (>3 months) were screened to include in the study. They were diagnosed based on the diagnostic criteria developed by the Cervicogenic Headache International Study Group (CHISG) by an orthopedic surgeon with twenty years of clinical experience in diagnosing and treating the CgH and it falls under the International classification of disease -10 (ICD-10) code of G44. 841.[6] Patients with pain intensity ≥3 on a numerical pain rating scale (NPRS), CgH resulting from pain in the neck followed by headache, limited neck movements and neck stiffness and cervical spine disorders were allowed to participate in the study. Other primary headaches such as migraine and tension-type headaches (TTH), whiplash injuries, participants who show signs of the five 'D's' (dizziness, drop attacks, dysarthria, dysphagia, diplopia) or who have signs of the three 'N's (nystagmus, nausea, other neurological symptoms (cord compression or nerve root involvement), contraindications to manipulative therapy (tumour, degenerative and inflammatory arthritis, osteoporosis, dislocation, fractures, and steroid intake), underwent previous head and neck surgeries, had physiotherapy or other complementary therapies in the last three months were excluded. The flow of the study program was documented following the CONSORT guidelines and displayed in (fig. 1).

The list of participants was compiled from the #####, and requests were sent to the participants via personal e-mail. Two orthopaedic surgeons with ten to fifteen years of

experience diagnosed the CgH patients after they consented to participate in the study. The research was carried out at the Department of Physical Therapy and Health Rehabilitation, #####, using the recommended study protocols.

2.3 Interventions

Certified physiotherapists having 10 – 15 years of experience in spinal manipulation for CgH provided the approved technique in each group. All the participants in the three groups had given their willingness to participate in the study after getting detailed information about the study protocol. The treatment consisted of a 10-minute hydro collator pack application to relax the muscles of the area around the neck and upper back. Following this, the participant's neck muscles and joints were assessed for any musculoskeletal dysfunction. After that, the participants were given the manipulation techniques as per the directions of the study protocol. Standardized treatment techniques were used for all the group participants to reduce intervention bias. The procedures of intervention and follow-up measurements were recorded in standardized forms. During the study period, the participants were asked to refrain from taking any other type of intervention, they received the concerned interventions 3 times per week for 4 weeks.

2.3.1 Spinal manipulation therapy

Peterson and Bergman defined SMT as a high-velocity low-amplitude thrust (HVLAT) technique. Four experienced physiotherapists having experience in SMT performed this technique after evaluation of each participant by physical examination and palpation. The therapist located the sites of abnormal changes in each vertebra and then manipulated the area following the study's recommendations. If any participant reported any new red flag signs or showed no signs for manipulation, such as no pain or musculoskeletal dysfunction, then the procedure was not performed.

2.3.1.1 Cervical spine manipulation (CSM)

To perform the C1-C2 cervical spine manipulation (CSM) the participant was instructed to lie down in a face-up position with upper and lower extremities kept aside relaxed. The head was kept in a neutral position and the treating therapist stands at the patient's head side and holds the chin of the patient with the right side hand. The therapist left hand holds the posterior aspect of the head and does two to three free rotatory movements. Afterwards, the therapist did

HVLAT technique in either the right or left direction based upon the symptoms informed by the patient (fig. 2). The manipulation was done first on the pain-free side and then on the painful side and the rotation range is limited by the target vertebra.[18]

2.3.1.2 Thoracic spine manipulation (TSM)

To perform the T1-T2 thoracic spine manipulation (TSM) the patient lays down in a face-up position with his arms crossed across his chest in a vertical direction, with elbows in the top position. The therapist stands beside the participant and faces towards the him. The treating therapist's left hand held the patient's both elbows together to apply downward force, while the right hand was kept under the spinous process of the target vertebra's lower vertebra with the thenar eminence and middle phalanx of the middle finger. The vertical downward thrust (anterior to posterior) was applied to the target vertebra by the therapist's left hand, and the thrust was adjusted by the right hand through pronation and radial deviation. Manipulation was performed by lowering the knees to generate thrust force while keeping the therapist's spine straight and head up (fig. 3).[15]

2.3.2 Conventional physiotherapy (CPT)

The participants of the CPT group received massage therapy for 15 minutes using Queen Helene, Cocoa Butter face & body cream, New York, USA. The patient lays down in a face-up position, with the posterior aspect of the head resting on a folded towel. The treating therapist stands by the patient's head side and uses the tips of the middle fingers of both hands to perform circular kneading on both sides of the C1 to C7 vertebra. This maneuver was repeated 3 times for each cervical vertebra, beginning from the C7 vertebra and working towards the C1 vertebra. The neck was then turned to the other side, and the same manoeuvre was repeated from insertion to origin on the sub-occipital and paravertebral muscles.[19]

Participants of all three groups were asked to perform neck isometric exercises three times per day, every day. The patient was asked to keep his hand over his forehead and resist the forward movement of his neck for 10 seconds and the same movement was repeated 15 times. Similarly, the patient was asked to keep the hand on the posterior and lateral sides of the head, and resist the backward and sideways movements of the neck. Also, static active stretching exercises for the upper trapezius, levator scapulae, scalene, and sternocleidomastoid muscles were taught to the patients, which was maintained for the 30s with 3 repetitions. The patients were instructed to keep doing this set of exercises after 4 weeks of various

intervention protocols and they were asked to maintain an exercise log book to check the treatment compliance.

2.4 Outcomes

All the outcome measures were recorded by a blinded physiotherapist, and the scores were entered in a data sheet. The scores were measured at the beginning of the study, after 4-weeks, 8-weeks, and at 6-months.

2.4.1 Primary outcome

CgH frequency: It is a self-administered outcome variable where the patient enters his CgH pain experience in a medical log book every evening to find the number of painful days in 4-weeks.[20]

2.4.2 Secondary outcome

CgH pain intensity: The pain intensity of CgH was assessed using an 11-point numerical pain rating scale (NPRS). Patients rated their typical level of pain status during the previous week on a 10 cm horizontal line, with one end 0 representing "no pain" and the other end 10 representing "worst pain imaginable."[21]

CgH disability: The Headache Impact Test (HIT) questionnaire is a valid and reliable instrument to assess the level of disability in CgH patients. It consists of six items: pain, social functioning, role functioning, vitality, cognitive functioning, and psychological distress. The score categories are no or mild disability (49 or less), moderate disability (50–55), severe disability (56–59), and complete disability (60–78).[22]

NP frequency: It is a self-administered outcome variable where the patient enters his neck pain experience in a medical log book every evening to find the number of painful days in 4-weeks.[20]

NP intensity: The pain intensity of neck pain was assessed using an 11-point numerical pain rating scale (NPRS). Patients rated their average pain intensity over the past week on a 10 cm horizontal line, with one end 0 representing "no pain" and the other end 10 representing "worst pain imaginable."[21]

NP pressure threshold: It is the lowest intensity at which a given stimulus is perceived as painful and it was measured using an instrument Algometer (Baseline, 22-pound dolorimeter,

ID, USA). The tip was placed over the fixed points on the back of the neck and assessed through palpation. It is a reliable and valid tool for determining pain threshold.[23]

Cervical flexion–rotation test (FRT): The cervical flexion–rotation test is done with the patient in a supine lying position. The therapist passively maintains the patient's neck into full flexion to relax the structures of the middle and lower cervical spine, then the patient's head is passively rotated in each direction while the flexed position is maintained and the range of motion is measured.[8]

Neck disability index (NDI): It is a reliable and valid self-reported questionnaire with ten items scored on a 0 to 5 scale. The grades of disability are determined based on the scores obtained, which are as follows: 10-29% mild; 30-49% moderate; 50-69% severe; 70% or more is a complete disability.[24]

Quality of life: The EQ-5D (Euro Qol 5D) is self-administered health related quality of life (HRQOL) questionnaire, which measures the five dimensions of quality of life. It includes mobility, self-care, usual activities, pain/discomfort, and anxiety/depression. It is used to assess the CgH patients' overall quality of life with a scale of 0 (worst) to 100 (best). [25]

2.5 Sample size

For calculating the number of subjects to be include in the study, the primary outcome measure CgH frequency in days was taken into consideration based on previous pilot study which found the effect of spinal manipulation in the treatment of CgH, with 10 subjects in each group. Using the G-Power software (version 3.1.9.2; Franz Faul, University of Kiel, Germany), assuming a two-sided $\alpha = 0.05$, and power ($1-\beta = 0.80$), to detect an effect size of 1.2 CgH days and mean difference of 4 CgH days (between groups) and a standard deviation of 0.5, an approximately 28 samples were required. In assuming 10% dropout, we enrolled 32 subjects in each group.

2.6 Blinding

Because of the experimental nature of the study methodology, it was not feasible to blind the treating therapist as well as the participants of the study. The therapists who assessed the outcome variables at baseline, 4-weeks, 8-weeks, and 6-months were blinded. Therefore, the therapist providing the treatment and the therapist measuring the data were different

individuals. In addition, the outcome-measuring therapist continued to be masked to the participant's groups at all-time intervals. Also, participants were asked not to discuss their treatment details with their peers or the outcome-measuring therapist. In addition, authors did not have access to information that could identify individual participants during or after data collection.

2.7 Statistical methods

The normality of study participants' demographic characteristics was analyzed through the Kolmogorov-Smirnov test. The outcome data were presented in the form of a mean and standard deviation with a 95% confidence interval. The effects of treatment at different time intervals were analyzed using a 3 × 4 linear mixed model analysis (LMM), with treatment groups (cervical spine manipulation, thoracic spine manipulation, and conventional physical therapy) and time intervals (baseline, four weeks, eight weeks, and at six months) and a statistical significance level of $\alpha=0.05$. All the statistical tests were done using GraphPad-Prism (version 9.1), Boston, MA, USA.

3. RESULTS

3.1 Participants

Out of the 130 participants screened, eight had a VAS score greater than 8, ten participants were having some sort of orthopaedic injuries, four participants were undergone joint surgeries, and twelve refused to involve in the research and were excluded. N=96 participants were chosen based on the eligibility criteria and allocated to one of the three groups. Two participants in the CSM and CMT groups, and three in the TSM group, did not complete the 4-week treatment program with a 6-month follow-up (fig. 1), and the study analysis assumed the intention to treat principle method. Compliance with follow-up data collection at 6 months was 93%, adherence to study protocols (e.g., number of visits) was 100%, and none of the participants in the three groups received any additional care that was not included in the three study interventions. At baseline, the demographic characters such as age (years), height (cm), weight (kg), and BMI (kg/m^2) scores did not report statistically significant variation between the groups ($p>0.05$). In all three groups, females (53-56%) are affected more than males. At baseline, the clinical variables also did not show any significant

difference between groups (p0.05). The clinical presentation of headache is more unilateral (78% - 84%) than bilateral, and the majority of CGH cases have associated neck pain (84% - 88%). (Table 1).

3.2 Primary outcome

The preliminary score on the primary outcome CgH frequency score among the CSM, TSM, and CPT groups showed no statistical variation ($p \geq 0.05$) which indicated the homogenous presence of study participants. The mean and standard deviation (SD) of the CgH frequency score between the three groups, at four-time periods, was shown in Table 2. The 3 × 4 linear mixed model (LMM) analysis reported statistically significant change in CgH frequency (p=0.012, $\eta p^2 = 0.092$). Over 4 weeks of different interventions, there is a significant change in CgH pain frequency level between the CSM (7.9; CI 95% 7.41 to 8.38), and TSM (4.7 CI 95% 4.21 to 5.18), groups (p=0.001). A similar improvement can be seen in 8-weeks and at 6-months' measurement. The post-hoc Bonferroni analysis and the standard mean difference showed more percentage of improvement in CgH pain frequency in the CSM group than TSM and CPT groups (fig. 4). The complete interpretation shows a slight leaning towards the CSM group than TSM and CPT group in CgH frequency.

3.3 Secondary outcomes

The preliminary score on the secondary outcome variables between the CSM, TSM, and CPT groups showed no statistical variation ($p \geq 0.05$) which indicated the homogenous presence of study participants. The mean and standard deviation (SD) of the secondary outcomes between the three groups, at four-time periods, was shown in Table 2. The 3 × 4 linear mixed model showed statistically significant variation in CgH pain intensity (p = 0.022, $\eta p^2 = 0.078$), CgH disability (p = 0.043, $\eta p^2 = 0.102$), neck pain frequency (p = 0.007, $\eta p^2 = 0.086$), neck pain intensity (p = 0.002, $\eta p^2 = 0.096$), neck pain threshold (p = 0.015, $\eta p^2 = 0.104$), flexion rotation test (Right and left) (p = 0.008, 0.006 $\eta p^2 = 0.091$, 0.074), neck disability index (p = 0.003, $\eta p^2 = 0.082$) and quality of life (p = 0.004, $\eta p^2 = 0.112$).

After 4 weeks of intervention, there are statistically significant variations in CgH pain intensity, CgH disability, NP (frequency, intensity, and threshold), FRT (Right and left), NDI, and QoL score between the CSM (2.9; CI 95% 2.71 to 3.08), (17.1; CI 95% 14.7 to 19.6) (12.7; CI 95% 12.08 to 13.31), (3.1; CI 95% 2.89 to 3.30), (-18.7; CI 95% -29.18 to -8.21), (-12.9, -12.31; CI 95% -16.12 to -9.67, -15.30 to -9.31), (23.64; CI 95% 20.55 to 26.72), (-32.5; CI

95% -35.89 to -29.10) and TSM (2.0; CI 95% 1.81 to 2.18), (8.8; CI 95% 6.3 to 11.2), (7.9; CI 95% 7.28 to 8.51), (1.8; CI 95% 1.59 to 2.00), (-8.8; CI 95% -19.28 to 1.68), (-5.91, -4.31; CI 95% -9.13 to -2.68, -7.30 to -1.31), (11.36; CI 95% 8.27 to 14.44), (-15.7; CI 95% -19.09 to -12.30) groups ($p<0.001$) respectively. The post hoc Bonferroni analysis and the standard mean difference showed more percentage of improvement in CgH pain frequency in the CSM group than TSM and CPT groups (fig. 4). The complete interpretation shows a slight leaning towards the CSM group than TSM and CPT group in all the secondary variables.

4. DISCUSSION

Despite having manual therapy reference in clinical practice guidelines (CPG) recommended by Ontario protocol for traffic injury management (OPTIMa) for the use of cervical and thoracic spine for patients with CgH, so far no randomized clinical trials (RCT) have been conducted to find the individual effects of CSM, TSM and conventional physiotherapy for CgH patients.[26] Based on our information, this is the first RCT conducted to investigate and compare the individual effects of cervical and thoracic spine manipulation, with conventional physiotherapy in CgH patients with 6-month follow-up period. The recent report shows that the lower cervical spine (C5, C6, and C7) and upper thoracic spine (T1 and T2) are known to be a more mobile and functional region in the human body, where stability is sacrificed and more prone to joint pathologies like spondylosis, spondylolisthesis, disc degeneration, disc bulge and herniation. Patients with such joint dysfunctions may experience unilateral or bilateral CgH, diffuse pain in the neck region, restricted neck movements, and disturbed activities of daily living (ADL).[27]

According to this study, after four weeks of intervention the cervical spine manipulation (CSM) group showed statistically significant changes in all the outcome measures in CgH patients. When compared to TSM and conventional physical therapy, CSM is more effective in managing CgH pain (MCID = 0.9, 9%) and associated neck pain (MCID = 1.3, 13%) at 6 months. CgH and neck pain frequency was also significantly lower in the CSM group (MCID = 3.2, 11%; 4.8, 16%) than in the TSM group, which was supported by Dunning JR et al.[16] So far, no studies have looked at the changes in CgH disability and neck disability, but this study looked at the benefits of cervical spine manipulation (MCID = 8.3, 20%; 12.28, 25%) over thoracic spine manipulation. However, these findings were contradicted by Borusiak et al. findings.[28] According to our findings, HVLAT technique in a cervical vertebra of a short

duration (less than 2 ms) promotes afferent nerve fibre activity via joint receptors. It improves the overall action and properties of the neck muscles by activating the alpha motor neuron.[29] It alters the sensory fiber activity by activating the joint receptors, thereby changing the α-motor neuron activity levels and subsequent muscle reaction. Because of the high mobility of the cervical spine, CSM can stimulate the receptors of deep neck muscles and sub-occipital muscles, which TSM is not able to do.[30] Other theories for the pain-modulating effects of cervical manipulation included biomechanical, vertebral (temporal summation), and neural (central descending pain inhibitory pathway) mechanisms which were noted by Bialosky JE et al and Haavik-Taylor H et al.[5,31,32]

Our findings also showed that thoracic spine manipulation improved both primary and secondary outcomes in CgH patients. TSM was also helpful in improving the pain parameters, functional disability and quality of life significantly. It was agreed by research by McDevitt AW et al[15] and suggests that TSM speeds up the healing process. Although several types of research have been conducted to find the effects of different types of spinal manipulation, the real mechanical and neuro-physiological alterations behind these changes have not yet been found and are unclear, but Bialosky et al. suggest that the effects may be due to potential neurophysiological and biomechanical effects, as well as possibly placebo effect.[31] According to Masaracchio M et al, TSM, impedes the excitation of pain-stimulating nerve fibres in the apophyseal joints, intervertebral discs, paraspinal muscles and ligaments, resulting in pain reduction and improved joint range of motion in mechanical neck pain. The same neuro-physiological changes would have a minor impact on sensory receptors, which relaxes the paraspinal muscles and decreases the pain status in CgH patients.[33] The little changes in the conventional physical therapy group on pain intensity and other outcome variables explained the analgesic effect of CPT on cervicogenic headache. It promotes the anti-nociceptive response by activating the opioid and oxytocin interaction through the release of neurotransmitters by stimulating the local nerves. Application of massage on the trigeminal-cervical area reduces inflammatory responses, reduces neural sensitivity and plays a significant function in decreasing the tension of the sub-occipital and para-vertebral cervical spine muscles, which is another important mechanism of physical therapy on CgH patients.[34,35] The findings of this trial would assist physiotherapists in making decisions to select the best manual therapy approach for CgH patients.

Limitations

The study had some limitations during its execution, which should be considered for future studies. First, the study included both genders, but the reports collected were not calculated

independently during the statistical analysis, these differences may influence the research reports. Second, it is impossible to ensure that the subjects completed the questionnaires daily rather than after a week or four weeks. Third, this study lacks a placebo group to determine the true effects of treatment groups. The beneficial effects of various manipulation techniques on pain and other symptoms in Cervicogenic headache were investigated. Finally, the treatment preference of physiotherapists and patients was not asked which could have affected the results. Future studies are recommended to find the biomechanical and biochemical mechanisms underlying the clinical and functional changes of these manipulation techniques in treating patients with CgH symptoms.

5. CONCLUSION

The reports of this current randomized clinical study found that cervical spine manipulation experienced significantly better improvements in pain parameters (intensity, frequency and threshold) functional disability and quality of life in CgH than thoracic spine manipulation and conventional physiotherapy. The report also provided strong evidence in the field of manual therapy for cervicogenic headache, a common and costly clinical condition.

List of abbreviations: CgH - Cervicogenic Headache, ROM - range of motion, FRT - flexion rotation test, ICHD - International Classification of Headache Disorders, CSM - cervical spine manipulation, TSM - thoracic spine manipulation, CMT - conventional massage therapy, HVLAT - high-velocity low amplitude thrust, NDI - Neck disability index, QOL - Quality of life

REFERENCES

1. AlQarni MA, Fayi KA, Al-Sharif MN, Siddiqui AF, Alhazzani AA. Prevalence of migraine and non-migraine headache and its relation with other diseases in the adults of Aseer Region, Saudi Arabia. *J Family Med Prim Care*. 2020;9(3):1567-1572.
2. Chaibi A, Russell MB. Manual therapies for cervicogenic headache: a systematic review. *J Headache Pain*. 2012;13(5):351-359.
3. Verma S, Tripathi M, Chandra PS. Cervicogenic Headache: Current Perspectives. *Neurol India*. 2021;69(Supplement):S194-S198.
4. Pourahmadi M, Mohseni-Bandpei MA, Keshtkar A, et al. Effectiveness of dry needling for improving pain and disability in adults with tension-type, cervicogenic, or migraine headaches: protocol for a systematic review. *Chiropr Man Therap*. 2019;27:43.

5. Lerner-Lentz A, O'Halloran B, Donaldson M, Cleland JA. Pragmatic application of manipulation versus mobilization to the upper segments of the cervical spine plus exercise for treatment of cervicogenic headache: a randomized clinical trial. *J Man Manip Ther*. 2021;29(5):267-275.
6. Sjaastad O, Fredriksen TA, Pfaffenrath V. Cervicogenic headache: diagnostic criteria. The Cervicogenic Headache International Study Group. Headache. 1998;38(6):442–5.
7. Hall T, Briffa K, Hopper D. Clinical evaluation of cervicogenic headache: a clinical perspective. *J Man Manip Ther*. 2008;16(2):73-80.
8. Hall TM, Briffa K, Hopper D, Robinson K. Comparative analysis and diagnostic accuracy of the cervical flexion-rotation test. *J Headache Pain*. 2010;11(5):391-397.
9. Bogduk N.Govind J., Cervicogenic headache: an assessment of the evidence on clinical diagnosis, invasive tests, and treatment. Lancet Neurol, 2009. 8(10): p. 959–68.
10. Page P. Cervicogenic headaches: an evidence-led approach to clinical management. *Int J Sports Phys Ther*. 2011;6(3):254-266.
11. Clarke TCBL, Stussman BJ, Barnes PM, Nahin RL. Trends in the use of complementary health approaches among adults: United States, 2002–2012. Natl Health Stat Rep. 2015;79:1–16.
12. Fernández-de-Las-Peñas C, Courtney CA. Clinical reasoning for manual therapy management of tension type and cervicogenic headache. *J Man Manip Ther*. 2014;22(1):44-50.
13. Haas M, Groupp E, Aickin M, Fairweather A, Ganger B, Attwood M, et al. Dose response for chiropractic care of chronic cervicogenic headache and associated neck pain: a randomized pilot study. J Manipulative Physiol Ther. 2004;27(9):547–53.
14. Nilsson N, Christensen HW, Hartvigsen J. The effect of spinal manipulation in the treatment of cervicogenic headache. *J Manipulative Physiol Ther*. 1997;20(5):326-330.
15. McDevitt AW, Cleland JA, Rhon DI, et al. Thoracic spine thrust manipulation for individuals with cervicogenic headache: a crossover randomized clinical trial. *J Man Manip Ther*. 2022;30(2):78-95.
16. Dunning JR, Butts R, Mourad F, Young I, Peñas CF, Hagins M et al. Upper cervical and upper thoracic manipulation versus mobilizationand exercise in patients withcervicogenic headache: a multi-center randomized clinical trial. BMC Musculoskeletal Disorders. 2016;17:64.
17. The International Classification of Headache Disorders, 3rd edition. Cephalalgia. 2018, 38(1):1–211.

18. Peterson DH, Bergmann T. Chiropractic technique: principles and procedures. 3rd ed. St. Louis: Mosby; 2010.
19. Diana Hopper, Yogita Bajaj, Chor Kei Choi, et al. A pilot study to investigate the short-term effects of specific soft tissue massage on upper cervical movement impairment in patients with cervicogenic headache. Journal of Manual and Manipulative Therapy 2013: 21(1); 18 – 24.
20. Bendtsen L, Bigal ME, Cerbo R, Diener HC, Holroyd K, Lampl C, Mitsikostas DD, Steiner TJ, Tfelt-Hansen P. International Headache Society Clinical Trials S: Guidelines for controlled trials of drugs in tension-type headache: second edition. Cephalalgia. 2010;30(1):1–16.
21. Jensen MP, Karoly P, Braver S. The measurement of clinical pain intensity: a comparison of six methods. Pain. 1986;27(1):117–26.
22. Kosinski M, Bayliss MS, Bjorner JB, Ware Jr JE, Garber WH, Batenhorst A, Cady R, Dahlof CG, Dowson A, Tepper S. A six-item short-form survey for measuring headache impact: the HIT-6. Qual Life Res Int J Qual Life Asp Treat Care Rehab. 2003;12(8):963–74.
23. Chesterton LS, Sim J, Wright CC, Foster NE. Interrater reliability of algometry in measuring pressure pain thresholds in healthy humans, using multiple raters. Clin J Pain. 2007;23(9):760–6.
24. Vernon H, Mior S. The neck disability index: a study of reliability and validity. J Manipulative Physiol Ther. 1991;14:7. 409–415.
25. Luo N, Johnson JA, Shaw JW, Feeny D, Coons SJ. Self-reported health status of the general adult U.S. population as assessed by the EQ-5D and Health Utilities Index. Med Care. 2005;43(11):1078–86.
26. Côté P, Yu H, Shearer HM, et al. Non-pharmacological management of persistent headaches associated with neck pain: A clinical practice guideline from the Ontario protocol for traffic injury management (OPTIMa) collaboration. *Eur J Pain*. 2019;23(6):1051-1070.
27. Anarte-Lazo E, Carvalho GF, Schwarz A, Luedtke K, Falla D. Differentiating migraine, cervicogenic headache and asymptomatic individuals based on physical examination findings: a systematic review and meta-analysis. *BMC Musculoskelet Disord*. 2021;22(1):755.
28. Borusiak P, Biedermann H, Bosserhoff S, Opp J. Lack of efficacy of manual therapy in children and adolescents with suspected cervicogenic headache: results of a prospective, randomized, placebo-controlled, and blinded trial. *Headache*. 2010;50(2):224-230.
29. Bolton PS, Budgell BS. Spinal manipulation and spinal mobilization influence different axial sensory beds. Med Hypotheses. 2006;66(2):258–62.

30. Cassidy JD, Lopes AA, Yong-Hing K. The immediate effect of manipulation versus mobilization on pain and range of motion in the cervical spine: a randomized controlled trial. J Manipulative Physiol Ther. 1992;15(9):570–5.

31. Bialosky JE, Bishop MD, Price DD, Robinson ME, George SZ. The mechanisms of manual therapy in the treatment of musculoskeletal pain: a comprehensive model. Man Ther. 2009;14(5):531–8. doi:10.1016/j.math.2008.09.001

32. Haavik-Taylor H, Murphy B. Cervical spine manipulation alters sensorimotor integration: a somatosensory evoked potential study. Clin Neurophysiol. 2007;118(2):391–402. doi:10.1016/j.clinph.2006.09.014

33. Masaracchio M, Kirker K, States R, Hanney WJ, Liu X, Kolber M. Thoracic spine manipulation for the management of mechanical neck pain: A systematic review and meta-analysis. PLoS One. 2019 Feb 13;14(2):e0211877.

34. Panneton WM, Gan Q, Livergood RS. A trigeminoreticular pathway: implications in pain. PloS one. 2011; 6: e24499.

35. Youssef EF, Shanb AS. Mobilization versus massage therapy in the treatment of cervicogenic headache: a clinical study. *J Back Musculoskelet Rehabil*. 2013;26(1):17-24.

Table 1: Demographic details of CSM, TSM and CPT groups.

Sr. No	Variable		CSM	TSM	CPT	p-value
**1**	Age (year)	-	35.6 ± 3.8	34.8 ± 3.2	36.2 ± 3.7	0.295*
**2**	Gender	Male	14 (44%)	15 (47%)	14 (44%)	-
		Female	18 (56%)	17 (53%)	18 (56%)	-
**3**	Height (cm)	-	164.6 ± 3.8	163.5 ± 4.1	165.3 ± 4.4	0.215*
**4**	Weight (m)	-	72.92 ± 4.2	71.21 ± 4.6	73.83 ± 4.5	0.061*
**5**	BMI (Kg/m^2)	-	24.3 ± 2.23	24.5 ± 1.92	23.8 ± 2.01	0.378*
**6**	CgH duration (year)	-	6.6 ± 2.9	6.8 ± 3.1	5.9 ± 3.2	0.471*
**7**	CgH frequency (per day)	-	0.74 ± 0.15	0.72 ± 0.11	0.69 ± 0.12	0.293*
**8**	CgH intensity(0-10)	-	6.9 ± 1.4	7.2 ± 1.3	7.3 ± 1.3	0.461*
**9**	Headache	Unilateral	26 (81%)	25 (78%)	27 (84%)	-
		Bilateral	6 (19%)	7 (12%)	5 (16%)	-
**10**	Neck pain	Yes	28 (88%)	27 (84%)	28 (88%)	-
		No	4 (12%)	5 (16%)	4(12%)	-

* Non Significant, CSM – Cervical spine manipulation, TSM – Thoracic spine manipulation, CMT- Conventional physiotherapy, BMI – Body mass index, CgH – Cervicogenic Headache

Table 2: Pre and post mean and SD of outcome measures of CSM, TSM and CPT groups.

Sr.No	Variable		CSM	TSM	CPT	Group × Time p-value
**1**	**CgH Frequency (no of days per 4 weeks)**	Base line	16.8 ± 1.8	17.2 ± 1.9	17.4 ± 1.7	**0.012****
		4 weeks	11.2 ± 1.4	14.1 ± 1.6	15.8 ± 1.4	
		8 weeks	6.2 ± 0.9	9.5 ± 0.9	13.4 ± 1.1	$\eta p^2 = 0.092$
		6 months	2.9 ± 0.5	6.1 ± 0.7	10.8 ± 1.1	
**2**	**CgH Pain intensity (0 -10)**	Base line	7.2 ± 0.8	6.8 ± 0.7	7.1 ± 0.7	**0.022****
		4 weeks	4.1 ± 0.5	5.2 ± 0.5	6.2 ± 0.5	
		8 weeks	2.8 ± 0.4	4.1 ± 0.4	5.1 ± 0.4	$\eta p^2 = 0.078$
		6 months	0.8 ± 0.2	1.7 ± 0.3	3.7 ± 0.4	
**3**	**CgH Disability**	Base line	57.88 ± 6.5	57.21 ± 6.8	56.91 ± 5.9	**0.043****
		4 weeks	45.73 ± 5.5	49.38 ± 5.6	52.38 ± 5.3	
		8 weeks	36.41 ± 4.3	41.73 ± 5.1	49.67 ± 4.5	$\eta p^2 = 0.102$
		6 months	31.19 ± 3.8	39.54 ± 4.5	48.37 ± 4.1	
**4**	**Neck pain frequency (no of days per 4 weeks)**	Base line	23.9 ± 3.2	23.6 ± 3.4	22.9 ± 3.6	**0.007****
		4 weeks	15.8 ± 2.2	18.5 ± 2.4	20.4 ± 2.3	
		8 weeks	9.3 ± 1.6	12.3 ± 1.8	18.2 ± 1.5	$\eta p^2 = 0.086$
		6 months	3.4 ± 0.4	8.2 ± 0.9	16.1 ± 1.5	
**5**	**Neck pain intensity (0 -10)**	Base line	7.2 ± 0.6	7.1 ± 0.6	6.9 ± 0.5	**0.002****
		4 weeks	4.8 ± 0.5	6.2 ± 0.6	6.1 ± 0.5	
		8 weeks	2.6 ± 0.4	4.2 ± 0.5	5.1 ± 0.4	$\eta p^2 = 0.096$
		6 months	0.6 ± 0.2	1.9 ± 0.4	3.7 ± 0.4	
**6**	**Neck pain threshold**	Base line	262.1 ± 21.6	261.3 ± 22.3	262.7 ± 22.5	**0.015****
		4 weeks	268.5 ± 20.2	266.3 ± 21.8	263.5 ± 21.6	
		8 weeks	274.5 ± 18.3	272.4 ± 19.4	267.8 ± 19.6	$\eta p^2 = 0.104$
		6 months	288.2 ± 17.3	278.3 ± 18.2	269.5 ± 17.3	
**7**	**Flexion rotation test (Right side)**	Base line	25.18 ± 7.4	25.12 ± 7.3	26.01 ± 7.2	**0.008****
		4 weeks	31.76 ± 6.3	30.19 ± 6.3	27.12 ± 6.5	
		8 weeks	39.73 ± 5.6	35.16 ± 5.4	31.42 ± 6.1	$\eta p^2 = 0.091$
		6 months	45.21 ± 5.3	38.22 ± 5.1	32.31 ± 5.8	
**8**	**Flexion rotation test (Left side)**	Base line	24.93 ± 6.2	24.72 ± 6.1	24.88 ± 6.3	**0.006****
		4 weeks	33.12 ± 5.9	27.93 ± 5.9	26.63 ± 5.8	
		8 weeks	37.81 ± 5.2	32.53 ± 5.5	29.92 ± 5.1	$\eta p^2 = 0.074$
		6 months	44.23 ± 5.1	36.23 ± 5.1	31.92 ± 4.9	
**9**	**Neck Disability Index**	Base line	51.01 ± 11.1	51.25 ± 10.8	50.97 ± 11.2	**0.003****
		4 weeks	37.36 ± 9.2	43.34 ± 9.8	45.32 ± 10.2	
		8 weeks	24.83 ± 6.1	31.32 ± 6.2	38.53 ± 6.9	$\eta p^2 = 0.082$
		6 months	11.28 ± 3.4	23.56 ± 5.4	34.92 ± 6.3	
**10**	**Quality of life**	Base line	24.9 ± 4.5	25.1 ± 3.9	24.8 ± 4.2	**0.004****
		4 weeks	48.3 ± 4.8	36.3 ± 4.8	34.9 ± 4.3	
		8 weeks	64.5 ± 5.5	55.3 ± 5.1	40.1 ± 4.6	$\eta p^2 = 0.112$
		6 months	79.2 ± 6.2	62.4 ± 5.9	46.7 ± 4.9	

** Significant, CSM – Cervical spine manipulation, TSM – Thoracic spine manipulation, CPT- Conventional physiotherapy, CgH – Cervicogenic Headache

Table 3: Pre and post mean difference and confidence interval (upper limit and lower limit) scores of CSM, TSM and CPT groups.

Variable / Time		Baseline	4 weeks	8 weeks	6 months
		Mean difference (95% CI)			
**CgH Frequency**	**CSM × TSM**	0.4 (-0.67 to 1.47)	2.9 (2.02 to 3.77)	3.3 (2.72 to 3.87)	3.2 (2.71 to 3.68)
	**P – value**	0.649	0.001	0.001	0.001
	**CSM × CPT**	0.6 (-0.47 to 1.67)	4.6 (3.72 to 5.47)	7.2 (6.62 to 7.77)	7.9 (7.41 to 8.38)
	**P – value**	0.381	0.001	0.688	0.874
	**TSM × CPT**	0.2 (-0.87 to 1.27)	1.7 (0.82 to 2.57)	3.9 (3.32 to 4.47)	4.7 (4.21 to 5.18)
	**P – value**	0.897	0.001	0.001	0.004
**CgH Pain Intensity**	**CSM × TSM**	-0.4 (-0.83 to 0.03)	1.1 (0.80 to 1.39)	1.3 (1.06 to 1.53)	0.9 (0.71 to 1.08)
	**P – value**	0.080	0.001	0.001	0.001
	**CSM × CPT**	-0.1 (-0.53 to 0.33)	2.1 (1.80 to 2.39)	2.3 (2.06 to 2.53)	2.9 (2.71 to 3.08)
	**P – value**	0.849	0.001	0.003	0.874
	**TSM × CPT**	0.3 (-0.13 to 0.73)	1.0 (0.70 to 1.29)	1.0 (0.76 to 1.23)	2.0 (1.81 to 2.18)
	**P – value**	0.237	0.001	0.001	0.108
**CgH Disability**	**CSM × TSM**	-0.6 (-4.48 to 3.14)	3.6 (0.39 to 6.90)	5.3 (2.55 to 8.08)	8.3 (5.88 to 10.81)
	**P – value**	0.908	0.024	0.001	0.001
	**CSM × CPT**	-0.9 (-4.78 to 2.84)	6.6 (3.39 to 9.90)	13.2 (10.49 to 16.02)	17.1 (14.7 to 19.6)
	**P – value**	0.817	0.000	0.001	0.001
	**TSM × CPT**	-0.3 (-4.11 to 3.51)	3.00 (-0.25 to 6.25)	7.9 (5.17 to 10.70)	8.8 (6.3 to 11.2)
	**P – value**	0.980	0.774	0.001	0.001
**Neck pain frequency**	**CSM × TSM**	-0.3 (-2.32 to 1.7)	2.7 (1.32 to 4.07)	3.0 (2.02 to 3.97)	4.8 (4.18 to 5.41)
	**P – value**	0.933	0.001	0.001	0.001
	**CSM × CPT**	-1.0 (-3.02 to 1.02)	4.6 (3.22 to 5.97)	8.9 (7.92 to 8.97)	12.7 (12.08 to 13.31)
	**P – value**	0.470	0.001	0.002	0.874
	**TSM × CPT**	-0.7 (-2.72 to 1.32)	1.9 (0.52 to 3.27)	5.9 (4.92 to 6.87)	7.9 (7.28 to 8.51)
	**P – value**	0.690	0.003	0.001	0.746
**Neck pain intensity**	**CSM × TSM**	-0.1 (-0.43 to 0.23)	1.4 (1.08 to 1.71)	1.6 (1.34 to 1.85)	1.3 (1.09 to 1.50)
	**P – value**	0.762	0.001	0.001	0.001
	**CSM × CPT**	-0.3 (-0.63 to 0.03)	1.3 (0.98 to 1.61)	2.5 (2.24 to 2.75)	3.1 (2.89 to 3.30)
	**P – value**	0.093	0.001	0.001	0.873
	**TSM × CPT**	-0.2 (-0.53 to 0.13)	-0.1 (-0.41 to 0.21)	0.9 (0.64 to 1.15)	1.8 (1.59 to 2.00)
	**P – value**	0.341	0.736	0.001	0.001
**Neck pain threshold**	**CSM × TSM**	-0.8 (-13.98 to 12.38)	-2.2 (-14.83 to 10.43)	-2.1 (-13.47 to 9.27)	-9.9 (-20.38 to 0.58)
	**P – value**	0.988	0.909	0.899	0.068
	**CSM × CPT**	0.6 (-12.58 to 13.78)	-5 (-17.63 to 7.63)	-6.7 (-18.07 to 4.67)	-18.7 (-29.18 to -8.21)
	**P – value**	0.993	0.614	0.343	0.000
	**TSM × CPT**	1.4 (-11.78 to 14.58)	-2.8 (15.43 to 9.83)	-4.6 (-15.97 to 6.77)	-8.8 (-19.28 to 1.68)
	**P – value**	0.965	0.857	0.602	0.118
**Flexion rotation test (Right side)**	**CSM × TSM**	-0.06 (-4.40 to 4.28)	-1.5 (-5.36 to 2.22)	-4.5 (-7.9 to -1.1)	-6.9 (-10.21 to -3.76)
	**P – value**	0.980	0.587	0.005	0.001
	**CSM × CPT**	0.83 (-3.51 to 5.17)	-4.64 (-8.43 to -0.84)	-8.3 (-11.7 to -4.91)	-12.9 (-16.12 to -9.67)
	**P – value**	0.892	0.012	0.001	0.001
	**TSM × CPT**	0.89 (-3.45 to 5.23)	-3.07 (-6.86 to 0.72)	-3.74 (-7.13 to -0.34)	-5.91 (-9.13 to -2.68)
	**P – value**	0.877	0.136	0.027	0.001
**Flexion rotation test (Left side)**	**CSM × TSM**	-0.21 (-3.90 to 3.48)	-5.19 (-8.68 to -1.69)	-5.2 (-8.41 to -2.14),	-8.0 (-10.99 to -5.00)
	**P – value**	0.989	0.001	0.001	0.001
	**CSM × CPT**	-0.05 (-3.74 to 3.64)	-6.4 (-9.98 to -2.99)	-7.8 (-11.02 to -4.75)	-12.31 (-15.30 to -9.31)
	**P – value**	1.032	0.001	0.001	0.001
	**TSM × CPT**	0.16 (-3.53 to 3.85)	-1.3 (-4.79 to 2.19)	-2.61 (-5.74 to 0.52)	-4.31 (-7.30 to -1.31)
	**P – value**	0.994	0.650	0.122	0.002
**Neck Disability Index**	**CSM × TSM**	0.24 (-6.3 to 6.81)	5.98 (0.17 to 11.78)	6.49 (2.67 to 10.30)	12.28 (9.19 to 15.36)
	**P – value**	0.993	0.041	0.001	0.001
	**CSM × CPT**	-0.04 (-6.61 to 6.53)	7.96 (2.15 to 13.76)	13.7 (9.88 to 17.51)	23.64 (20.55 to 26.72)
	**P – value**	0.803	0.004	0.001	0.001
	**TSM × CPT**	-0.28 (-6.85 to 6.29)	1.98 (-3.82 to 7.78)	7.21 (3.39 to 11.02)	11.36 (8.27 to 14.44)
	**P – value**	0.994	0.696	0.001	0.001
**Quality of life**	**CSM × TSM**	0.20 (-2.30 to 2.70)	-12.0 (-14.76 to -9.23)	-9.2 (-12.2 to -6.1)	-16.8 (-20.19 to -13.40)
	**P – value**	0.980	0.001	0.001	0.001
	**CSM × CPT**	-0.10 (-2.60 to 2.40)	-13.4 (-16.16 to -10.63)	-24.4 (-27.4 to -21.37)	-32.5 (-35.89 to -29.10)
	**P – value**	0.996	0.001	0.001	0.002
	**TSM × CPT**	-0.30 (-2.80 to 2.20)	-1.4 (-4.16 to 1.36)	-15.20 (-18.22 to -12.17)	-15.7 (-19.09 to -12.30)
	**P – value**	0.956	0.452	0.001	0.001

CSM – Cervical spine manipulation, TSM – Thoracic spine manipulation, CPT- Conventional physiotherapy, CgH – Cervicogenic Headache

Figure legends:

Figure 1: Flow chart showing the study details.

Figure 2: Figure showing the cervical spine manipulation.

Figure 3: Figure showing the thoracic spine manipulation.

Figure 4: Pre and post outcome measures of CSM, TSM and CPT groups.

CONSORT flow chart

Click here to access/download;Figure;Figure 1.jpg

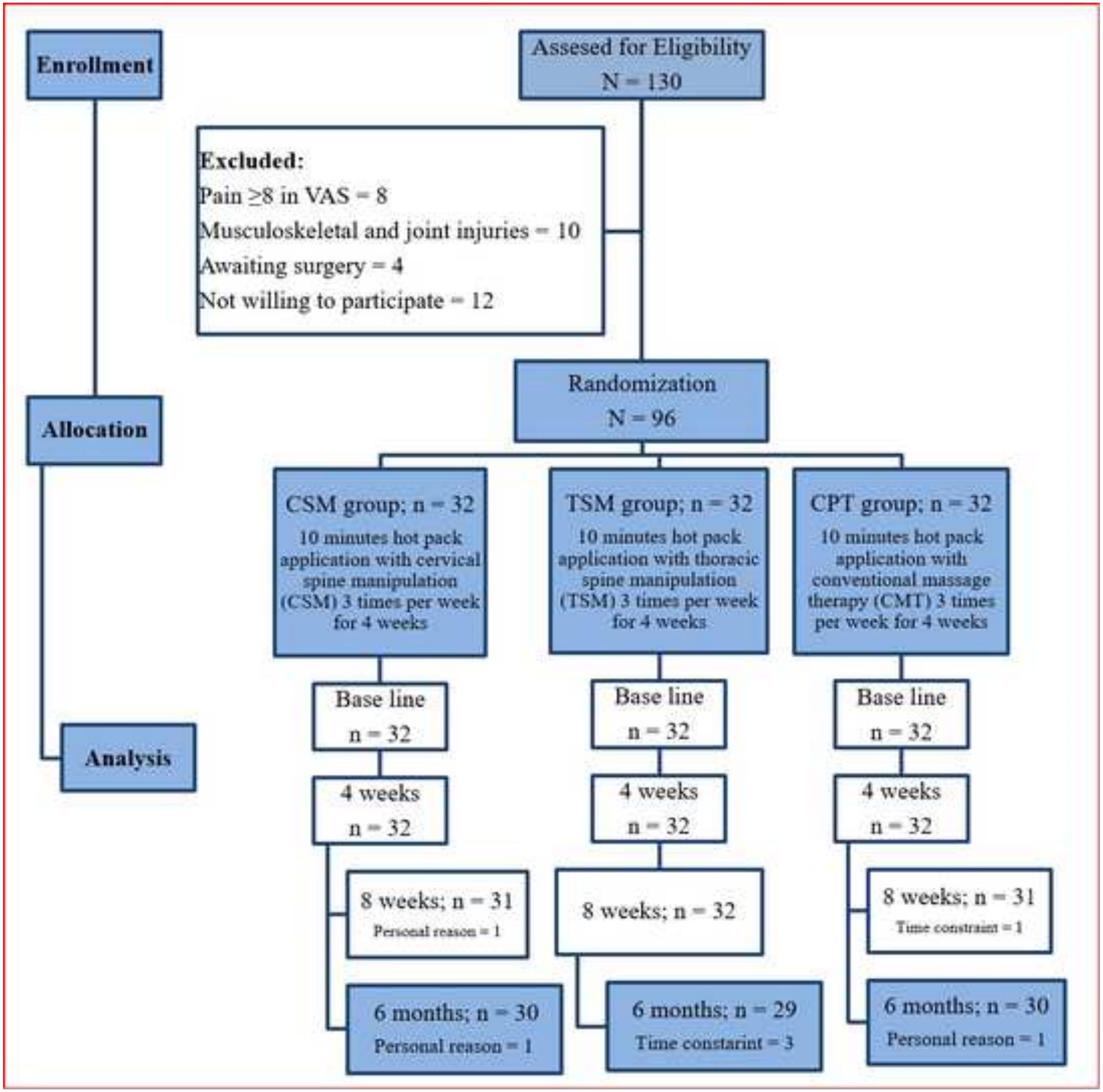

Figure 2

Click here to access/download;Figure;Figure 2.jpeg

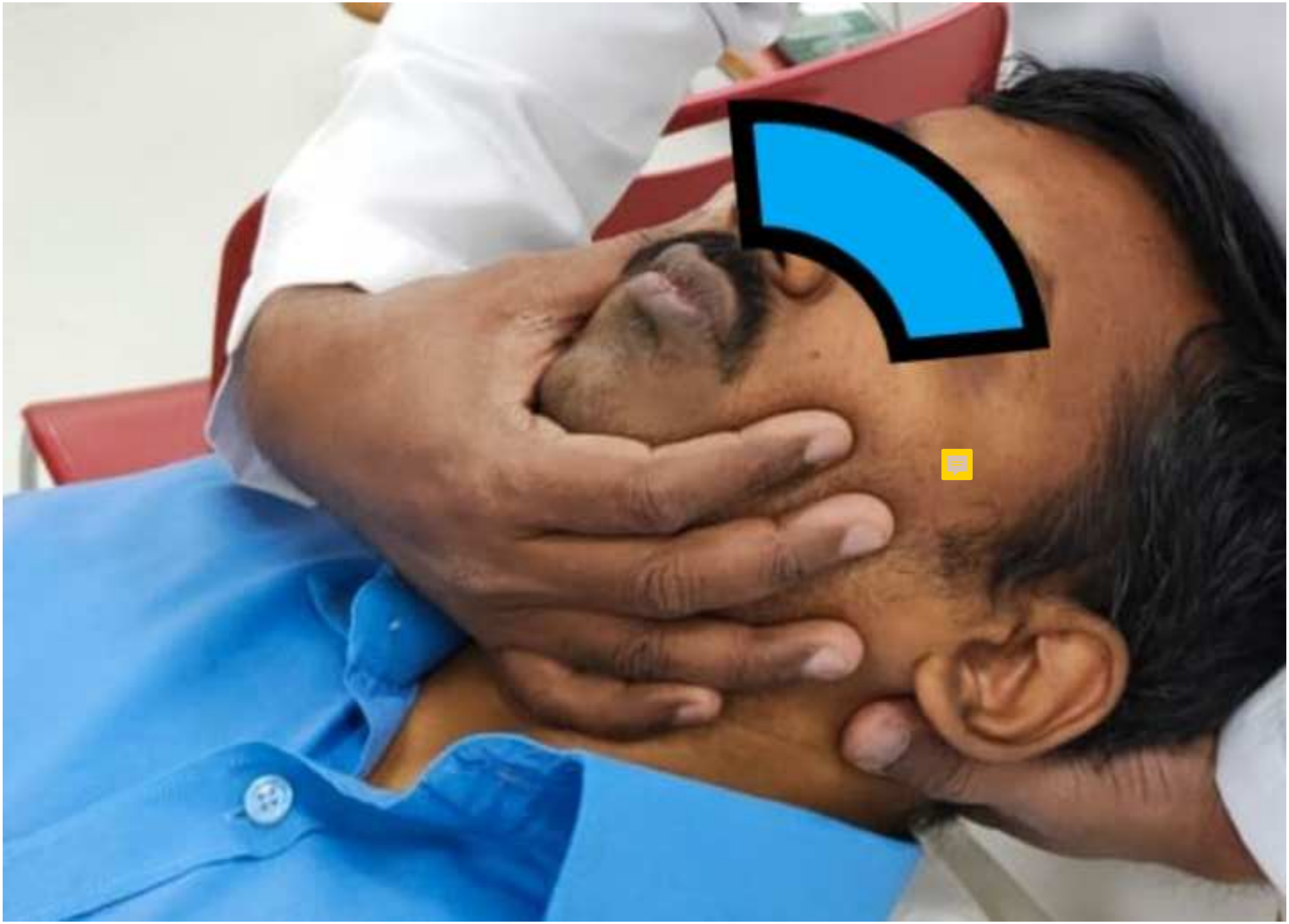

Figure 3

Click here to access/download;Figure;Figure 3.jpeg

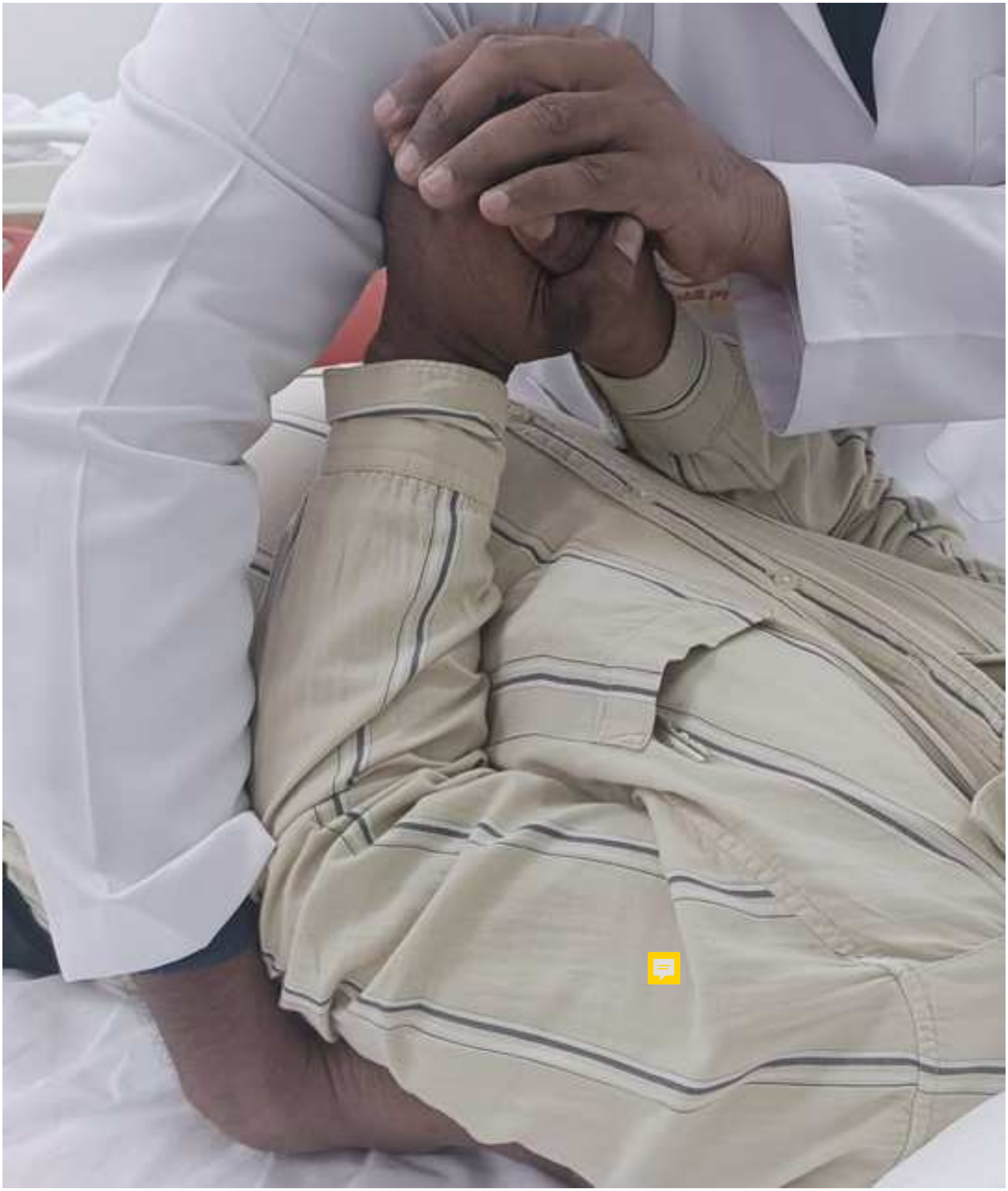

Figure 4

Click here to access/download;Figure;Figure 4.jpg

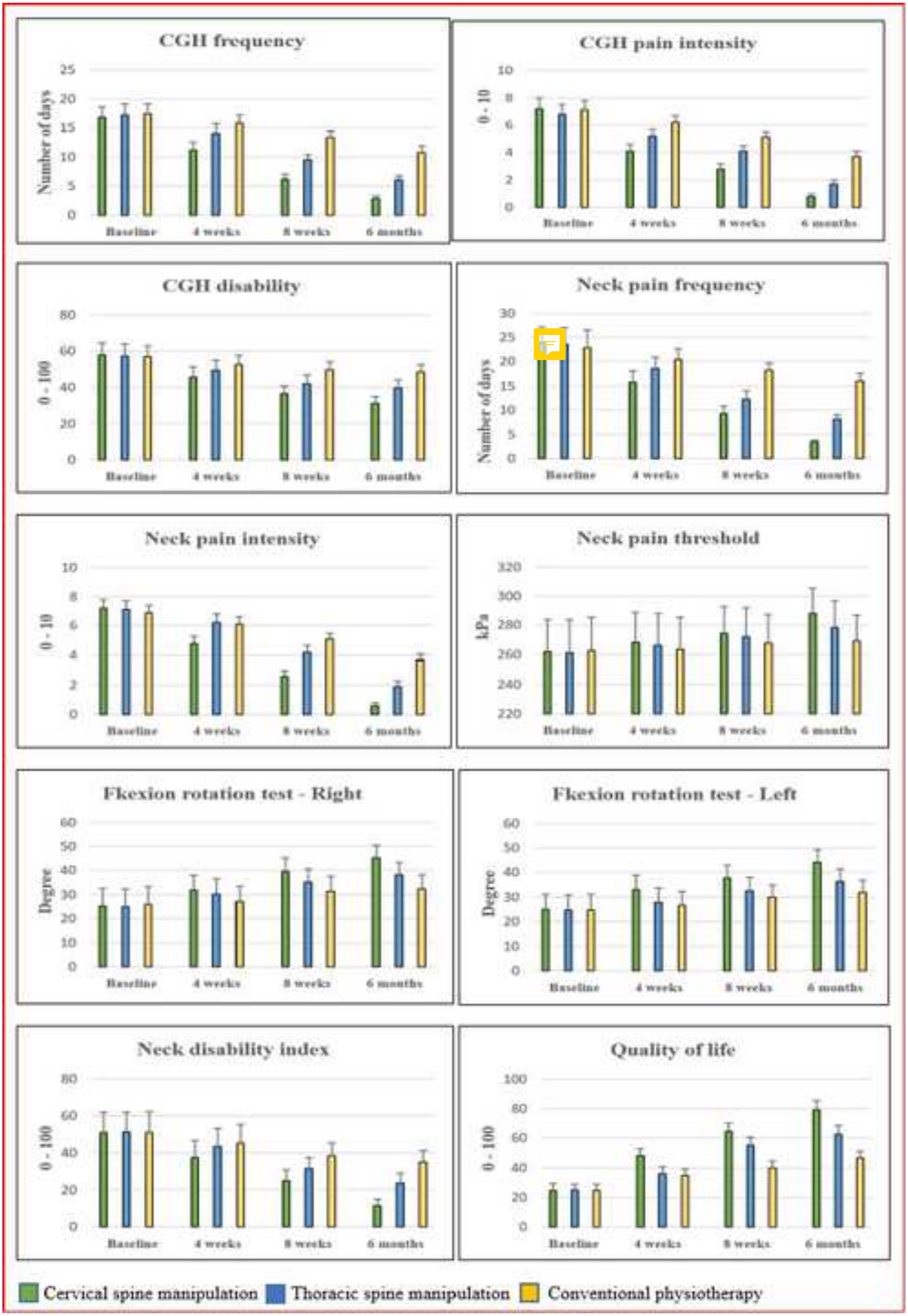

CONSORT Check list

Click here to access/download
Supporting Information
Consort 2010 checklist.docx

Trial Protocol

Click here to access/download
Supporting Information
Trial protocol.docx

Ethical approval

Click here to access/download

Other

Ethical approval.pdf
