# Supplementary material for: Comparative effectiveness of cervical vs thoracic spinal-thrust manipulation for care of cervicogenic headache: A randomized controlled trial
Source: PLoS One. 2024 Mar 29;19(3):e0300737. doi: 10.1371/journal.pone.0300737 (PMC10980233; doi:10.1371/journal.pone.0300737)
Supplement: Attachment — Submitted filename: PONE-D-23-12627_R1_reviewer_Redacted.pdf [file pone.0300737.s006.pdf]

PLOS ONE

Comparative effectiveness of cervical vs thoracic spinal-thrust manipulation for care of cervicogenic headache: a randomized controlled trial.

--Manuscript Draft--

**Manuscript Number:**	PONE-D-23-12627R1
**Article Type:**	Clinical Trial
**Full Title:**	Comparative effectiveness of cervical vs thoracic spinal-thrust manipulation for care of cervicogenic headache: a randomized controlled trial.
**Short Title:**	Manipulation techniques in Cervicogenic Headache.
**Corresponding Author:**	Gopal Nambi, PhD<br>Prince Sattam bin Abdulaziz University College of Applied Medical Sciences<br>Al Kharj, SAUDI ARABIA
**Keywords:**	Cervicogenic headache; Cervical spine; thoracic spine; manipulation; Physiotherapy
**Abstract:**	Background: There is ample evidence supporting the use of different manipulative therapy techniques for Cervicogenic Headache (CgH). However, no technique can be singled as the best available treatment for patients with CgH. Therefore, the objective of the study is to find and compare the clinical effects of cervical spine over thoracic spine manipulation and conventional physiotherapy in patients with CgH.<br>Design, Setting, Participants : It is a prospective, randomized controlled study conducted between July 2020 and January 2023 at the University hospital. N=96 eligible patients with CgH were selected based on selection criteria and they were divided into cervical spine manipulation (CSM; n=32), thoracic spine manipulation (TSM; n=32) and conventional physiotherapy (CPT; n=32) groups, and they received the respective treatment for four weeks. Primary (CgH frequency) and secondary (CgH pain intensity, CgH disability, neck pain frequency, neck pain intensity, neck pain threshold, flexion rotation test (right and left), neck disability index and quality of life) scores were measured. The effects of treatment at various intervals were analyzed using a 3 × 4 linear mixed model analysis (LMM), with treatment group (cervical spine manipulation, thoracic spine manipulation, and conventional physiotherapy) and time intervals (baseline, 4 weeks, 8 weeks, and 6 months), and the statistical significance level was set at $P < 0.05$.<br>Results: The baseline demographic and clinical variables did not show any significance difference ($p>0.05$). The reports of the CSM, TSM and CPT group were compared between the groups. Four weeks following treatment CSM group showed more significant changes in primary (CgH frequency) and secondary (CgH pain intensity, CgH disability, neck pain frequency, pain intensity, pain threshold, flexion rotation test (Right and left), neck disability index and quality of life) than TSM and CPT groups ($p=0.001$). The same gradual improvement was seen in CSM group when compared to TSM and CPT groups ($p=0.001$) in the above variables at 8 weeks and at 6 months follow up.<br>Conclusion: The reports of the current randomized clinical study found that CSM resulted in significantly better improvements in pain parameters (intensity, frequency and threshold) functional disability and quality of life in patients with CgH than thoracic spine manipulation and conventional physiotherapy.
**Order of Authors:**	Gopal Nambi, PhD
	Mshari Alghadier, PhD
	Mudathir Mohamedahmed Eltayeb, PhD
	Osama R. Aldhafian, MD
	Ayman K. Saleh, MD
	Nesreen Alsanousi, PhD
	Alaa Jameel A. Albarakati, MD
	Mohamed A. Omar, MD

Powered by Editorial Manager® and ProduXion Manager® from Aries Systems Corporation

	Mohamed Nagah Ahmed Ibrahim, MD
	Abdehamid A. Attallah, MD
	Mohammed Abdelgwad Ismail, MD
	Mohamed Elfeshawy, PhD
**Response to Reviewers:**	Dear Editor and reviewers,<br>Thank you for your recent correspondence regarding our paper on 'Comparative effectiveness of cervical vs thoracic spinal-thrust manipulation for care of cervicogenic headache: a randomized controlled trial." We would like to thank you for the opportunity to submit a revised manuscript. We appreciate all the comments, suggestions, and positive criticism raised by the Editor and the reviewers which were of great value, and certainly helped us to improve the quality of our manuscript. We made all possible efforts to properly address and/or reply to the comments raised. Additionally, a complete review of the article was done for grammar and readability. A significant number of modifications were made to the article after several reviews. No large disagreements were made with the reviewer's comments. The authors of this paper greatly thank each of the reviewers for spending time and provided such a constructive comments and fantastic input. The authors greatly appreciate the work of all the reviewers as well as the editors on this manuscript. Many points were a great addition to the quality of this article. Here is a corrected manuscript with a point-by-point response to comments from the reviewers. If any additional corrections can be corrected in the further rounds of revisions.<br>Regards<br>Dr. Gopal
**Additional Information:**	
**Question**	**Response**
**Financial Disclosure**<br><br>Enter a financial disclosure statement that describes the sources of funding for the work included in this submission. Review the submission guidelines for detailed requirements. View published research articles from *PLOS ONE* for specific examples.<br><br>This statement is required for submission and **will appear in the published article** if the submission is accepted. Please make sure it is accurate.	This study is supported via funding from Prince Sattam bin Abdulaziz University project number (PSAU/2023/R/1444). There was no additional external funding received for this study.

**Unfunded studies**<br>Enter: *The author(s) received no specific funding for this work.*<br><br>**Funded studies**<br>Enter a statement with the following details:<br>• Initials of the authors who received each award<br>• Grant numbers awarded to each author<br>• The full name of each funder<br>• URL of each funder website<br>• Did the sponsors or funders play any role in the study design, data collection and analysis, decision to publish, or preparation of the manuscript?<br>• **NO** - Include this sentence at the end of your statement: *The funders had no role in study design, data collection and analysis, decision to publish, or preparation of the manuscript.*<br>• **YES** - Specify the role(s) played.<br><br>* typeset	
**Competing Interests**<br><br>Use the instructions below to enter a competing interest statement for this submission. On behalf of all authors, disclose any competing interests that could be perceived to bias this work—acknowledging all financial support and any other relevant financial or non-financial competing interests.<br><br>This statement is required for submission and **will appear in the published article** if the submission is accepted. Please make sure it is accurate and that any funding sources listed in your Funding Information later in the submission form are also declared in your Financial Disclosure statement.<br><br>View published research articles from *PLOS ONE* for specific examples.	The authors have declared that no competing interests exist.

Powered by Editorial Manager® and ProduXion Manager® from Aries Systems Corporation

**NO authors have competing interests**<br><br>Enter: *The authors have declared that no competing interests exist.*<br><br>**Authors with competing interests**<br><br>Enter competing interest details beginning with this statement:<br><br>*I have read the journal's policy and the authors of this manuscript have the following competing interests: [insert competing interests here]*<br><br>* typeset	
**Ethics Statement**<br><br>Enter an ethics statement for this submission. This statement is required if the study involved:<br><br>• Human participants<br>• Human specimens or tissue<br>• Vertebrate animals or cephalopods<br>• Vertebrate embryos or tissues<br>• Field research<br><br>Write "N/A" if the submission does not require an ethics statement.<br><br>General guidance is provided below. Consult the submission guidelines for detailed instructions. **Make sure that all information entered here is included in the Methods section of the manuscript.**	This study was designed and conducted in accordance with the principles of the Declaration of Helsinki and it has been approved by the Department Ethics Committee, Prince Sattam bin Abdulaziz University, Saudi Arabia with an ethical approval number RHPT/019/042. The participants were informed about the harms and benefits of the research through an information form. Subjects who consented to participate in the study were selected for the study and the written informed consent was obtained from all subjects.

Powered by Editorial Manager® and ProduXion Manager® from Aries Systems Corporation

**Format for specific study types**<br><br>**Human Subject Research (involving human participants and/or tissue)**<br>• Give the name of the institutional review board or ethics committee that approved the study<br>• Include the approval number and/or a statement indicating approval of this research<br>• Indicate the form of consent obtained (written/oral) or the reason that consent was not obtained (e.g. the data were analyzed anonymously)<br><br>**Animal Research (involving vertebrate animals, embryos or tissues)**<br>• Provide the name of the Institutional Animal Care and Use Committee (IACUC) or other relevant ethics board that reviewed the study protocol, and indicate whether they approved this research or granted a formal waiver of ethical approval<br>• Include an approval number if one was obtained<br>• If the study involved *non-human primates*, add *additional details* about animal welfare and steps taken to ameliorate suffering<br>• If anesthesia, euthanasia, or any kind of animal sacrifice is part of the study, include briefly which substances and/or methods were applied<br><br>**Field Research**<br><br>Include the following details if this study involves the collection of plant, animal, or other materials from a natural setting:<br>• Field permit number<br>• Name of the institution or relevant body that granted permission	
**Data Availability**<br><br>Authors are required to make all data underlying the findings described fully available, without restriction, and from the time of publication. PLOS allows rare exceptions to address legal and ethical concerns. See the PLOS Data Policy and FAQ for detailed information.	Yes - all data are fully available without restriction

A Data Availability Statement describing where the data can be found is required at submission. Your answers to this question constitute the Data Availability Statement and **will be published in the article**, if accepted.<br><br>**Important:** Stating 'data available on request from the author' is not sufficient. If your data are only available upon request, select 'No' for the first question and explain your exceptional situation in the text box.<br><br>Do the authors confirm that all data underlying the findings described in their manuscript are fully available without restriction?	
**Describe where the data may be found in full sentences. If you are copying our sample text, replace any instances of XXX with the appropriate details.**<br><br>• If the data are **held or will be held in a public repository**, include URLs, accession numbers or DOIs. If this information will only be available after acceptance, indicate this by ticking the box below. For example: *All XXX files are available from the XXX database (accession number(s) XXX, XXX.*).<br>• If the data are all contained **within the manuscript and/or Supporting Information files**, enter the following: *All relevant data are within the manuscript and its Supporting Information files.*<br>• If neither of these applies but you are able to provide **details of access elsewhere**, with or without limitations, please do so. For example:<br><br>*Data cannot be shared publicly because of [XXX]. Data are available from the XXX Institutional Data Access / Ethics Committee (contact via XXX) for researchers who meet the criteria for access to confidential data.*<br><br>*The data underlying the results presented in the study are available from (include the name of the third party*	The minimal data set is available as a supporting file.

and contact information or URL).<br>• This text is appropriate if the data are owned by a third party and authors do not have permission to share the data.<br><br>* typeset	
Additional data availability information:	Tick here if your circumstances are not covered by the questions above and you need the journal's help to make your data available.

Manuscript
Click here to access/download;Manuscript;Final manuscript - R1.docx

Title: Comparative effectiveness of cervical vs thoracic spinal-thrust manipulation for care of cervicogenic headache: a randomized controlled trial.

Running Title: Manipulation techniques in Cervicogenic Headache.

Gopal Nambi,[1*] Mshari Alghadier[1], Mudathir Mohamedahmed Eltayeb[2], Osama R. Aldhafian[3], Ayman K. Saleh[3,4], Nesreen Alsanousi[5], Alaa Jameel A. Albarakati[6], Mohamed A. Omar[4], Mohamed Nagah Ahmed Ibrahim[4], Abdehamid A. Attallah[4], Mohammed Abdelgwad Ismail[4], Mohamed Elfeshawy[4]

[1] Department of Health and Rehabilitation Sciences, College of Applied Medical Sciences, Prince Sattam bin Abdulaziz University, Al-Kharj, Saudi Arabia.

[2] Department of Nursing, College of Applied Medical Sciences, University, Prince Sattam bin Abdulaziz University, Al-Kharj, Saudi Arabia.

[3] Department of Surgery, College of Medicine, Prince Sattam bin Abdulaziz University, Al-Kharj, Saudi Arabia.

[4] Department of Orthopedic surgery, Faculty of Medicine for Girls, Al-Azhar University, Cairo, Egypt.

[5] Department of Biochemistry, College of Medicine, Prince Sattam bin Abdulaziz University, Al-Kharj, Saudi Arabia.

[5] Department of Surgery, College of Medicine, Umm Al-Qura University, Al-Qunfudah Branch, Makkah, Saudi Arabia.

*Corresponding Author:

E-mail: physio_gopal@rediffmail.com

DECLARATIONS

Clinical trial registration: Registered in Clinical trial registry, India (www.ctri.nic.in) with registration number: CTRI/2020/06/026092 and registered prospectively.

Consent for publication: No individual data is contained in this publication.

Availability of data and materials: The minimal data set is available as a supporting file.

Competing interest: None declared

Funding: This study is supported via funding from Prince Sattam bin Abdulaziz University project number (PSAU/2023/R/1444). There was no additional external funding received for this study."

Author Contributions: Gopal Nambi (GN), Mshari Alghadier (MA), Mudathir Mohamedahmed Eltayeb (MM), Osama R. Aldhafian (OA), Ayman K. Saleh (AS), Nesreen Alsanousi (NA), Alaa Jameel A. Albarakati (AA), Mohamed A. Omar (MO), Mohamed Nagah Ahmed Ibrahim (MI), Abdehamid A. Attalah (AA), Mohammed Abdelgwad Ismail (MI) Mohamed Elfeshawy (ME).

Conceptualization: GN, MA, MM, OA, AS, NA, AA, MO, MI, AA, MI, ME. Data curation: MM, OA, AS, NA, AA, MO, MI, ME. Formal analysis: GN, MA, AS, NA, AA, MO, MI, MI, ME. Funding acquisition: GN, MA, MM, OA, MO, MI, AA, MI, ME. Investigation: GN, MA, MM, OA, AS, NA, AA, MO. Methodology: GN, MA, MM, OA, MO, MI, AA, MI. Project administration: MA, MM, AS, NA, AA, AA, MI, ME. Resources: MM, OA, AS, NA, AA, AA, MI, ME. Software: OA, AS, NA, AA, AA, MI, ME. Validation: MM, OA, AS, MO, MI, AA, MI, ME. Visualization: GN, MM, OA, AS, MO, MI, MI, ME. Writing—original draft: MM, OA, AS, NA, MI, AA, MI. Writing—review and editing: GN, MA, MM, OA, AS, NA, AA, MO, MI, AA, MI, ME.

Acknowledgments: This study is supported via funding from Deanship of scientific research, Prince Sattam bin Abdulaziz University, Al Kharj, Saudi Arabia.

Abstract

Background: There is ample evidence supporting the use of different manipulative therapy techniques for Cervicogenic Headache (CgH). However, no technique can be singled as the best available treatment for patients with CgH. Therefore, the objective of the study is to find and compare the clinical effects of cervical spine over thoracic spine manipulation and conventional physiotherapy in patients with CgH.

Design, Setting, Participants: It is a prospective, randomized controlled study conducted between July 2020 and January 2023 at the University hospital. N=96 eligible patients with CgH were selected based on selection criteria and they were divided into cervical spine manipulation (CSM; n=32), thoracic spine manipulation (TSM; n=32) and conventional physiotherapy (CPT; n=32) groups, and they received the respective treatment for four weeks. Primary (CgH frequency) and secondary (CgH pain intensity, CgH disability, neck pain frequency, neck pain intensity, neck pain threshold, flexion rotation test (right and left), neck disability index and quality of life) scores were measured. The effects of treatment at various intervals were analyzed using a 3 × 4 linear mixed model analysis (LMM), with treatment group (cervical spine manipulation, thoracic spine manipulation, and conventional physiotherapy) and time intervals (baseline, 4 weeks, 8 weeks, and 6 months), and the statistical significance level was set at $P < 0.05$.

Results: The baseline demographic and clinical variables did not show any significance difference (p>0.05). The reports of the CSM, TSM and CPT group were compared between the groups. Four weeks following treatment CSM group showed more significant changes in primary (CgH frequency) and secondary (CgH pain intensity, CgH disability, neck pain frequency, pain intensity, pain threshold, flexion rotation test (Right and left), neck disability index and quality of life) than TSM and CPT groups (p=0.001). The same gradual improvement was seen in CSM group when compared to TSM and CPT groups (p=0.001) in the above variables at 8 weeks and at 6 months follow up.

Conclusion: The reports of the current randomized clinical study found that CSM resulted in significantly better improvements in pain parameters (intensity, frequency and threshold) functional disability and quality of life in patients with CgH than thoracic spine manipulation and conventional physiotherapy.

Keywords: Cervicogenic headache; cervical spine; thoracic spine; manipulation; physiotherapy

Clinical trial registration: CTRI/2020/06/026092 trial registered prospectively on 24/06/2020.

Introduction

Globally, headache disorders affect approximately 66% of the population between the ages of 18 and 65 years at least once a year. Sixty-six percentage of men and fifty-seven percentage of women report headache at least once in their lifetime which reduces the quality of life, work productivity and increased costs to society.[1] Cervicogenic headache (CgH) is a distinct form of headache and accounts for 17.8% of all headaches, the prevalence rate is between 15 and 20% among cases of chronic headache.[2] The prevalence rate of CgH is 0.21% in females and 0.13% in males and has various causative factors.[3] It has a significant negative socioeconomic impact and is a burden on the community and public health.[4] The causative factor for the headache is located in the neck region and the pain is made worse by movements of the head and neck.[2] The most accepted mechanism of CgH is found between the trigeminal nerve and C1 – C3 nerves in the trigemino-cervical nucleus.[5] It usually arises from musculoskeletal structures such as the cervical vertebra, intervertebral disc, or paravertebral muscles. The clinical features of CgH include unilateral or bilateral headache, limited range of motion (ROM) of the neck, and radiating pain to the head or face region. [6]

Generally, CgH is diagnosed on the basis of a detailed history and clinical assessment. [7] Physical examinations typically reveal pain in the cervical region – neck pain (NP), decreased neck movements, upper quarter muscle tightness and loss of muscle properties such as excitability, contractility, extensibility and elasticity.[8] The cervical flexion rotation test (CFRT) is a valid, reliable and accurate method with an overall diagnostic accuracy of 91% for assessing cervical range of motion in patients with CgH.[9] The management of CgH consists of pharmacological and non-pharmacological methods, in which the pharmacological means are associated with many side effects such as damage to liver or kidneys, diarrhea, constipation and allergic reactions.[10] There are also many non-pharmacological treatment modalities available such as; physical modalities, positional release therapy (PRT), muscle strengthening

exercises, ergonomic guidance and patient education etc.[11] It has been estimated that 34% of US citizens receive some sort of physiotherapy for CgH each year.[12]

In physiotherapy, joint mobilization and manipulation are the most commonly used treatment modality for treating patients with CgH. [13] The manipulation technique commonly used to treat CgH tarrget two different regions in the spine such as cervical and thoracic spine. Cervical spine manipulation (CSM) and thoracic spine manipulation (TSM) technique uses high velocity, low amplitude thrusts (HVLAT) maneuver. Some studies have looked solely at the effects of manipulating the cervical spine in cases of Cervicogenic headache. [6,14-15] Dunning JR et al investigated that six to eight sessions of upper cervical and upper thoracic manipulation were shown to be more effective than mobilization and exercise in patients with CgH, and the effects were maintained at 3 months.[16] Haas et al. investigated the effectiveness of cervical manipulation in patients with CgH.[17] Similarly, McDevitt AW et al. found that thoracic spine manipulation alone significantly improved neck-related disability in CgH, but had no effect on headache-related disability but participants reported overall improvement of their condition.[18] However, so far no studies have compared and investigated the individual effects of cervical spine manipulation, thoracic spine manipulation or conventional physiotherapy in treating patients with CgH.

Numerous studies have supported the application of various manipulative therapy approaches for the treatment of CgH. [13-18] Nevertheless, there is a scarcity of evidence in comparing the individual effects of cervical and thoracic manipulation approaches in Cervicogenic headache, particularly regarding its clinical and functional aspects. Additionally, no studies have attempted to address the shortcomings and gaps observed in the existing literature on the management of CgH, such as a lack of comparison between manipulation in two different regions, poor study designs, quality and small sample sizes. Therefore, our study objective was to compare and investigate the individual effects of cervical and thoracic manipulation techniques on patients with Cervicogenic headache. This randomized clinical trial hypothesized that there is a difference in primary and secondary outcome measures between cervical spine manipulation, thoracic spine manipulation, and conventional physiotherapy for treating of patients with CgH.

Materials and methods

Study design

The trial was a parallel-group, prospective, randomized controlled trial. The required participants were screened and diagnosed by a physician at the University hospital between 1st July 2020 and 31st July 2022 in accordance to the Cervicogenic headache diagnostic criteria 11.2.1 from the ICHD-3 (International Classification of Headache Disorders) [19] and the disease CgH falls under the International classification of disease -10 (ICD-10) code of G44. 841.[7] Ninety-six (N=96) participants who fulfilled the eligibility criteria were randomly allocated into three groups equally: the cervical spine manipulation (CSM; n=32), thoracic spine manipulation (TSM; n=32), and conventional physiotherapy (CPT; n=32) groups through a computer-generated simple random table and the allocation of the participants to each group was concealed using sealed envelopes. The computer did not generate the group until it was time to randomize each participant, ensuring that the allocation was concealed. No significant changes were made while the study was being carried out because it was designed as a follow-up to a pilot study and the 6-month follow-up data collection was completed on 31st January 2023.

The research was conducted at #####, and the Department Ethical Committee (DEC) granted ethical approval under the reference number RHPT/019/042. The DEC accepted the study protocol as well as the informed consent forms. The study involved human participants and followed the instructions outlined in the Declaration of Helsinki (1964) and was prospectively registered in clinical trial.gov.in CTRI/2020/06/026092 on June 24, 2020.

Participants

Participants aged between 18-60 years and suffering from unilateral or bilateral CgH (>3 months) were allowed to participate in the study. Patients with pain intensity ≥3 on a visual analog scale (VAS), CgH resulting from pain in the neck followed by headache, limited neck movements, neck stiffness and cervical spine disorders were allowed to participate in the study. Other primary headaches such as migraine and tension-type headaches (TTH), whiplash injuries, participants who show signs of the five ‘D’s’ (dizziness, drop attacks, dysarthria, dysphagia, diplopia) or who have signs of the three ‘N’s (nystagmus, nausea, other neurological symptoms (cord compression or nerve root involvement), contraindications to manipulative therapy (tumour, degenerative and inflammatory arthritis, osteoporosis, dislocation, fractures, and steroid intake), underwent previous head and neck surgeries, had physiotherapy or other

complementary therapies in the last three months were excluded. The flow of the study program was documented by following the CONSORT guidelines and it was displayed in (Fig 1). [20]

Fig 1. Flow chart showing the study details.

Interventions

Certified physiotherapists having 10 – 15 years of experience in providing spinal manipulation for patients with CgH provided the treatment to both groups. All the participants in the three groups had given their informed consent to participate in the study after understanding the detailed information about the study protocol. All the participants in the three groups received 10-minutes of hydro collator pack application to relax the muscles of the area around the neck and upper back. Following this, the participant's neck muscles and joints were assessed for any musculoskeletal dysfunction. After that, the participants were given the manipulation techniques as per the directions provided in the study protocol. Standardized treatment techniques were used for all the group participants to reduce intervention bias. The procedures of intervention and follow-up measurements were recorded in standardized forms. During the study period, the participants were asked to refrain from taking any other type of intervention, they received the concerned interventions 3 times per week for 4 weeks.

Spinal manipulation therapy

Peterson and Bergman defined spinal manipulation therapy (SMT) is a high-velocity low-amplitude thrust (HVLAT) technique. [21] Four experienced physiotherapists having experience in SMT performed this technique after evaluation of each participant by physical examination and palpation. Then the therapist manipulated the upper cervical (C1-2) spine for the CSM group and upper thoracic (T1-T2) spine for the TSM group by following the study protocol irrespective of joint dysfunction. For both the upper cervical (C1-2) and upper thoracic (T1-T2) manipulations, if no popping or cracking sound was heard on the first attempt, the therapist repositioned the patient and performed a second manipulation. A maximum of 2 attempts were performed on each patient. The clinicians were instructed that the manipulations are likely to be accompanied by multiple audible popping sounds. [16] If any participant reported any new red flag signs or showed no signs for manipulation, such as no pain or musculoskeletal dysfunction, then the procedure was not performed.

Cervical spine manipulation (CSM)

The patient was asked to lie comfortably in a supine position, in which the manipulation targeted the C1–C2 vertebra. The patient's head was kept in a "cradle hold" method" where the head was free from the treatment table. The left posterior arch of the C1 vertebra (atlas) was held with the lateral aspect of the proximal phalanx of the therapist's index finger of the left hand. The right hand of the therapist holds the chin of the patient. To localize the forces to the left C1-C2 vertebral articulation, the patient was positioned using extension, a posterior-anterior (PA) shift, ipsilateral side-bend and contralateral side-shift. While maintaining this position, the therapist performed a single high-velocity, low-amplitude thrust (HVLAT) manipulation to the left atlanto-axial joint using right rotation in an arc toward the underside eye and translation toward the table. This was repeated using the same procedure but directed to the right C1-C2 articulation. The selection of the spinal segments to target was prescriptive (C1–C2) and it was based on the study protocol (Fig 2). The manipulation was done first on the pain-free side and then on the painful side and the rotation range was limited by the target vertebra. [6]

Fig 2. Figure showing the cervical spine manipulation.

Thoracic spine manipulation (TSM)

The patient was asked to lie comfortably in a supine position and the manipulation targeted the T1–T2 vertebra. For this technique, the patient held her/his arms and forearms across the chest with the elbows aligned in a supero inferior direction. The therapist contacted the transverse processes of the lower vertebrae of the target motion segment with the thenar eminence and middle phalanx of the third digit. The upper lever was localized to the target motion segment by adding rotation away and side-bend towards the therapist while the underside hand used pronation and radial deviation to achieve rotation toward and side-bend away moments, respectively. The space inferior to the xiphoid process and costo-chondral margin of the therapist was used as the contact point against the patient's elbows to deliver a manipulation in an anterior to posterior direction targeting T1-2 bilaterally (Fig 3). [18]

Fig 3. Figure showing the thoracic spine manipulation.

Conventional physiotherapy (CPT)

The participants of the CPT group received massage therapy for 15 minutes using Queen Helene, Cocoa Butter face & body cream, New York, USA. The participant was asked

to lie down in a prone position, with the anterior aspect of the head is resting on a face hole in the couch. The treating therapist stands by the patient's head side and uses the tips of the middle fingers of both hands to perform circular kneading on both sides of the C1 to C7 vertebra. This maneuver was repeated 3 times for each cervical vertebra, beginning from the C7 vertebra and working towards the C1 vertebra. Then the head was turned to right side, and the circular kneading was performed on the sub-occipital and paravertebral muscles and the same procedure was done in the left side of the neck. [22]

Participants in all the three groups received ten minutes of hydro collator heat prior to intervention (manipulation or massage). Also they were asked to perform neck isometric exercises three times a day, every day for 4 weeks. The patient was asked to keep his hand over his forehead and resist the forward movement of his neck for 10 seconds and the same movement was repeated 15 times. Similarly, the patient was asked to keep the hand on the posterior and lateral sides of the head, and resist the backward and sideways movements of the neck (Fig 4). Also, static active stretching exercises for the upper trapezius, levator scapulae, scalene, and sternocleidomastoid muscles were taught to the patients, which was maintained for the 30s with 3 repetitions. [23] The patients were instructed to keep doing this set of exercises after 4 weeks of various intervention protocols and they were asked to maintain an exercise log book to check the treatment compliance.

Fig 4. Figure showing the neck isometric exercises.

Outcomes

All the outcome measures were recorded by a blinded physiotherapist, and the scores were entered in a data sheet. The scores were measured at the beginning of the study, after 4-weeks, 8-weeks, and at 6-months.

Primary outcome

CgH frequency: It is a self-administered outcome variable where the patient enters his CgH pain experience in a medical log book every evening to find the number of painful days in 4-weeks. [24]

Secondary outcome

CgH pain intensity: The pain intensity of CgH was assessed using visual analogue scale (VAS). Patients rated their typical level of pain status during the previous week on a 10 cm

horizontal line, with one end 0 representing "no pain" and the other end 10 representing "worst pain imaginable." [25]

CgH disability: The Headache Impact Test (HIT) questionnaire is a valid and reliable tool to assess the level of disability in CgH patients. It consists of six items: pain, social functioning, role functioning, vitality, cognitive functioning, and psychological distress. Each item is rated using 5 response categories (never, rarely, sometimes, very often, or always), each category of which is associated with a numerical value (6, 8, 10, 11, and 13, respectively), resulting in a range of possible total summed scores of 36-78. The score categories are no or mild disability (49 or less), moderate disability (50–55), severe disability (56–59), and complete disability (60–78). [26]

NP frequency: It is a self-administered outcome variable where the patient enters his neck pain experience in a medical log book every evening to find the number of painful days in 4-weeks. [24]

NP intensity: The pain intensity of neck pain was assessed using visual analogue scale (VAS). Patients rated their average pain intensity over the past week on a 10 cm horizontal line, with one end 0 representing "no pain" and the other end 10 representing "worst pain imaginable." [25]

NP pressure threshold: It is the lowest intensity at which a given stimulus is perceived as painful and it was measured using an instrument called an Algometer (Baseline, 22-pound dolorimeter, ID, USA). The tip was placed over the fixed points (trigger point on the upper trapezius muscle) on the neck region which is identified through palpation techniques. It is a reliable and valid tool for determining pain threshold. [27]

Cervical flexion–rotation test (FRT): The cervical flexion–rotation test is done with the patient in a supine lying position. The therapist passively maintains the patient's neck into full flexion to relax the structures of the middle and lower cervical spine, then the patient's head is passively rotated in each direction while the flexed position is maintained and the range of motion is measured. [9]

Neck disability index (NDI): It is a reliable and valid self-reported questionnaire with ten items scored on a 0 to 5 scale. The grades of disability are determined based on the scores obtained,

which are as follows: 10-29% mild; 30-49% moderate; 50-69% severe; 70% or more is a complete disability. [28]

Quality of life: The EQ-5D (Euro Qol 5D) is self-administered health related quality of life (HRQOL) questionnaire, which measures the five dimensions of quality of life. It includes mobility, self-care, usual activities, pain/discomfort, and anxiety/depression. It is used to assess the CgH patients' overall quality of life with a scale of 0 (worst) to 100 (best). [29]

Sample size

For calculating the number of subjects to be include in the study, the primary outcome measure CgH frequency in days was taken into consideration based on previous pilot study which found the effect of spinal manipulation in the treatment of CgH, with 10 subjects in each group. Using the G-Power software (version 3.1.9.2; Franz Faul, University of Kiel, Germany), assuming a two-sided $\alpha = 0.05$, and power ($1-\beta = 0.80$), to detect an effect size of 1.2 CgH days and mean difference of 4 CgH days (between groups) and a standard deviation of 0.5, an approximately 28 samples were required. In assuming 10% dropout, we enrolled 32 subjects in each group.

Blinding

Because of the experimental nature of the study methodology, it was not feasible to blind the treating therapist as well as the participants of the study. The therapists who assessed the outcome variables at baseline, 4-weeks, 8-weeks, and 6-months were blinded. Therefore, the therapist providing the treatment and the therapist measuring the data were different individuals. In addition, the outcome-measuring therapist continued to be masked to the participant's groups at all-time intervals. Nevertheless, for the primary outcome measurement, the assessor was the participant, so the study cannot properly be called assessor-blind either. Also, participants were asked not to discuss their treatment details with their peers or the outcome-measuring therapist. In addition, authors did not have access to information that could identify individual participants during or after data collection.

Statistical methods

The normality of study participants' demographic characteristics was analyzed through the Kolmogorov-Smirnov test. The outcome data were presented in the form of a mean and standard deviation with a 95% confidence interval. The study followed the intention to treat principle method by including the participants' missing follow-up data in the data analysis. The effects of treatment at different time intervals were analyzed using a 3 × 4 linear mixed model analysis (LMM), with patient as a random factor and treatment groups; cervical spine manipulation, thoracic spine manipulation, and conventional physical therapy and time intervals; baseline, four weeks, eight weeks, and at six months) as fixed factors. Following the omnibus 3x4 LMM model analysis, post hoc Bonferroni analysis was done to compare the three study groups pair-wise. For all the statistical tests a statistical significance level of $\alpha=0.05$ was set and the software GraphPad-Prism (version 9.1), Boston, MA, USA was used for analysis.

Results

Participants

Out of the 130 participants screened, eight had a VAS score greater than 8, ten participants were having some sort of orthopaedic injuries, four participants were undergone joint surgeries, and twelve refused to involve in the research and were excluded. N=96 participants were chosen based on the eligibility criteria and allocated to one of the three groups. Two participants in the CSM and CPT groups, and three in the TSM group, did not complete the 4-week treatment program with a 6-month follow-up (Fig 1). Compliance with follow-up data collection at 6 months was 93%, adherence to study protocols (e.g., number of visits) was 100%, and none of the participants in the three groups received any additional care that was not included in the three study interventions. At baseline, the demographic characters such as age (years), height (cm), weight (kg), and BMI (kg/m^2) scores did not report statistically significant variation between the groups ($p>0.05$). In all three groups, females (53-56%) are affected more than males. At baseline, the clinical variables also did not show any significant difference between groups (p0.05). The clinical presentation of headache is more unilateral (78% - 84%) than bilateral, and the majority of CGH cases have associated neck pain (84% - 88%). (Table 1). Due to random sample design the three groups were uniform in baseline characteristics and the baseline outcome variables.

Primary outcome

The mean and standard deviation (SD) of the CgH frequency score between the three groups, at four-time periods, was shown in Table 2 and 3. The 3 × 4 linear mixed model (LMM) analysis reported statistically significant change in CgH frequency ($p=0.012$). Over 4 weeks of different interventions, there is a significant change in CgH pain frequency level between the CSM (7.9; CI 95% 7.41 to 8.38), and TSM (4.7 CI 95% 4.21 to 5.18), groups ($p=0.001$). A similar improvement can be seen in 8-weeks and at 6-months' measurement. The post-hoc Bonferroni analysis and the standard mean difference showed more percentage of improvement in CgH pain frequency in the CSM group than TSM and CPT groups (Fig 5a). The complete interpretation shows a slight leaning towards the CSM group than TSM and CPT group in CgH frequency.

Fig 5a. Pre and post outcome measures of CSM, TSM and CPT groups.

Secondary outcomes

The mean and standard deviation (SD) of the secondary outcomes between the three groups, at four-time periods, was shown in Table 2 and 3. The 3 × 4 linear mixed model showed statistically significant variation in CgH pain intensity ($p = 0.022$), CgH disability ($p = 0.043$), neck pain frequency ($p = 0.007$), neck pain intensity ($p = 0.002$), neck pain threshold ($p = 0.015$), flexion rotation test (Right and left) ($p = 0.008, 0.006$), neck disability index ($p = 0.003$) and quality of life ($p = 0.004$).

After 4 weeks of intervention, there are statistically significant variations in CgH pain intensity, CgH disability, NP (frequency, intensity, and threshold), FRT (Right and left), NDI, and QoL score between the CSM (2.9; CI 95% 2.71 to 3.08), (17.1; CI 95% 14.7 to 19.6) (12.7; CI 95% 12.08 to 13.31), (3.1; CI 95% 2.89 to 3.30), (-18.7; CI 95% -29.18 to -8.21), (-12.9, -12.31; CI 95% -16.12 to -9.67, -15.30 to -9.31), (23.64; CI 95% 20.55 to 26.72), (-32.5; CI 95% -35.89 to -29.10) and TSM (2.0; CI 95% 1.81 to 2.18), (8.8; CI 95% 6.3 to 11.2), (7.9; CI 95% 7.28 to 8.51), (1.8; CI 95% 1.59 to 2.00), (-8.8; CI 95% -19.28 to 1.68), (-5.91, -4.31; CI 95% -9.13 to -2.68, -7.30 to -1.31), (11.36; CI 95% 8.27 to 14.44), (-15.7; CI 95% -19.09 to -12.30) groups ($p<0.001$) respectively. The post hoc Bonferroni analysis and the standard mean difference showed more percentage of improvement in the secondary outcomes in the CSM

group than TSM and CPT groups (Fig 5a and 5b). The complete interpretation shows a slight leaning towards the CSM group than TSM and CPT group in all the secondary variables.

Fig 5b. Pre and post outcome measures of CSM, TSM and CPT groups.

Discussion

This is the first powered randomized clinical study to find the individual and comparative effects of cervical and thoracic manipulation for patients with CgH. The reports of the study show that cervical manipulation was superior to thoracic manipulation and conventional PT (massage) in treating patients with CgH. According to this study, after four weeks of intervention the cervical spine manipulation (CSM) group showed statistically significant changes in all the outcome measures in CgH patients. When compared to TSM and conventional physical therapy, CSM is more effective in reducing the primary outcome CgH frequency, which was clinically important. The same improvements were noted in all the other outcome measures such as CgH pain, neck pain frequency, neck pain intensity, neck pain threshold, range of motion, and quality of life and these reports were supported by Dunning JR et al study. [16] So far, no studies have looked at the changes in CgH disability and neck disability, but this study looked at the benefits of cervical spine manipulation over thoracic spine manipulation in CgH patients. However, these findings were contradictory to the observation of Borusiak et al. [30]

According to our findings, HVLAT technique in the cervical vertebra may promote the afferent nerve fiber activity via joint receptors. It improves the overall action and properties of the neck muscles by activating the alpha motor neuron. [31] It alters the sensory fiber activity by activating the joint receptors, thereby changing the α-motor neuron activity levels and subsequent muscle reaction. Because of the high mobility of the cervical spine, CSM can stimulate the receptors of deep neck muscles and sub-occipital muscles, which TSM is not able to do. [32] Other theories for the pain-modulating effects of cervical manipulation included biomechanical, vertebral (temporal summation), and neural (central descending pain inhibitory pathway) mechanisms which were noted by Bialosky JE et al and Haavik-Taylor H et al. [6,33-34]

Our findings also showed that thoracic spine manipulation improved both primary and secondary outcomes in CgH patients. TSM was also helpful in improving the pain parameters, functional disability and quality of life significantly. It was supported by McDevitt AW et al

[18] and suggests that TSM had no effect on headache-related disability but resulted in significant improvements in neck-related disability and participant reported perceived improvement. Although several types of research have been conducted to find the effects of different types of spinal manipulation, the real mechanical and neuro-physiological alterations behind these changes have not yet been found and are unclear, but Bialosky et al. suggest that the effects may be due to potential neurophysiological and biomechanical effects, as well as possibly placebo effect. [33] According to Suvarnnato T et al, the neurophysiologic response of pain reduction in patients with chronic neck pain through TSM is that it induces a reflex inhibition of pain or muscle relaxation reflex by modifying the discharge of proprioceptive Group I and II afferents. It also activates descending inhibitory mechanisms resulting in pain reduction in distant areas from the manipulation. Through these mechanisms, the thoracic manipulation may induce ventral periaqueductal gray (vPAG) in the brain, which activates endogenous opioid peptides resulting in pain reduction in different areas. [35] The little changes in the conventional physical therapy group on pain intensity and other outcome variables explained the analgesic effect of CPT on cervicogenic headache. Application of massage on the trigeminal-cervical area reduces inflammatory responses, reduces neural sensitivity and plays a significant function in decreasing the tension of the sub-occipital and para-vertebral cervical spine muscles, which is another important mechanism of physical therapy on CgH patients. [36-37] The findings of this trial would assist physiotherapists in making decisions to select the best manual therapy approach for CgH patients.

Limitations

The study had some limitations during its execution, which should be considered for future studies. First, the study included both genders, but the reports collected were not calculated independently during the statistical analysis, these gender differences may influence the research reports. Second, it is impossible to ensure that the subjects completed the questionnaires daily rather than after a week or four weeks. The standard for headache outcomes is a headache diary that uses technology to ensure that frequency and pain intensity are reported immediately on the day stipulated, not added in later. Third, this study lacks a placebo group to determine the true effects of treatment groups. The beneficial effects of various manipulation techniques on pain and other symptoms in Cervicogenic headache were investigated. Fourth, the differences in the length of study visits between groups adds attention bias and could reduce differences between manipulation groups and the massage group.

Finally, the treatment preference of physiotherapists and patients was not asked which could have affected the results due to clinical equipoise.

Conclusion

The reports of the current randomized clinical study found that cervical spine manipulation resulted in significantly better improvements in pain parameters (intensity, frequency and threshold), functional disability and quality of life in patients with cervicogenic headache than thoracic spine manipulation and conventional physiotherapy. The report also provided an additional evidence in the field of manual therapy for patients with cervicogenic headache. Future studies are recommended to find the biomechanical and biochemical reactions behind the clinical and functional changes of these manipulation techniques in treating patients with CgH symptoms.

List of abbreviations: CgH - Cervicogenic Headache, ROM - range of motion, FRT - flexion rotation test, ICHD - International Classification of Headache Disorders, CSM - cervical spine manipulation, TSM - thoracic spine manipulation, CMT - conventional massage therapy, HVLAT - high-velocity low amplitude thrust, NDI - Neck disability index, QOL - Quality of life

References

1. AlQarni MA, Fayi KA, Al-Sharif MN, Siddiqui AF, Alhazzani AA. Prevalence of migraine and non-migraine headache and its relation with other diseases in the adults of Aseer Region, Saudi Arabia. *J Family Med Prim Care*. 2020;9(3):1567-1572.
2. Verma S, Tripathi M, Chandra PS. Cervicogenic Headache: Current Perspectives. *Neurol India*. 2021;69(Supplement):S194-S198.
3. Chaibi A, Russell MB. Manual therapies for cervicogenic headache: a systematic review. *J Headache Pain*. 2012;13(5):351-359.
4. Stovner L, Hagen K, Jensen R, et al. The global burden of headache: a documentation of headache prevalence and disability worldwide. *Cephalalgia*. 2007;27(3):193–210.
5. Pourahmadi M, Mohseni-Bandpei MA, Keshtkar A, et al. Effectiveness of dry needling for improving pain and disability in adults with tension-type, cervicogenic, or migraine headaches: protocol for a systematic review. *Chiropr Man Therap*. 2019;27:43.

6. Lerner-Lentz A, O'Halloran B, Donaldson M, Cleland JA. Pragmatic application of manipulation versus mobilization to the upper segments of the cervical spine plus exercise for treatment of cervicogenic headache: a randomized clinical trial. *J Man Manip Ther*. 2021;29(5):267-275.
7. Sjaastad O, Fredriksen TA, Pfaffenrath V. Cervicogenic headache: diagnostic criteria. The Cervicogenic Headache International Study Group. Headache. 1998;38(6):442–5.
8. Hall T, Briffa K, Hopper D. Clinical evaluation of cervicogenic headache: a clinical perspective. *J Man Manip Ther*. 2008;16(2):73-80.
9. Hall TM, Briffa K, Hopper D, Robinson K. Comparative analysis and diagnostic accuracy of the cervical flexion-rotation test. *J Headache Pain*. 2010;11(5):391-397.
10. Bogduk N.Govind J., Cervicogenic headache: an assessment of the evidence on clinical diagnosis, invasive tests, and treatment. Lancet Neurol, 2009. 8(10): p. 959–68.
11. Page P. Cervicogenic headaches: an evidence-led approach to clinical management. *Int J Sports Phys Ther*. 2011;6(3):254-266.
12. Clarke TCBL, Stussman BJ, Barnes PM, Nahin RL. Trends in the use of complementary health approaches among adults: United States, 2002–2012. Natl Health Stat Rep. 2015;79:1–16.
13. Fernández-de-Las-Peñas C, Courtney CA. Clinical reasoning for manual therapy management of tension type and cervicogenic headache. *J Man Manip Ther*. 2014;22(1):44-50.
14. Haas M, Bronfort G, Evans R, Schulz C, Vavrek D, Takaki L, Hanson L, Leininger B, Neradilek MB. Dose-response and efficacy of spinal manipulation for care of cervicogenic headache: a dual-center randomized controlled trial. Spine J. 2018 Oct;18(10):1741-1754.
15. Nilsson N, Christensen HW, Hartvigsen J. The effect of spinal manipulation in the treatment of cervicogenic headache. *J Manipulative Physiol Ther*. 1997;20(5):326-330.
16. Dunning JR, Butts R, Mourad F, Young I, Peñas CF, Hagins M et al. Upper cervical and upper thoracic manipulation versus mobilizationand exercise in patients withcervicogenic headache: a multi-center randomized clinical trial. BMC Musculoskeletal Disorders. 2016;17:64.
17. Haas M, Spegman A, Peterson D, Aickin M, Vavrek D. Dose response and efficacy of spinal manipulation for chronic cervicogenic headache: a pilot randomized controlled trial. Spine J. 2010;10(2):117–28

18. McDevitt AW, Cleland JA, Rhon DI, et al. Thoracic spine thrust manipulation for individuals with cervicogenic headache: a crossover randomized clinical trial. *J Man Manip Ther*. 2022;30(2):78-95.
19. The International Classification of Headache Disorders, 3rd edition. Cephalalgia. 2018, 38(1):1–211.
20. Butcher NJ, Monsour A, Mew EJ, et al. Guidelines for Reporting Outcomes in Trial Reports: The CONSORT-Outcomes 2022 Extension. *JAMA*. 2022;328(22):2252-2264.
21. Peterson DH, Bergmann T. Chiropractic technique: principles and procedures. 3rd ed. St. Louis: Mosby; Chapter 5, 2010, 246-275.
22. Diana Hopper, Yogita Bajaj, Chor Kei Choi, et al. A pilot study to investigate the short-term effects of specific soft tissue massage on upper cervical movement impairment in patients with cervicogenic headache. Journal of Manual and Manipulative Therapy 2013: 21(1); 18 – 24.
23. Nambi G, Alghadier M, Ebrahim EE, et al. Comparative Effects of Mulligan's Mobilization, Spinal Manipulation, and Conventional Massage Therapy in Cervicogenic Headache-A Prospective, Randomized, Controlled Trial. *Healthcare (Basel)*. 2022;11(1):107.
24. Bendtsen L, Bigal ME, Cerbo R, Diener HC, Holroyd K, Lampl C, Mitsikostas DD, Steiner TJ, Tfelt-Hansen P. International Headache Society Clinical Trials S: Guidelines for controlled trials of drugs in tension-type headache: second edition. Cephalalgia. 2010;30(1):1–16.
25. Jensen MP, Karoly P, Braver S. The measurement of clinical pain intensity: a comparison of six methods. Pain. 1986;27(1):117–26.
26. Kosinski M, Bayliss MS, Bjorner JB, Ware Jr JE, Garber WH, Batenhorst A, Cady R, Dahlof CG, Dowson A, Tepper S. A six-item short-form survey for measuring headache impact: the HIT-6. Qual Life Res Int J Qual Life Asp Treat Care Rehab. 2003;12(8):963–74.
27. Chesterton LS, Sim J, Wright CC, Foster NE. Interrater reliability of algometry in measuring pressure pain thresholds in healthy humans, using multiple raters. Clin J Pain. 2007;23(9):760–6.
28. Vernon H, Mior S. The neck disability index: a study of reliability and validity. J Manipulative Physiol Ther. 1991;14:7. 409–415.
29. Luo N, Johnson JA, Shaw JW, Feeny D, Coons SJ. Self-reported health status of the general adult U.S. population as assessed by the EQ-5D and Health Utilities Index. Med Care. 2005;43(11):1078–86.
30. Borusiak P, Biedermann H, Bosserhoff S, Opp J. Lack of efficacy of manual therapy in children and adolescents with suspected cervicogenic headache: results of a prospective, randomized, placebo-controlled, and blinded trial. *Headache*. 2010;50(2):224-230.

31. Bolton PS, Budgell BS. Spinal manipulation and spinal mobilization influence different axial sensory beds. Med Hypotheses. 2006;66(2):258–62.

32. Cassidy JD, Lopes AA, Yong-Hing K. The immediate effect of manipulation versus mobilization on pain and range of motion in the cervical spine: a randomized controlled trial. J Manipulative Physiol Ther. 1992;15(9):570–5.

33. Bialosky JE, Bishop MD, Price DD, Robinson ME, George SZ. The mechanisms of manual therapy in the treatment of musculoskeletal pain: a comprehensive model. Man Ther. 2009;14(5):531–8.

34. Haavik-Taylor H, Murphy B. Cervical spine manipulation alters sensorimotor integration: a somatosensory evoked potential study. Clin Neurophysiol. 2007;118(2):391–402. doi:10.1016/j.clinph.2006.09.014

35. Suvarnnato T, Puntumetakul R, Kaber D, Boucaut R, Boonphakob Y, Arayawichanon P, Chatchawan U. The effects of thoracic manipulation versus mobilization for chronic neck pain: a randomized controlled trial pilot study. J Phys Ther Sci. 2013 Jul;25(7):865-71.

36. Panneton WM, Gan Q, Livergood RS. A trigeminoreticular pathway: implications in pain. PloS one. 2011; 6: e24499.

37. Youssef EF, Shanb AS. Mobilization versus massage therapy in the treatment of cervicogenic headache: a clinical study. *J Back Musculoskelet Rehabil*. 2013;26(1):17-24.

Table 1: Demographic details of CSM, TSM and CPT groups.

Sr. No	Variable		CSM	TSM	CPT	p-value
**1**	Age (year)	-	35.6 ± 3.8	34.8 ± 3.2	36.2 ± 3.7	0.295*
**2**	Gender	Male	14 (44%)	15 (47%)	14 (44%)	-
		Female	18 (56%)	17 (53%)	18 (56%)	-
**3**	Height (cm)	-	164.6 ± 3.8	163.5 ± 4.1	165.3 ± 4.4	0.215*
**4**	Weight (m)	-	72.92 ± 4.2	71.21 ± 4.6	73.83 ± 4.5	0.061*
**5**	BMI (Kg/m^2)	-	24.3 ± 2.23	24.5 ± 1.92	23.8 ± 2.01	0.378*
**6**	CgH duration (year)	-	6.6 ± 2.9	6.8 ± 3.1	5.9 ± 3.2	0.471*
**7**	CgH frequency (per day)	-	0.74 ± 0.15	0.72 ± 0.11	0.69 ± 0.12	0.293*
**8**	CgH intensity(0-10)	-	6.9 ± 1.4	7.2 ± 1.3	7.3 ± 1.3	0.461*
**9**	Headache	Unilateral	26 (81%)	25 (78%)	27 (84%)	-
		Bilateral	6 (19%)	7 (12%)	5 (16%)	-
**10**	Neck pain	Yes	28 (88%)	27 (84%)	28 (88%)	-
		No	4 (12%)	5 (16%)	4(12%)	-

* Non Significant, CSM – Cervical spine manipulation, TSM – Thoracic spine manipulation, CMT- Conventional physiotherapy, BMI – Body mass index, CgH – Cervicogenic Headache

Table 2: Pre and post mean and SD of outcome measures of CSM, TSM and CPT groups.

Sr.No	Variable		CSM	TSM	CPT	Group × Time p-value
**1**	**CgH Frequency (no of days per 4 weeks)**	Base line	16.8 ± 1.8	17.2 ± 1.9	17.4 ± 1.7	**0.012****
		4 weeks	11.2 ± 1.4	14.1 ± 1.6	15.8 ± 1.4	
		8 weeks	6.2 ± 0.9	9.5 ± 0.9	13.4 ± 1.1	$\eta p^2 = 0.092$
		6 months	2.9 ± 0.5	6.1 ± 0.7	10.8 ± 1.1	
**2**	**CgH Pain intensity (0 -10)**	Base line	7.2 ± 0.8	6.8 ± 0.7	7.1 ± 0.7	**0.022****
		4 weeks	4.1 ± 0.5	5.2 ± 0.5	6.2 ± 0.5	
		8 weeks	2.8 ± 0.4	4.1 ± 0.4	5.1 ± 0.4	$\eta p^2 = 0.078$
		6 months	0.8 ± 0.2	1.7 ± 0.3	3.7 ± 0.4	
**3**	**CgH Disability**	Base line	57.88 ± 6.5	57.21 ± 6.8	56.91 ± 5.9	**0.043****
		4 weeks	45.73 ± 5.5	49.38 ± 5.6	52.38 ± 5.3	
		8 weeks	36.41 ± 4.3	41.73 ± 5.1	49.67 ± 4.5	$\eta p^2 = 0.102$
		6 months	31.19 ± 3.8	39.54 ± 4.5	48.37 ± 4.1	
**4**	**Neck pain frequency (no of days per 4 weeks)**	Base line	23.9 ± 3.2	23.6 ± 3.4	22.9 ± 3.6	**0.007****
		4 weeks	15.8 ± 2.2	18.5 ± 2.4	20.4 ± 2.3	
		8 weeks	9.3 ± 1.6	12.3 ± 1.8	18.2 ± 1.5	$\eta p^2 = 0.086$
		6 months	3.4 ± 0.4	8.2 ± 0.9	16.1 ± 1.5	
**5**	**Neck pain intensity (0 -10)**	Base line	7.2 ± 0.6	7.1 ± 0.6	6.9 ± 0.5	**0.002****
		4 weeks	4.8 ± 0.5	6.2 ± 0.6	6.1 ± 0.5	
		8 weeks	2.6 ± 0.4	4.2 ± 0.5	5.1 ± 0.4	$\eta p^2 = 0.096$
		6 months	0.6 ± 0.2	1.9 ± 0.4	3.7 ± 0.4	
**6**	**Neck pain threshold**	Base line	262.1 ± 21.6	261.3 ± 22.3	262.7 ± 22.5	**0.015****
		4 weeks	268.5 ± 20.2	266.3 ± 21.8	263.5 ± 21.6	
		8 weeks	274.5 ± 18.3	272.4 ± 19.4	267.8 ± 19.6	$\eta p^2 = 0.104$
		6 months	288.2 ± 17.3	278.3 ± 18.2	269.5 ± 17.3	
**7**	**Flexion rotation test (Right side)**	Base line	25.18 ± 7.4	25.12 ± 7.3	26.01 ± 7.2	**0.008****
		4 weeks	31.76 ± 6.3	30.19 ± 6.3	27.12 ± 6.5	
		8 weeks	39.73 ± 5.6	35.16 ± 5.4	31.42 ± 6.1	$\eta p^2 = 0.091$
		6 months	45.21 ± 5.3	38.22 ± 5.1	32.31 ± 5.8	
**8**	**Flexion rotation test (Left side)**	Base line	24.93 ± 6.2	24.72 ± 6.1	24.88 ± 6.3	**0.006****
		4 weeks	33.12 ± 5.9	27.93 ± 5.9	26.63 ± 5.8	
		8 weeks	37.81 ± 5.2	32.53 ± 5.5	29.92 ± 5.1	$\eta p^2 = 0.074$
		6 months	44.23 ± 5.1	36.23 ± 5.1	31.92 ± 4.9	
**9**	**Neck Disability Index**	Base line	51.01 ± 11.1	51.25 ± 10.8	50.97 ± 11.2	**0.003****
		4 weeks	37.36 ± 9.2	43.34 ± 9.8	45.32 ± 10.2	
		8 weeks	24.83 ± 6.1	31.32 ± 6.2	38.53 ± 6.9	$\eta p^2 = 0.082$
		6 months	11.28 ± 3.4	23.56 ± 5.4	34.92 ± 6.3	
**10**	**Quality of life**	Base line	24.9 ± 4.5	25.1 ± 3.9	24.8 ± 4.2	**0.004****
		4 weeks	48.3 ± 4.8	36.3 ± 4.8	34.9 ± 4.3	
		8 weeks	64.5 ± 5.5	55.3 ± 5.1	40.1 ± 4.6	$\eta p^2 = 0.112$
		6 months	79.2 ± 6.2	62.4 ± 5.9	46.7 ± 4.9	

** Significant, CSM – Cervical spine manipulation, TSM – Thoracic spine manipulation, CPT- Conventional physiotherapy, CgH – Cervicogenic Headache

Table 3: Pre and post mean difference and confidence interval (upper limit and lower limit) scores of CSM, TSM and CPT groups.

Variable / Time		Baseline	4 weeks	8 weeks	6 months
		Mean difference (95% CI)			
**CgH Frequency**	**CSM × TSM**	0.4 (-0.67 to 1.47)	2.9 (2.02 to 3.77)	3.3 (2.72 to 3.87)	3.2 (2.71 to 3.68)
	**P – value**	0.649	0.001	0.001	0.001
	**CSM × CPT**	0.6 (-0.47 to 1.67)	4.6 (3.72 to 5.47)	7.2 (6.62 to 7.77)	7.9 (7.41 to 8.38)
	**P – value**	0.381	0.001	0.688	0.874
	**TSM × CPT**	0.2 (-0.87 to 1.27)	1.7 (0.82 to 2.57)	3.9 (3.32 to 4.47)	4.7 (4.21 to 5.18)
	**P – value**	0.897	0.001	0.001	0.004
**CgH Pain Intensity**	**CSM × TSM**	-0.4 (-0.83 to 0.03)	1.1 (0.80 to 1.39)	1.3 (1.06 to 1.53)	0.9 (0.71 to 1.08)
	**P – value**	0.080	0.001	0.001	0.001
	**CSM × CPT**	-0.1 (-0.53 to 0.33)	2.1 (1.80 to 2.39)	2.3 (2.06 to 2.53)	2.9 (2.71 to 3.08)
	**P – value**	0.849	0.001	0.003	0.874
	**TSM × CPT**	0.3 (-0.13 to 0.73)	1.0 (0.70 to 1.29)	1.0 (0.76 to 1.23)	2.0 (1.81 to 2.18)
	**P – value**	0.237	0.001	0.001	0.108
**CgH Disability**	**CSM × TSM**	-0.6 (-4.48 to 3.14)	3.6 (0.39 to 6.90)	5.3 (2.55 to 8.08)	8.3 (5.88 to 10.81)
	**P – value**	0.908	0.024	0.001	0.001
	**CSM × CPT**	-0.9 (-4.78 to 2.84)	6.6 (3.39 to 9.90)	13.2 (10.49 to 16.02)	17.1 (14.7 to 19.6)
	**P – value**	0.817	0.000	0.001	0.001
	**TSM × CPT**	-0.3 (-4.11 to 3.51)	3.00 (-0.25 to 6.25)	7.9 (5.17 to 10.70)	8.8 (6.3 to 11.2)
	**P – value**	0.980	0.774	0.001	0.001
**Neck pain frequency**	**CSM × TSM**	-0.3 (-2.32 to 1.7)	2.7 (1.32 to 4.07)	3.0 (2.02 to 3.97)	4.8 (4.18 to 5.41)
	**P – value**	0.933	0.001	0.001	0.001
	**CSM × CPT**	-1.0 (-3.02 to 1.02)	4.6 (3.22 to 5.97)	8.9 (7.92 to 8.97)	12.7 (12.08 to 13.31)
	**P – value**	0.470	0.001	0.002	0.874
	**TSM × CPT**	-0.7 (-2.72 to 1.32)	1.9 (0.52 to 3.27)	5.9 (4.92 to 6.87)	7.9 (7.28 to 8.51)
	**P – value**	0.690	0.003	0.001	0.746
**Neck pain intensity**	**CSM × TSM**	-0.1 (-0.43 to 0.23)	1.4 (1.08 to 1.71)	1.6 (1.34 to 1.85)	1.3 (1.09 to 1.50)
	**P – value**	0.762	0.001	0.001	0.001
	**CSM × CPT**	-0.3 (-0.63 to 0.03)	1.3 (0.98 to 1.61)	2.5 (2.24 to 2.75)	3.1 (2.89 to 3.30)
	**P – value**	0.093	0.001	0.001	0.873
	**TSM × CPT**	-0.2 (-0.53 to 0.13)	-0.1 (-0.41 to 0.21)	0.9 (0.64 to 1.15)	1.8 (1.59 to 2.00)
	**P – value**	0.341	0.736	0.001	0.001
**Neck pain threshold**	**CSM × TSM**	-0.8 (-13.98 to 12.38)	-2.2 (-14.83 to 10.43)	-2.1 (-13.47 to 9.27)	-9.9 (-20.38 to 0.58)
	**P – value**	0.988	0.909	0.899	0.068
	**CSM × CPT**	0.6 (-12.58 to 13.78)	-5 (-17.63 to 7.63)	-6.7 (-18.07 to 4.67)	-18.7 (-29.18 to -8.21)
	**P – value**	0.993	0.614	0.343	0.000
	**TSM × CPT**	1.4 (-11.78 to 14.58)	-2.8 (15.43 to 9.83)	-4.6 (-15.97 to 6.77)	-8.8 (-19.28 to 1.68)
	**P – value**	0.965	0.857	0.602	0.118
**Flexion rotation test (Right side)**	**CSM × TSM**	-0.06 (-4.40 to 4.28)	-1.5 (-5.36 to 2.22)	-4.5 (-7.9 to -1.1)	-6.9 (-10.21 to -3.76)
	**P – value**	0.980	0.587	0.005	0.001
	**CSM × CPT**	0.83 (-3.51 to 5.17)	-4.64 (-8.43 to -0.84)	-8.3 (-11.7 to -4.91)	-12.9 (-16.12 to -9.67)
	**P – value**	0.892	0.012	0.001	0.001
	**TSM × CPT**	0.89 (-3.45 to 5.23)	-3.07 (-6.86 to 0.72)	-3.74 (-7.13 to -0.34)	-5.91 (-9.13 to -2.68)
	**P – value**	0.877	0.136	0.027	0.001
**Flexion rotation test (Left side)**	**CSM × TSM**	-0.21 (-3.90 to 3.48)	-5.19 (-8.68 to -1.69)	-5.2 (-8.41 to -2.14),	-8.0 (-10.99 to -5.00)
	**P – value**	0.989	0.001	0.001	0.001
	**CSM × CPT**	-0.05 (-3.74 to 3.64)	-6.4 (-9.98 to -2.99)	-7.8 (-11.02 to -4.75)	-12.31 (-15.30 to -9.31)
	**P – value**	1.032	0.001	0.001	0.001
	**TSM × CPT**	0.16 (-3.53 to 3.85)	-1.3 (-4.79 to 2.19)	-2.61 (-5.74 to 0.52)	-4.31 (-7.30 to -1.31)
	**P – value**	0.994	0.650	0.122	0.002
**Neck Disability Index**	**CSM × TSM**	0.24 (-6.3 to 6.81)	5.98 (0.17 to 11.78)	6.49 (2.67 to 10.30)	12.28 (9.19 to 15.36)
	**P – value**	0.993	0.041	0.001	0.001
	**CSM × CPT**	-0.04 (-6.61 to 6.53)	7.96 (2.15 to 13.76)	13.7 (9.88 to 17.51)	23.64 (20.55 to 26.72)
	**P – value**	0.803	0.004	0.001	0.001
	**TSM × CPT**	-0.28 (-6.85 to 6.29)	1.98 (-3.82 to 7.78)	7.21 (3.39 to 11.02)	11.36 (8.27 to 14.44)
	**P – value**	0.994	0.696	0.001	0.001
**Quality of life**	**CSM × TSM**	0.20 (-2.30 to 2.70)	-12.0 (-14.76 to -9.23)	-9.2 (-12.2 to -6.1)	-16.8 (-20.19 to -13.40)
	**P – value**	0.980	0.001	0.001	0.001
	**CSM × CPT**	-0.10 (-2.60 to 2.40)	-13.4 (-16.16 to -10.63)	-24.4 (-27.4 to -21.37)	-32.5 (-35.89 to -29.10)
	**P – value**	0.996	0.001	0.001	0.002
	**TSM × CPT**	-0.30 (-2.80 to 2.20)	-1.4 (-4.16 to 1.36)	-15.20 (-18.22 to -12.17)	-15.7 (-19.09 to -12.30)
	**P – value**	0.956	0.452	0.001	0.001

CSM – Cervical spine manipulation, TSM – Thoracic spine manipulation, CPT- Conventional physiotherapy, CgH – Cervicogenic Headache

Supporting files:

1. CONSORT 2010 Check list.
2. Study trial protocol.
3. Ethical approval certificate
4. Dataset

Figure 5b Click here to access/download;Figure;Figure 5b.tiff

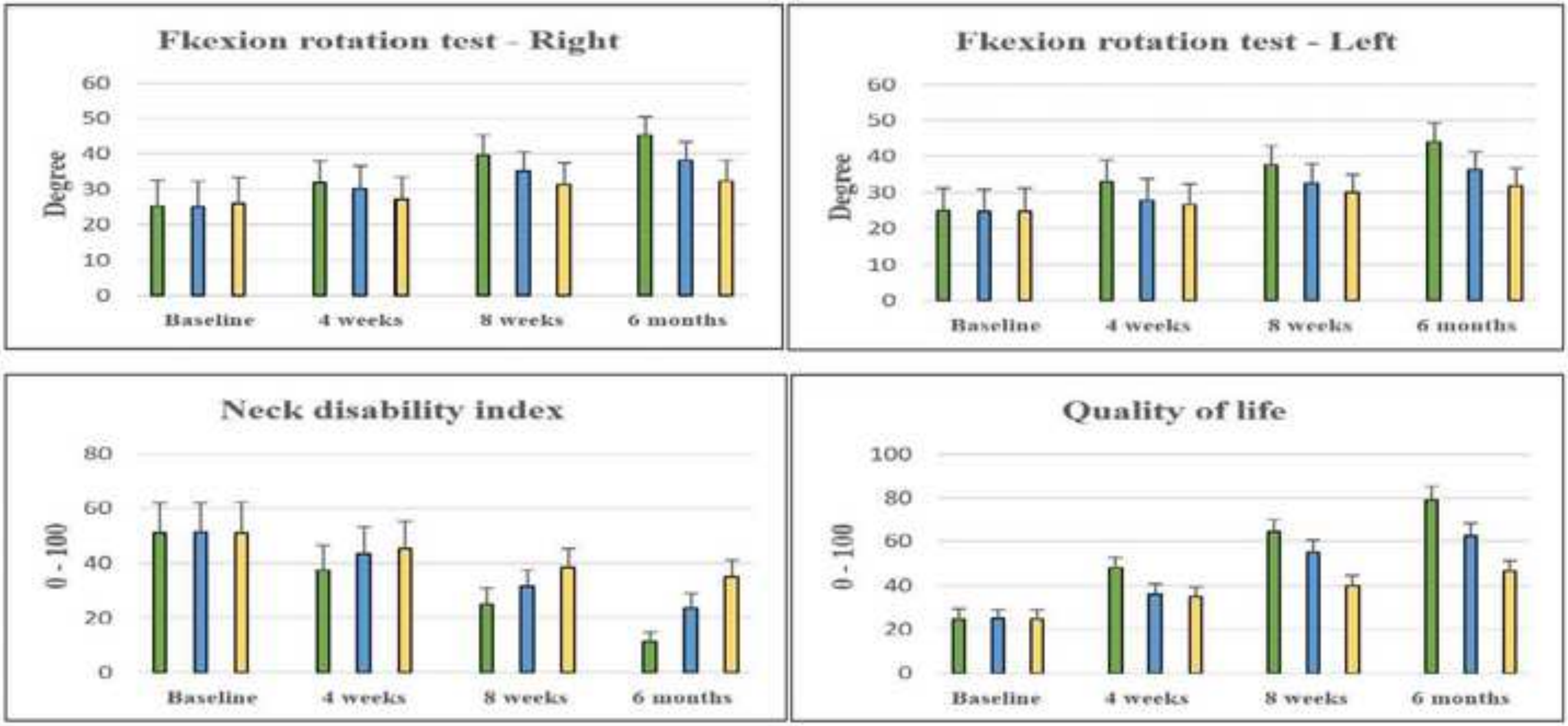

Cervical spine manipulation Thoracic spine manipulation Conventional physiotherapy

Figure 1 CONSORT Study flow chart

Click here to access/download;Figure;Figure 1.tiff

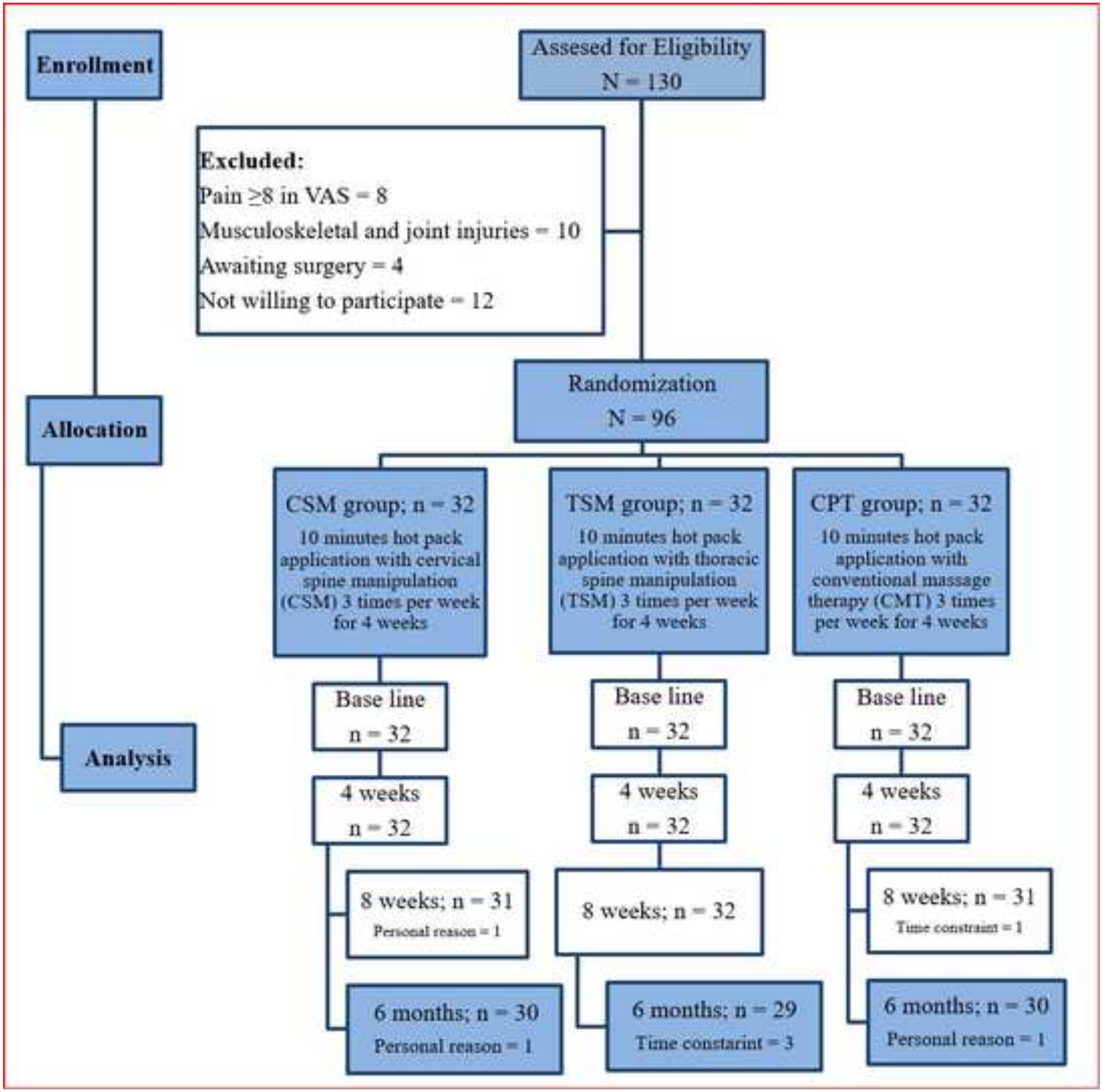

Figure 2

Click here to access/download;Figure;Figure 2.tiff

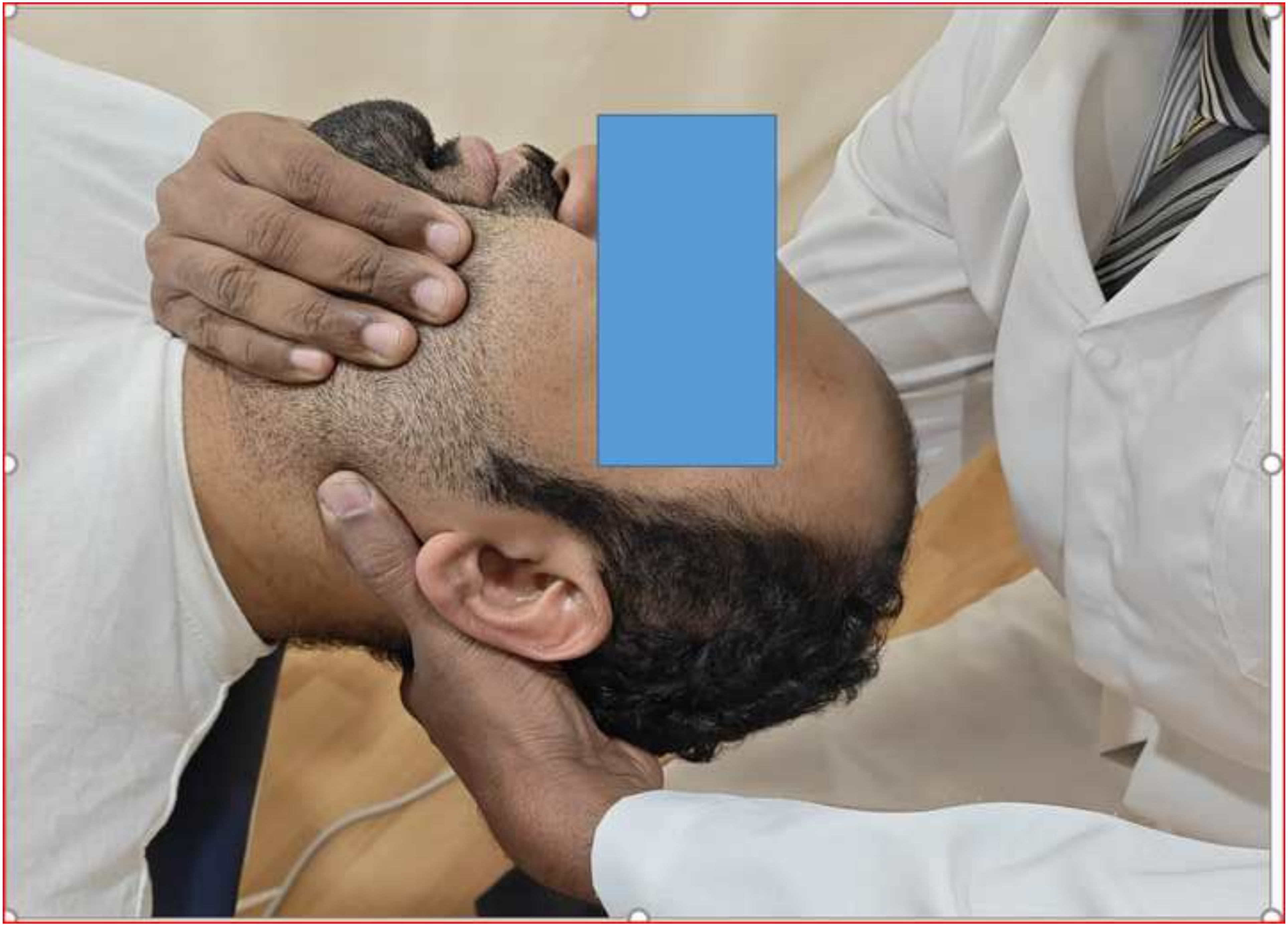

Click here to access/download;Figure;Figure 3.tiff

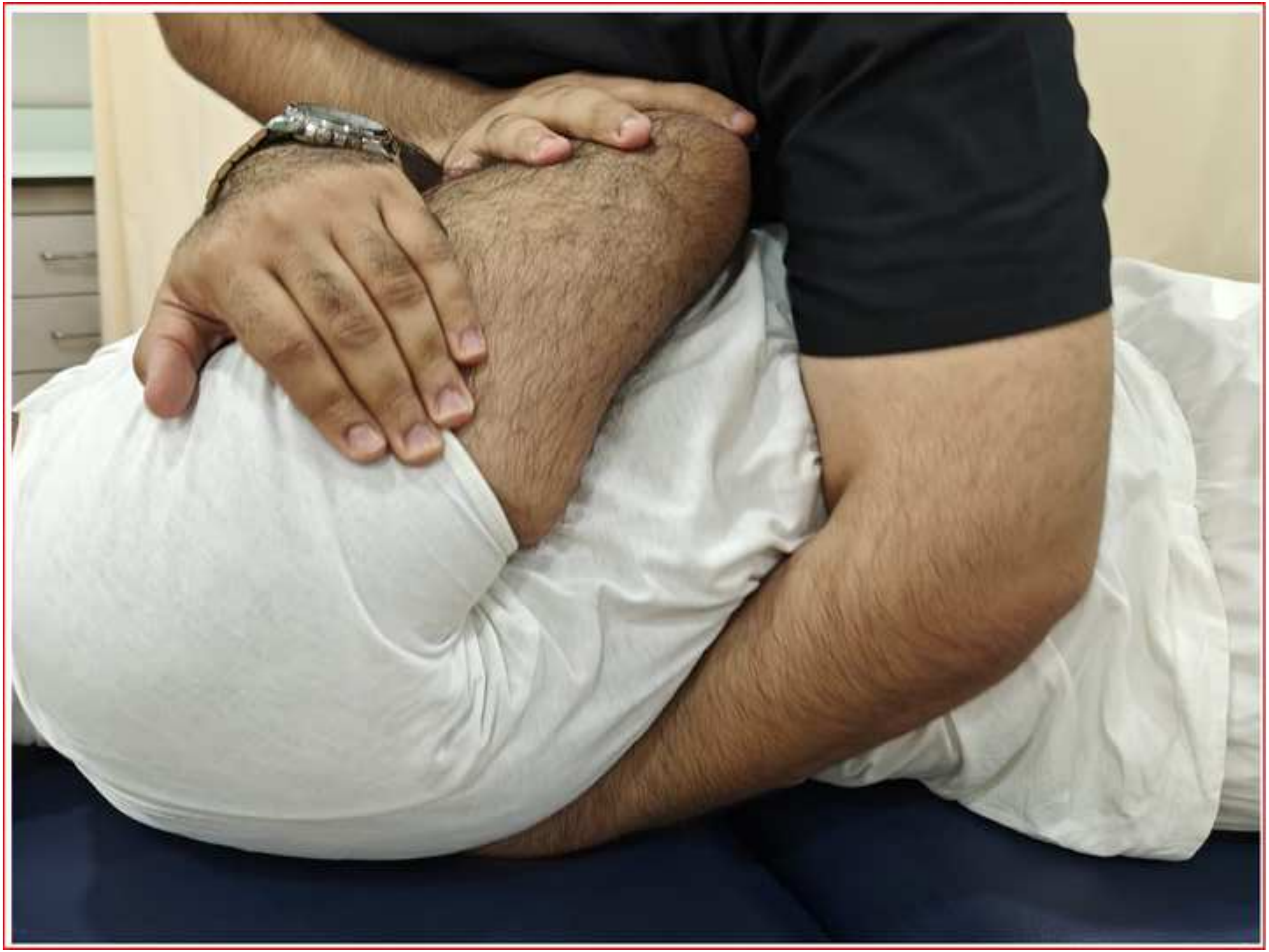

Figure 4

Click here to access/download;Figure;Figure 4.tiff

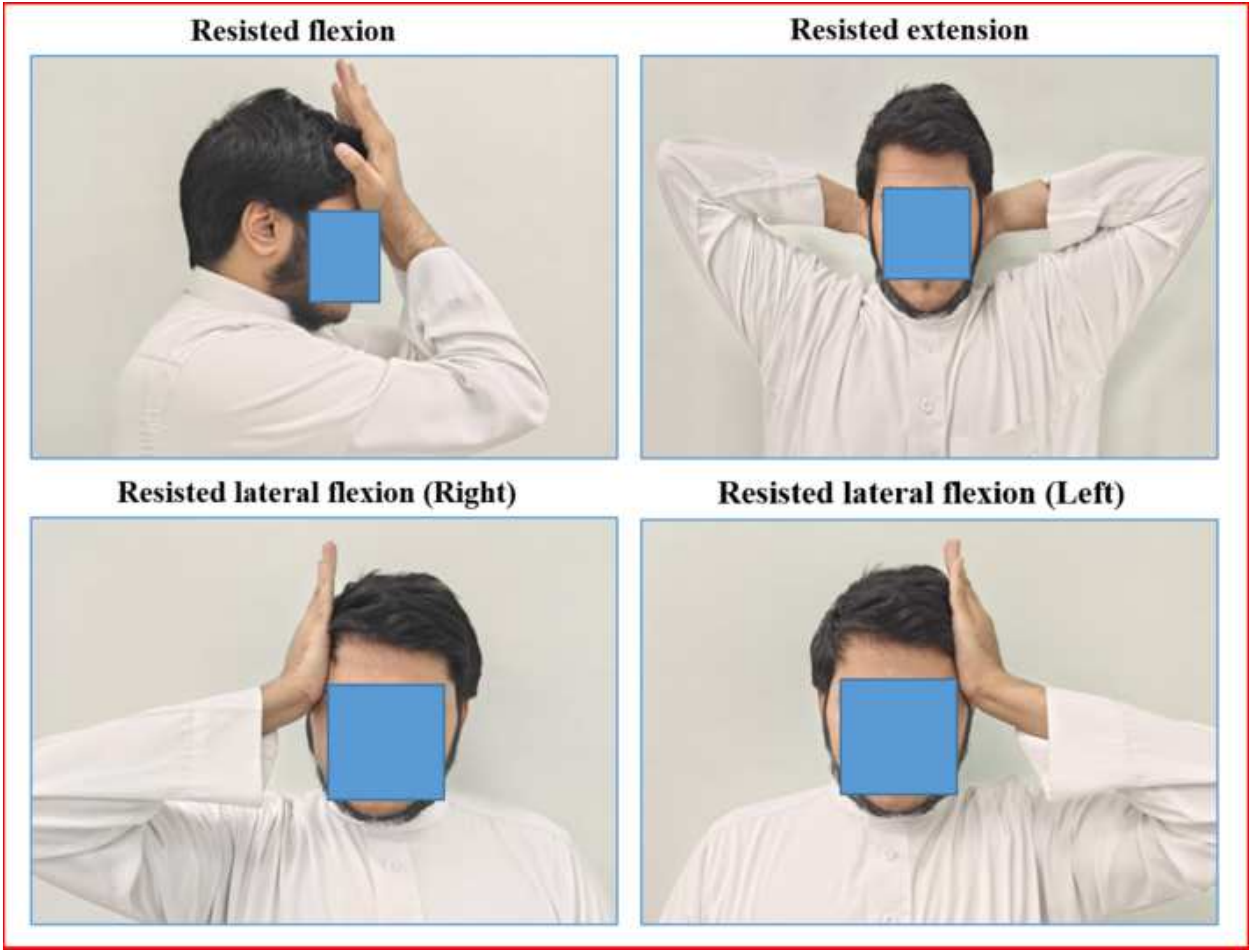

Figure 5a

Click here to access/download;Figure;Figure 5a.tiff

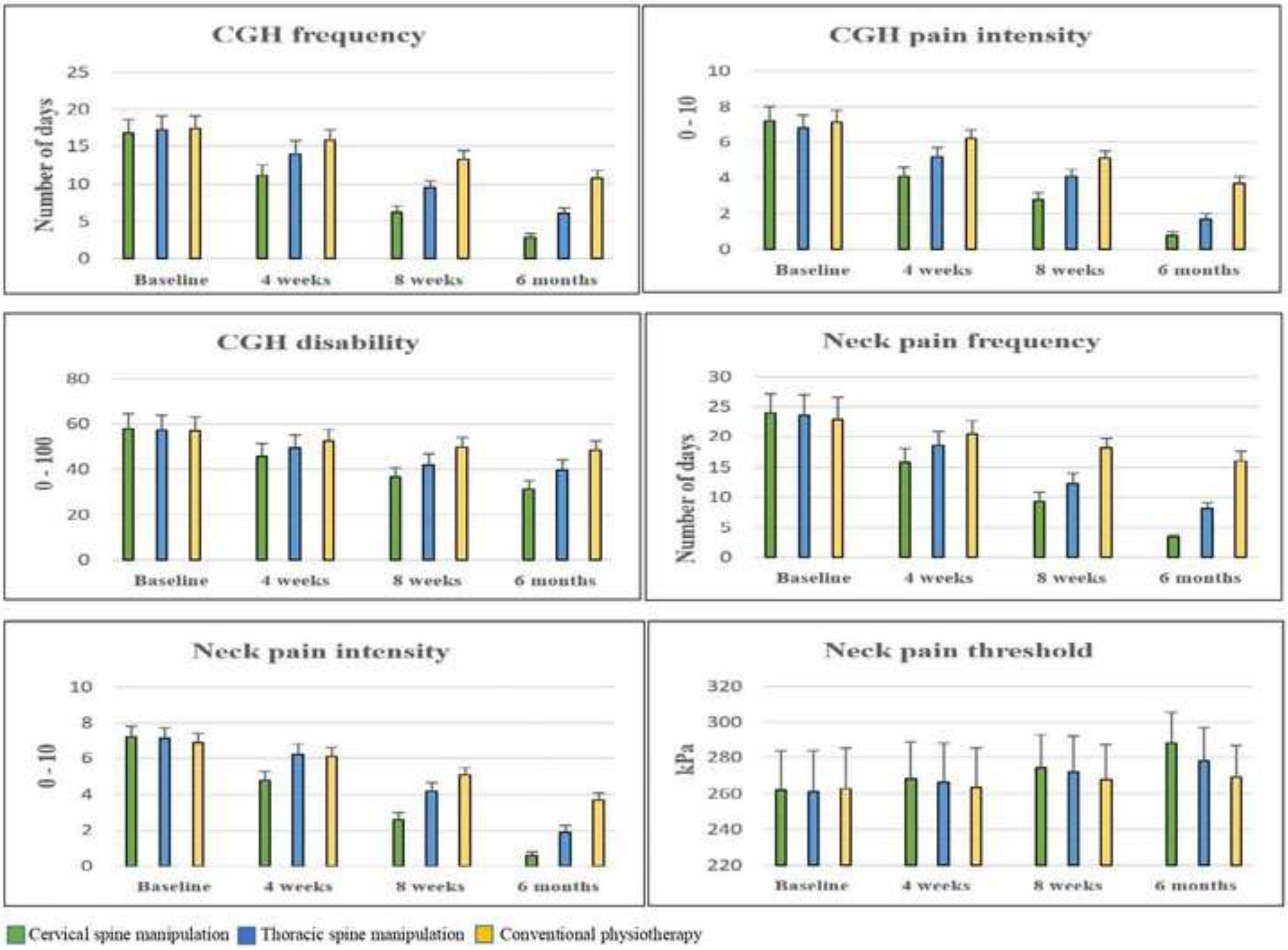

CONSORT Check list

Click here to access/download
Supporting Information
Consort 2010 checklist.docx

Click here to access/download
Supporting Information
Data.xlsx

Click here to access/download
Supporting Information
Trial protocol.docx

Ethical approval

Click here to access/download

Other

Ethical approval.pdf

Title: Comparative effectiveness of cervical vs thoracic spinal-thrust manipulation for care of cervicogenic headache: a randomized controlled trial.

Running Title: Manipulation techniques in Cervicogenic Headache.

Gopal Nambi,[1*] Mshari Alghadier[1], Mudathir Mohamedahmed Eltayeb[2], Osama R. Aldhafian[3], Ayman K. Saleh[3,4], Nesreen Alsanousi[5], Alaa Jameel A. Albarakati[6], Mohamed A. Omar[4], Mohamed Nagah Ahmed Ibrahim[4], Abdehamid A. Attallah[4], Mohammed Abdelgwad Ismail[4], Mohamed Elfeshawy[4]

[1] Department of Health and Rehabilitation Sciences, College of Applied Medical Sciences, Prince Sattam bin Abdulaziz University, Al-Kharj, Saudi Arabia.

[2] Department of Nursing, College of Applied Medical Sciences, University, Prince Sattam bin Abdulaziz University, Al-Kharj, Saudi Arabia.

[3] Department of Surgery, College of Medicine, Prince Sattam bin Abdulaziz University, Al-Kharj, Saudi Arabia.

[4] Department of Orthopedic surgery, Faculty of Medicine for Girls, Al-Azhar University, Cairo, Egypt.

[5] Department of Biochemistry, College of Medicine, Prince Sattam bin Abdulaziz University, Al-Kharj, Saudi Arabia.

[5] Department of Surgery, College of Medicine, Umm Al-Qura University, Al-Qunfudah Branch, Makkah, Saudi Arabia.

*Corresponding Author:

E-mail: physio_gopal@rediffmail.com

DECLARATIONS

Clinical trial registration: Registered in Clinical trial registry, India (www.ctri.nic.in) with registration number: CTRI/2020/06/026092 and registered prospectively.

Consent for publication: No individual data is contained in this publication.

Availability of data and materials: The minimal data set is available as a supporting file.

Competing interest: None declared

Funding: This study is supported via funding from Prince Sattam bin Abdulaziz University project number (PSAU/2023/R/1444). There was no additional external funding received for this study.

Author Contributions: Gopal Nambi (GN), Mshari Alghadier (MA), Mudathir Mohamedahmed Eltayeb (MM), Osama R. Aldhafian (OA), Ayman K. Saleh (AS), Nesreen Alsanousi (NA), Alaa Jameel A. Albarakati (AA), Mohamed A. Omar (MO), Mohamed Nagah Ahmed Ibrahim (MI), Abdehamid A. Attalah (AA), Mohammed Abdelgwad Ismail (MI) Mohamed Elfeshawy (ME).

Conceptualization: GN, MA, MM, OA, AS, NA, AA, MO, MI, AA, MI, ME. Data curation: MM, OA, AS, NA, AA, MO, MI, ME. Formal analysis: GN, MA, AS, NA, AA, MO, MI, MI, ME. Funding acquisition: GN, MA, MM, OA, MO, MI, AA, MI, ME. Investigation: GN, MA, MM, OA, AS, NA, AA, MO. Methodology: GN, MA, MM, OA, MO, MI, AA, MI. Project administration: MA, MM, AS, NA, AA, AA, MI, ME. Resources: MM, OA, AS, NA, AA, AA, MI, ME. Software: OA, AS, NA, AA, AA, MI, ME. Validation: MM, OA, AS, MO, MI, AA, MI, ME. Visualization: GN, MM, OA, AS, MO, MI, MI, ME. Writing—original draft: MM, OA, AS, NA, MI, AA, MI. Writing—review and editing: GN, MA, MM, OA, AS, NA, AA, MO, MI, AA, MI, ME.

Acknowledgments: This study is supported via funding from Deanship of scientific research, Prince Sattam bin Abdulaziz University, Al Kharj, Saudi Arabia.

Abstract

Background: There is ample evidence supporting the use of different manipulative therapy techniques for Cervicogenic Headache (CgH). However, no technique can be singled as the best available treatment for patients with CgH. Therefore, the objective of the study is to find and compare the clinical effects of cervical spine over thoracic spine manipulation and conventional physiotherapy in patients with CgH.

Design, Setting, Participants: It ~~is~~ a prospective, randomized controlled study conducted between July 2020 and January 2023 at the University hospital. N=96 eligible patients with CgH were selected based on selection criteria and they were divided into cervical spine manipulation (CSM; n=32), thoracic spine manipulation (TSM; n=32) and conventional physiotherapy (CPT; n=32) groups, and they received the respective treatment for four weeks. Primary (CgH frequency) and secondary (CgH pain intensity, CgH disability, neck pain frequency, neck pain intensity, neck pain threshold, flexion rotation test (right and left), neck disability index and quality of life) scores were measured. The effects of treatment at various intervals were analyzed using a 3 × 4 linear mixed model analysis (LMM), with treatment group (cervical spine manipulation, thoracic spine manipulation, and conventional physiotherapy) and time intervals (baseline, 4 weeks, 8 weeks, and 6 months), and the statistical significance level was set at $P < 0.05$.

Results: The baseline demographic and clinical variables did not show any significance difference (p>0.05). The reports of the CSM, TSM and CPT group were compared between the groups. Four weeks following treatment CSM group showed more significant changes in primary (CgH frequency) and secondary (CgH pain intensity, CgH disability, neck pain frequency, pain intensity, pain threshold, flexion rotation test (Right and left), neck disability index and quality of life) than TSM and CPT groups (p=0.001). The same gradual improvement was seen in CSM group when compared to TSM and CPT groups (p=0.001) in the above variables at 8 weeks and at 6 months follow up.

Conclusion: The reports of the current randomized clinical study found that CSM resulted in significantly better improvements in pain parameters (intensity, frequency and threshold) functional disability and quality of life in patients with CgH than thoracic spine manipulation and conventional physiotherapy.

Keywords: Cervicogenic headache; cervical spine; thoracic spine; manipulation; physiotherapy

Clinical trial registration: CTRI/2020/06/026092 trial registered prospectively on 24/06/2020.

Introduction

Globally, headache disorders affect approximately 66% of the population between the ages of 18 and 65 years at least once a year. Sixty-six percent[redacted] of men and fifty-seven percent[redacted] of women report headache at least once in their lifetime which reduces the quality of life, work productivity and increased costs to society.[1] Cervicogenic headache (CgH) is a distinct form of headache and accounts for 17.8% of all headaches, the prevalence rate is between 15 and 20% among cases of chronic headache.[2] The prevalence rate of CgH is 0.21% in females and 0.13% in males and has various causative factors.[3] It has a significant negative socioeconomic impact and is a burden on the community and public health.[4] The causative factor for the headache is located in the neck region and the pain is made worse by movements of the head and neck.[2] The most accepted mechanism of CgH is found between the trigeminal nerve and C1 – C3 nerves in the trigemino-cervical nucleus.[5] It usually arises from musculoskeletal structures such as the cervical vertebra, intervertebral disc, or paravertebral muscles. The clinical features of CgH include unilateral or bilateral headache, limited range of motion (ROM) of the neck, and radiating pain to the head or face region. [6]

Generally, CgH is diagnosed on the basis of a detailed history and clinical assessment.[7] Physical examinations typically reveal pain in the cervical region – neck pain (NP), decreased neck movements, upper quarter muscle tightness and loss of muscle properties such as excitability, contractility, extensibility and elasticity.[8] The cervical flexion rotation test (CFRT) is a valid, reliable and accurate method with an overall diagnostic accuracy of 91% for assessing cervical range of motion in patients with CgH.[9] The management of CgH consists of pharmacological and non-pharmacological methods, in which the pharmacological means are associated with many side effects such as damage to liver or kidneys, diarrhea, constipation and allergic reactions.[10] There are also many non-pharmacological treatment modalities available such as; physical modalities, positional release therapy (PRT), muscle strengthening

exercises, ergonomic guidance and patient education etc.[11] It has been estimated that 34% of US citizens receive some sort of physiotherapy for CgH each year.[12]

In physiotherapy, joint mobilization and manipulation are the most commonly used treatment modality for treating patients with CgH. [13] The manipulation technique commonly used to treat CgH target two different regions in the spine such as cervical and thoracic spine. Cervical spine manipulation (CSM) and thoracic spine manipulation (TSM) technique uses high velocity, low amplitude thrusts (HVLAT) maneuver. Some studies have looked solely at the effects of manipulating the cervical spine in cases of Cervicogenic headache. [6,14-15] Dunning JR et al investigated that six to eight sessions of upper cervical and upper thoracic manipulation were shown to be more effective than mobilization and exercise in patients with CgH, and the effects were maintained at 3 months.[16] Haas et al. investigated the effectiveness of cervical manipulation in patients with CgH.[17] Similarly, McDevitt AW et al. found that thoracic spine manipulation alone significantly improved neck-related disability in CgH, but had no effect on headache-related disability but participants reported overall improvement of their condition.[18] However, so far no studies have compared and investigated the individual effects of cervical spine manipulation, thoracic spine manipulation or conventional physiotherapy in treating patients with CgH.

Numerous studies have supported the application of various manipulative therapy approaches for the treatment of CgH. [13-18] Nevertheless, there is a scarcity of evidence in comparing the individual effects of cervical and thoracic manipulation approaches in Cervicogenic headache, particularly regarding its clinical and functional aspects. Additionally, no studies have attempted to address the shortcomings and gaps observed in the existing literature on the management of CgH, such as a lack of comparison between manipulation in two different regions, poor study designs, quality and small sample sizes. Therefore, our study objective was to compare and investigate the individual effects of cervical and thoracic manipulation techniques on patients with ~~Cervicogenic headache~~. This randomized clinical trial hypothesized that there ~~is~~ a difference in primary and secondary outcome measures between cervical spine manipulation, thoracic spine manipulation, and conventional physiotherapy for treating of patients with CgH.

Materials and methods

Study design

The trial was a parallel-group, prospective, randomized controlled trial. The required participants were screened and diagnosed by a physician at the University hospital between 1st July 2020 and 31st July 2022 in accordance to the Cervicogenic headache diagnostic criteria 11.2.1 from the ICHD-3 (International Classification of Headache Disorders) [19] and the disease CgH falls under the International classification of disease -10 (ICD-10) code of G44. 841.[7] Ninety-six (N=96) participants who fulfilled the eligibility criteria were randomly allocated into three groups equally: the cervical spine manipulation (CSM; n=32), thoracic spine manipulation (TSM; n=32), and conventional physiotherapy (CPT; n=32) groups through a computer-generated simple random table and the allocation of the participants to each group was concealed using sealed envelopes. The computer did not generate the group until it was time to randomize each participant, ensuring that the allocation was concealed. No significant changes were made while the study was being carried out because it was designed as a follow-up to a pilot study and the 6-month follow-up data collection was completed on 31st January 2023.

The research was conducted at #####, and the Department Ethical Committee (DEC) granted ethical approval under the reference number RHPT/019/042. The DEC accepted the study protocol as well as the informed consent forms. The study involved human participants and followed the instructions outlined in the Declaration of Helsinki (1964) and was prospectively registered in clinical trial.gov.in CTRI/2020/06/026092 on June 24, 2020.

Participants

Participants aged between 18-60 years and suffering from unilateral or bilateral CgH (>3 months) were allowed to participate in the study. Patients with pain intensity ≥3 on a visual analog scale (VAS), CgH resulting from pain in the neck followed by headache, limited neck movements, neck stiffness and cervical spine disorders were allowed to participate in the study. Other primary headaches such as migraine and tension-type headaches (TTH), whiplash injuries, participants who show signs of the five ‘D’s’ (dizziness, drop attacks, dysarthria, dysphagia, diplopia) or who have signs of the three ‘N’s (nystagmus, nausea, other neurological symptoms (cord compression or nerve root involvement), contraindications to manipulative therapy (tumour, degenerative and inflammatory arthritis, osteoporosis, dislocation, fractures, and steroid intake), underwent previous head and neck surgeries, had physiotherapy or other

complementary therapies in the last three months were excluded. The flow of the study program was documented by following the CONSORT guidelines and it was displayed in (Fig 1). [20]

Fig 1. Flow chart showing the study details.

Interventions

Certified physiotherapists having 10 – 15 years of experience in providing spinal manipulation for patients with CgH provided the treatment to both groups. All the participants in the three groups had given their informed consent to participate in the study after understanding the detailed information about the study protocol. All the participants in the three groups received 10-minutes of hydro collator pack application to relax the muscles of the area around the neck and upper back. Following this, the participant's neck muscles and joints were assessed for any musculoskeletal dysfunction. After that, the participants were given the manipulation techniques as per the directions provided in the study protocol. Standardized treatment techniques were used for all the group participants to reduce intervention bias. The procedures of intervention and follow-up measurements were recorded in standardized forms. During the study period, the participants were asked to refrain from taking any other type of intervention, they received the concerned interventions 3 times per week for 4 weeks.

Spinal manipulation therapy

Peterson and Bergman defined spinal manipulation therapy (SMT) is a high-velocity low-amplitude thrust (HVLAT) technique. [21] Four experienced physiotherapists having experience in SMT performed this technique after evaluation of each participant by physical examination and palpation. Then the therapist manipulated the upper cervical (C1-2) spine for the CSM group and upper thoracic (T1-T2) spine for the TSM group by following the study protocol irrespective of joint dysfunction. For both the upper cervical (C1-2) and upper thoracic (T1-T2) manipulations, if no popping or cracking sound was heard on the first attempt, the therapist repositioned the patient and performed a second manipulation. A maximum of 2 attempts were performed on each patient. The clinicians were instructed that the manipulations are likely to be accompanied by multiple audible popping sounds. [16] If any participant reported any new red flag signs or showed no signs for manipulation, such as no pain or musculoskeletal dysfunction, then the procedure was not performed.

Cervical spine manipulation (CSM)

The patient was asked to lie comfortably in a supine position, in which the manipulation targeted the C1–C2 vertebra. The patient's head was kept in a "cradle hold" method" where the head was free from the treatment table. The left posterior arch of the C1 vertebra (atlas) was held with the lateral aspect of the proximal phalanx of the therapist's index finger of the left hand. The right hand of the therapist holds the chin of the patient. To localize the forces to the left C1-C2 vertebral articulation, the patient was positioned using extension, a posterior-anterior (PA) shift, ipsilateral side-bend and contralateral side-shift. While maintaining this position, the therapist performed a single high-velocity, low-amplitude thrust (HVLAT) manipulation to the left atlanto-axial joint using right rotation in an arc toward the underside eye and translation toward the table. This was repeated using the same procedure but directed to the right C1-C2 articulation. The selection of the spinal segments to target was prescriptive (C1–C2) and it was based on the study protocol (Fig 2). The manipulation was done first on the pain-free side and then on the painful side and the rotation range was limited by the target vertebra. [6]

Fig 2. Figure showing the cervical spine manipulation.

Thoracic spine manipulation (TSM)

The patient was asked to lie comfortably in a supine position and the manipulation targeted the T1–T2 vertebra. For this technique, the patient held her/his arms and forearms across the chest with the elbows aligned in a supero inferior direction. The therapist contacted the transverse processes of the lower vertebrae of the target motion segment with the thenar eminence and middle phalanx of the third digit. The upper lever was localized to the target motion segment by adding rotation away and side-bend towards the therapist while the underside hand used pronation and radial deviation to achieve rotation toward and side-bend away moments, respectively. The space inferior to the xiphoid process and costo-chondral margin of the therapist was used as the contact point against the patient's elbows to deliver a manipulation in an anterior to posterior direction targeting T1-2 bilaterally (Fig 3). [18]

Fig 3. Figure showing the thoracic spine manipulation.

Conventional physiotherapy (CPT)

The participants of the CPT group received massage therapy for 15 minutes using Queen Helene, Cocoa Butter face & body cream, New York, USA. The participant was asked

to lie down in a prone position, with the anterior aspect of the head is resting on a face hole in the couch. The treating therapist stands by the patient's head side and uses the tips of the middle fingers of both hands to perform circular kneading on both sides of the C1 to C7 vertebra. This maneuver was repeated 3 times for each cervical vertebra, beginning from the C7 vertebra and working towards the C1 vertebra. Then the head was turned to right side, and the circular kneading was performed on the sub-occipital and paravertebral muscles and the same procedure was done in the left side of the neck. [22]

Participants in all the three groups received ten minutes of hydro collator heat prior to intervention (manipulation or massage). Also they were asked to perform neck isometric exercises three times a day, every day for 4 weeks. The patient was asked to keep his hand over his forehead and resist the forward movement of his neck for 10 seconds and the same movement was repeated 15 times. Similarly, the patient was asked to keep the hand on the posterior and lateral sides of the head, and resist the backward and sideways movements of the neck (Fig 4). Also, static active stretching exercises for the upper trapezius, levator scapulae, scalene, and sternocleidomastoid muscles were taught to the patients, which was maintained for the 30s with 3 repetitions. [23] The patients were instructed to keep doing this set of exercises after 4 weeks of various intervention protocols and they were asked to maintain an exercise log book to check the treatment compliance.

Fig 4. Figure showing the neck isometric exercises.

Outcomes

All the outcome measures were recorded by a ~~blinded~~ physiotherapist, and the scores were entered in a data sheet. The scores were measured at the beginning of the study, after 4-weeks, 8-weeks, and at 6-months.

Primary outcome

CgH frequency: It is a self-administered outcome variable where the patient enters ~~his~~ CgH pain experience in a medical log book every evening to find the number of painful days in 4-weeks. [24]

Secondary outcome

CgH pain intensity: The pain intensity of CgH was assessed using visual analogue scale (VAS). Patients rated their typical level of pain status during the previous week on a 10 cm

horizontal line, with one end 0 representing "no pain" and the other end 10 representing "worst pain imaginable." [25]

CgH disability: The Headache Impact Test (HIT) questionnaire is a valid and reliable tool to assess the level of disability in CgH patients. It consists of six items: pain, social functioning, role functioning, vitality, cognitive functioning, and psychological distress. Each item is rated using 5 response categories (never, rarely, sometimes, very often, or always), each category of which is associated with a numerical value (6, 8, 10, 11, and 13, respectively), resulting in a range of possible total summed scores of 36-78. The score categories are no or mild disability (49 or less), moderate disability (50–55), severe disability (56–59), and complete disability (60–78). [26]

NP frequency: It is a self-administered outcome variable where the patient enters his neck pain experience in a medical log book every evening to find the number of painful days in 4-weeks. [24]

NP intensity: The pain intensity of neck pain was assessed using visual analogue scale (VAS). Patients rated their average pain intensity over the past week on a 10 cm horizontal line, with one end 0 representing "no pain" and the other end 10 representing "worst pain imaginable." [25]

NP pressure threshold: It is the lowest intensity at which a given stimulus is perceived as painful and it was measured using an instrument called an Algometer (Baseline, 22-pound dolorimeter, ID, USA). The tip was placed over the fixed points (trigger point on the upper trapezius muscle) on the neck region which is identified through palpation techniques. It is a reliable and valid tool for determining pain threshold. [27]

Cervical flexion–rotation test (~~FRT):~~ The cervical flexion–rotation test is done with the patient in a supine lying position. The therapist passively maintains the patient's neck into full flexion to relax the structures of the middle and lower cervical spine, then the patient's head is passively rotated in each direction while the flexed position is maintained and the range of motion is measured. [9]

Neck disability index (NDI): It is a reliable and valid self-reported questionnaire with ten items scored on a 0 to 5 scale. The grades of disability are determined based on the scores obtained,

which are as follows: 10-29% mild; 30-49% moderate; 50-69% severe; 70% or more is a complete disability. [28]

Quality of life: The EQ-5D (Euro Qol 5D) is self-administered health related quality of life (HRQOL) questionnaire, which measures the five dimensions of quality of life. It includes mobility, self-care, usual activities, pain/discomfort, and anxiety/depression. It is used to assess the CgH patients' overall quality of life with a scale of 0 (worst) to 100 (best). [29]

Sample size

For calculating the number of subjects to be include in the study, the primary outcome measure CgH frequency in days was taken into consideration based on previous pilot study which found the effect of spinal manipulation in the treatment of CgH, with 10 subjects in each group. Using the G-Power software (version 3.1.9.2; Franz Faul, University of Kiel, Germany), assuming a two-sided $\alpha = 0.05$, and power ($1-\beta = 0.80$), to detect an effect size of 1.2 CgH days and mean difference of 4 CgH days (between groups) and a standard deviation of 0.5, an approximately 28 samples were required. In assuming 10% dropout, we enrolled 32 subjects in each group.

Blinding

Because of the experimental nature of the study methodology, it was not feasible to blind the treating therapist as well as the participants of the study. The therapists who assessed the outcome variables at baseline, 4-weeks, 8-weeks, and 6-months were blinded. Therefore, the therapist providing the treatment and the therapist measuring the data were different individuals. In addition, the outcome-measuring therapist continued to be masked to the participant's groups at all-time intervals. Nevertheless, for the primary outcome measurement, the assessor was the participant, so the study cannot properly be called assessor-blind either. Also, participants were asked not to discuss their treatment details with their peers or the outcome-measuring therapist. In addition, authors did not have access to information that could identify individual participants during or after data collection.

Statistical methods

The normality of study participants' demographic characteristics was analyzed through the Kolmogorov-Smirnov test. The outcome data were presented in the form of a mean and standard deviation with a 95% confidence interval. The study followed the intention to treat principle method by including the participants' missing follow-up data in the data analysis. The effects of treatment at different time intervals were analyzed using a 3 × 4 linear mixed model analysis (LMM), with patient as a random factor and treatment groups; cervical spine manipulation, thoracic spine manipulation, and conventional physical therapy and time intervals; baseline, four weeks, eight weeks, and at six months) as fixed factors. Following the omnibus 3x4 LMM model analysis, post hoc Bonferroni analysis was done to compare the three study groups pair-wise. For all the statistical tests a statistical significance level of $\alpha=0.05$ was set and the software GraphPad-Prism (version 9.1), Boston, MA, USA was used for analysis.

Results

Participants

Out of the 130 participants screened, eight had a VAS score greater than 8, ten participants were having some sort of orthopaedic injuries, four participants were undergone joint surgeries, and twelve refused to involve in the research and were excluded. N=96 participants were chosen based on the eligibility criteria and allocated to one of the three groups. Two participants in the CSM and CPT groups, and three in the TSM group, did not complete the 4-week treatment program with a 6-month follow-up (Fig 1). Compliance with follow-up data collection at 6 months was 93%, adherence to study protocols (e.g., number of visits) was 100%, and none of the participants in the three groups received any additional care that was not included in the three study interventions. At baseline, the demographic characters such as age (years), height (cm), weight (kg), and BMI (kg/m^2) scores did not report statistically significant variation between the groups ($p>0.05$). In all three groups, females (53-56%) are affected more than males. At baseline, the clinical variables also did not show any significant difference between groups (p0.05). The clinical presentation of headache is more unilateral (78% - 84%) than bilateral, and the majority of CGH cases have associated neck pain (84% - 88%). (Table 1). Due to random sample design the three groups were uniform in baseline characteristics and the baseline outcome variables.

Primary outcome

The mean and standard deviation (SD) of the CgH frequency score between the three groups, at four-time periods, was shown in Table 2 and 3. The 3 × 4 linear mixed model (LMM) analysis reported statistically significant change in CgH frequency (p=0.012). Over 4 weeks of different interventions, there is a significant change in CgH pain frequency level between the CSM (7.9; CI 95% 7.41 to 8.38), and TSM (4.7 CI 95% 4.21 to 5.18), groups (p=0.001). A similar improvement can be seen in 8-weeks and at 6-months' measurement. The post-hoc Bonferroni analysis and the standard mean difference showed more percentage of improvement in CgH pain frequency in the CSM group than TSM and CPT groups (Fig 5a). The complete interpretation shows a slight leaning towards the CSM group than TSM and CPT group in CgH frequency.

Fig 5a. Pre and post outcome measures of CSM, TSM and CPT groups.

Secondary outcomes

The mean and standard deviation (SD) of the secondary outcomes between the three groups, at four-time periods, was shown in Table 2 and 3. The 3 × 4 linear mixed model showed statistically significant variation in CgH pain intensity (p = 0.022), CgH disability (p = 0.043), neck pain frequency (p = 0.007), neck pain intensity (p = 0.002), neck pain threshold (p = 0.015), flexion rotation test (Right and left) (p = 0.008, 0.006), neck disability index (p = 0.003) and quality of life (p = 0.004).

After 4 weeks of intervention, there are statistically significant variations in CgH pain intensity, CgH disability, NP (frequency, intensity, and threshold), FRT (Right and left), NDI, and QoL score between the CSM (2.9; CI 95% 2.71 to 3.08), (17.1; CI 95% 14.7 to 19.6) (12.7; CI 95% 12.08 to 13.31), (3.1; CI 95% 2.89 to 3.30), (-18.7; CI 95% -29.18 to -8.21), (-12.9, -12.31; CI 95% -16.12 to -9.67, -15.30 to -9.31), (23.64; CI 95% 20.55 to 26.72), (-32.5; CI 95% -35.89 to -29.10) and TSM (2.0; CI 95% 1.81 to 2.18), (8.8; CI 95% 6.3 to 11.2), (7.9; CI 95% 7.28 to 8.51), (1.8; CI 95% 1.59 to 2.00), (-8.8; CI 95% -19.28 to 1.68), (-5.91, -4.31; CI 95% -9.13 to -2.68, -7.30 to -1.31), (11.36; CI 95% 8.27 to 14.44), (-15.7; CI 95% -19.09 to -12.30) groups (p<0.001) respectively. The post hoc Bonferroni analysis and the standard mean difference showed more percentage of improvement in the secondary outcomes in the CSM

group than TSM and CPT groups (Fig 5a and 5b). The complete interpretation shows a slight leaning towards the CSM group than TSM and CPT group in all the secondary variables.

Fig 5b. Pre and post outcome measures of CSM, TSM and CPT groups.

Discussion

This is the first powered randomized clinical study to find the individual and comparative effects of cervical and thoracic manipulation for patients with CgH. The reports of the study show that cervical manipulation was superior to thoracic manipulation and conventional PT (massage) in treating patients with CgH. According to this study, after four weeks of intervention the cervical spine manipulation (CSM) group showed statistically significant changes in all the outcome measures in CgH patients. When compared to TSM and conventional physical therapy, CSM is more effective in reducing the primary outcome CgH frequency, which was clinically important. The same improvements were noted in all the other outcome measures such as CgH pain, neck pain frequency, neck pain intensity, neck pain threshold, range of motion, and quality of life and these reports were supported by Dunning JR et al study. [16] So far, no studies have looked at the changes in CgH disability and neck disability, but this study looked at the benefits of cervical spine manipulation over thoracic spine manipulation in CgH patients. However, these findings were contradictory to the observation of Borusiak et al. [30]

~~According to our findings, HVLAT technique in the cervical vertebra may promote the afferent nerve fiber activity via joint receptors. It improves the overall action and properties of the neck muscles by activating the alpha motor neuron. [31] It alters the sensory fiber activity by activating the joint receptors, thereby changing the α-motor neuron activity levels and subsequent muscle reaction.~~ Because of the high mobility of the cervical spine, CSM can stimulate the receptors of deep neck muscles and sub-occipital muscles, which TSM is not able to do. [32] Other theories for the pain-modulating effects of cervical manipulation included biomechanical, vertebral (temporal summation), and neural (central descending pain inhibitory pathway) mechanisms which were noted by Bialosky JE et al and Haavik-Taylor H et al. [6,33-34]

Our findings also showed that thoracic spine manipulation improved both primary and secondary outcomes in CgH patients. TSM was also helpful in improving the pain parameters, functional disability and quality of life significantly. It was supported by McDevitt AW et al

[18] and suggests that TSM had no effect on headache-related disability but resulted in significant improvements in neck-related disability and participant reported perceived improvement. Although several types of research have been conducted to find the effects of different types of spinal manipulation, the real mechanical and neuro-physiological alterations behind these changes have not yet been found and are unclear, but Bialosky et al. suggest that the effects may be due to potential neurophysiological and biomechanical effects, as well as possibly placebo effect. [33] According to Suvarnnato T et al, the neurophysiologic response of pain reduction in patients with chronic neck pain through TSM is that it induces a reflex inhibition of pain or muscle relaxation reflex by modifying the discharge of proprioceptive Group Iand II afferents. It also activates descending inhibitory mechanisms resulting in pain reduction in distant areas from the manipulation. Through these mechanisms, the thoracic manipulation may induce ventral periaqueductal gray (vPAG) in the brain, which activates endogenous opioid peptides resulting in pain reduction in different areas. [35] The little changes in the conventional physical therapy group on pain intensity and other outcome variables explained the analgesic effect of CPT on cervicogenic headache. Application of massage on the trigeminal-cervical area reduces inflammatory responses, reduces neural sensitivity and plays a significant function in decreasing the tension of the sub-occipital and para-vertebral cervical spine muscles, which is another important mechanism of physical therapy on CgH patients. [36-37] The findings of this trial would assist physiotherapists in making decisions to select the best manual therapy approach for CgH patients.

Limitations

The study had some limitations during its execution, which should be considered for future studies. First, the study included both genders, but the reports collected were not calculated independently during the statistical analysis, these gender differences may influence the research reports. Second, it is impossible to ensure that the subjects completed the questionnaires daily rather than after a week or four weeks. The standard for headache outcomes is a headache diary that uses technology to ensure that frequency and pain intensity are reported immediately on the day stipulated, not added in later. Third, this study lacks a placebo group to determine the true effects of treatment groups. The beneficial effects of various manipulation techniques on pain and other symptoms in Cervicogenic headache were investigated. Fourth, the differences in the length of study visits between groups adds attention bias and could reduce differences between manipulation groups and the massage group.

Finally, the treatment preference of physiotherapists and patients was not asked which could have affected the results due to clinical equipoise.

Conclusion

The reports of the current randomized clinical study found that cervical spine manipulation resulted in significantly better improvements in pain parameters (intensity, frequency and threshold), functional disability and quality of life in patients with cervicogenic headache than thoracic spine manipulation and conventional physiotherapy. The report also provided an additional evidence in the field of manual therapy for patients with cervicogenic headache. Future studies are recommended to find the biomechanical and biochemical reactions behind the clinical and functional changes of these manipulation techniques in treating patients with CgH symptoms.

List of abbreviations: CgH - Cervicogenic Headache, ROM - range of motion, FRT - flexion rotation test, ICHD - International Classification of Headache Disorders, CSM - cervical spine manipulation, TSM - thoracic spine manipulation, CMT - conventional massage therapy, HVLAT - high-velocity low amplitude thrust, NDI - Neck disability index, QOL - Quality of life

References

1. AlQarni MA, Fayi KA, Al-Sharif MN, Siddiqui AF, Alhazzani AA. Prevalence of migraine and non-migraine headache and its relation with other diseases in the adults of Aseer Region, Saudi Arabia. *J Family Med Prim Care*. 2020;9(3):1567-1572.
2. Verma S, Tripathi M, Chandra PS. Cervicogenic Headache: Current Perspectives. *Neurol India*. 2021;69(Supplement):S194-S198.
3. Chaibi A, Russell MB. Manual therapies for cervicogenic headache: a systematic review. *J Headache Pain*. 2012;13(5):351-359.
4. Stovner L, Hagen K, Jensen R, et al. The global burden of headache: a documentation of headache prevalence and disability worldwide. *Cephalalgia*. 2007;27(3):193–210.
5. Pourahmadi M, Mohseni-Bandpei MA, Keshtkar A, et al. Effectiveness of dry needling for improving pain and disability in adults with tension-type, cervicogenic, or migraine headaches: protocol for a systematic review. *Chiropr Man Therap*. 2019;27:43.

6. Lerner-Lentz A, O'Halloran B, Donaldson M, Cleland JA. Pragmatic application of manipulation versus mobilization to the upper segments of the cervical spine plus exercise for treatment of cervicogenic headache: a randomized clinical trial. *J Man Manip Ther*. 2021;29(5):267-275.
7. Sjaastad O, Fredriksen TA, Pfaffenrath V. Cervicogenic headache: diagnostic criteria. The Cervicogenic Headache International Study Group. Headache. 1998;38(6):442–5.
8. Hall T, Briffa K, Hopper D. Clinical evaluation of cervicogenic headache: a clinical perspective. *J Man Manip Ther*. 2008;16(2):73-80.
9. Hall TM, Briffa K, Hopper D, Robinson K. Comparative analysis and diagnostic accuracy of the cervical flexion-rotation test. *J Headache Pain*. 2010;11(5):391-397.
10. Bogduk N.Govind J., Cervicogenic headache: an assessment of the evidence on clinical diagnosis, invasive tests, and treatment. Lancet Neurol, 2009. 8(10): p. 959–68.
11. Page P. Cervicogenic headaches: an evidence-led approach to clinical management. *Int J Sports Phys Ther*. 2011;6(3):254-266.
12. Clarke TCBL, Stussman BJ, Barnes PM, Nahin RL. Trends in the use of complementary health approaches among adults: United States, 2002–2012. Natl Health Stat Rep. 2015;79:1–16.
13. Fernández-de-Las-Peñas C, Courtney CA. Clinical reasoning for manual therapy management of tension type and cervicogenic headache. *J Man Manip Ther*. 2014;22(1):44-50.
14. Haas M, Bronfort G, Evans R, Schulz C, Vavrek D, Takaki L, Hanson L, Leininger B, Neradilek MB. Dose-response and efficacy of spinal manipulation for care of cervicogenic headache: a dual-center randomized controlled trial. Spine J. 2018 Oct;18(10):1741-1754.
15. Nilsson N, Christensen HW, Hartvigsen J. The effect of spinal manipulation in the treatment of cervicogenic headache. *J Manipulative Physiol Ther*. 1997;20(5):326-330.
16. Dunning JR, Butts R, Mourad F, Young I, Peñas CF, Hagins M et al. Upper cervical and upper thoracic manipulation versus mobilizationand exercise in patients withcervicogenic headache: a multi-center randomized clinical trial. BMC Musculoskeletal Disorders. 2016;17:64.
17. Haas M, Spegman A, Peterson D, Aickin M, Vavrek D. Dose response and efficacy of spinal manipulation for chronic cervicogenic headache: a pilot randomized controlled trial. Spine J. 2010;10(2):117–28

18. McDevitt AW, Cleland JA, Rhon DI, et al. Thoracic spine thrust manipulation for individuals with cervicogenic headache: a crossover randomized clinical trial. *J Man Manip Ther*. 2022;30(2):78-95.
19. The International Classification of Headache Disorders, 3rd edition. Cephalalgia. 2018, 38(1):1–211.
20. Butcher NJ, Monsour A, Mew EJ, et al. Guidelines for Reporting Outcomes in Trial Reports: The CONSORT-Outcomes 2022 Extension. *JAMA*. 2022;328(22):2252-2264.
21. Peterson DH, Bergmann T. Chiropractic technique: principles and procedures. 3rd ed. St. Louis: Mosby; Chapter 5, 2010, 246-275.
22. Diana Hopper, Yogita Bajaj, Chor Kei Choi, et al. A pilot study to investigate the short-term effects of specific soft tissue massage on upper cervical movement impairment in patients with cervicogenic headache. Journal of Manual and Manipulative Therapy 2013: 21(1); 18 – 24.
23. Nambi G, Alghadier M, Ebrahim EE, et al. Comparative Effects of Mulligan's Mobilization, Spinal Manipulation, and Conventional Massage Therapy in Cervicogenic Headache-A Prospective, Randomized, Controlled Trial. *Healthcare (Basel)*. 2022;11(1):107.
24. Bendtsen L, Bigal ME, Cerbo R, Diener HC, Holroyd K, Lampl C, Mitsikostas DD, Steiner TJ, Tfelt-Hansen P. International Headache Society Clinical Trials S: Guidelines for controlled trials of drugs in tension-type headache: second edition. Cephalalgia. 2010;30(1):1–16.
25. Jensen MP, Karoly P, Braver S. The measurement of clinical pain intensity: a comparison of six methods. Pain. 1986;27(1):117–26.
26. Kosinski M, Bayliss MS, Bjorner JB, Ware Jr JE, Garber WH, Batenhorst A, Cady R, Dahlof CG, Dowson A, Tepper S. A six-item short-form survey for measuring headache impact: the HIT-6. Qual Life Res Int J Qual Life Asp Treat Care Rehab. 2003;12(8):963–74.
27. Chesterton LS, Sim J, Wright CC, Foster NE. Interrater reliability of algometry in measuring pressure pain thresholds in healthy humans, using multiple raters. Clin J Pain. 2007;23(9):760–6.
28. Vernon H, Mior S. The neck disability index: a study of reliability and validity. J Manipulative Physiol Ther. 1991;14:7. 409–415.
29. Luo N, Johnson JA, Shaw JW, Feeny D, Coons SJ. Self-reported health status of the general adult U.S. population as assessed by the EQ-5D and Health Utilities Index. Med Care. 2005;43(11):1078–86.
30. Borusiak P, Biedermann H, Bosserhoff S, Opp J. Lack of efficacy of manual therapy in children and adolescents with suspected cervicogenic headache: results of a prospective, randomized, placebo-controlled, and blinded trial. *Headache*. 2010;50(2):224-230.

31. Bolton PS, Budgell BS. Spinal manipulation and spinal mobilization influence different axial sensory beds. Med Hypotheses. 2006;66(2):258–62.
32. Cassidy JD, Lopes AA, Yong-Hing K. The immediate effect of manipulation versus mobilization on pain and range of motion in the cervical spine: a randomized controlled trial. J Manipulative Physiol Ther. 1992;15(9):570–5.
33. Bialosky JE, Bishop MD, Price DD, Robinson ME, George SZ. The mechanisms of manual therapy in the treatment of musculoskeletal pain: a comprehensive model. Man Ther. 2009;14(5):531–8.
34. Haavik-Taylor H, Murphy B. Cervical spine manipulation alters sensorimotor integration: a somatosensory evoked potential study. Clin Neurophysiol. 2007;118(2):391–402. doi:10.1016/j.clinph.2006.09.014
35. Suvarnnato T, Puntumetakul R, Kaber D, Boucaut R, Boonphakob Y, Arayawichanon P, Chatchawan U. The effects of thoracic manipulation versus mobilization for chronic neck pain: a randomized controlled trial pilot study. J Phys Ther Sci. 2013 Jul;25(7):865-71.
36. Panneton WM, Gan Q, Livergood RS. A trigeminoreticular pathway: implications in pain. PloS one. 2011; 6: e24499.
37. Youssef EF, Shanb AS. Mobilization versus massage therapy in the treatment of cervicogenic headache: a clinical study. *J Back Musculoskelet Rehabil*. 2013;26(1):17-24.

Table 1: Demographic details of CSM, TSM and CPT groups.

Sr. No	Variable		CSM	TSM	CPT	p-value
**1**	Age (year)	-	35.6 ± 3.8	34.8 ± 3.2	36.2 ± 3.7	0.295*
**2**	Gender	Male	14 (44%)	15 (47%)	14 (44%)	-
		Female	18 (56%)	17 (53%)	18 (56%)	-
**3**	Height (cm)	-	164.6 ± 3.8	163.5 ± 4.1	165.3 ± 4.4	0.215*
**4**	Weight (m)	-	72.92 ± 4.2	71.21 ± 4.6	73.83 ± 4.5	0.061*
**5**	BMI (Kg/m^2)	-	24.3 ± 2.23	24.5 ± 1.92	23.8 ± 2.01	0.378*
**6**	CgH duration (year)	-	6.6 ± 2.9	6.8 ± 3.1	5.9 ± 3.2	0.471*
**7**	CgH frequency (per day)	-	0.74 ± 0.15	0.72 ± 0.11	0.69 ± 0.12	0.293*
**8**	CgH intensity(0-10)	-	6.9 ± 1.4	7.2 ± 1.3	7.3 ± 1.3	0.461*
**9**	Headache	Unilateral	26 (81%)	25 (78%)	27 (84%)	-
		Bilateral	6 (19%)	7 (12%)	5 (16%)	-
**10**	Neck pain	Yes	28 (88%)	27 (84%)	28 (88%)	-
		No	4 (12%)	5 (16%)	4(12%)	-

* Non Significant, CSM – Cervical spine manipulation, TSM – Thoracic spine manipulation, CMT- Conventional physiotherapy, BMI – Body mass index, CgH – Cervicogenic Headache

Table 2: Pre and post mean and SD of outcome measures of CSM, TSM and CPT groups.

Sr.No	Variable		CSM	TSM	CPT	Group × Time p-value
**1**	**CgH Frequency (no of days per 4 weeks)**	Base line	16.8 ± 1.8	17.2 ± 1.9	17.4 ± 1.7	**0.012****
		4 weeks	11.2 ± 1.4	14.1 ± 1.6	15.8 ± 1.4	
		8 weeks	6.2 ± 0.9	9.5 ± 0.9	13.4 ± 1.1	$\eta p^2 = 0.092$
		6 months	2.9 ± 0.5	6.1 ± 0.7	10.8 ± 1.1	
**2**	**CgH Pain intensity (0 -10)**	Base line	7.2 ± 0.8	6.8 ± 0.7	7.1 ± 0.7	**0.022****
		4 weeks	4.1 ± 0.5	5.2 ± 0.5	6.2 ± 0.5	
		8 weeks	2.8 ± 0.4	4.1 ± 0.4	5.1 ± 0.4	$\eta p^2 = 0.078$
		6 months	0.8 ± 0.2	1.7 ± 0.3	3.7 ± 0.4	
**3**	**CgH Disability**	Base line	57.88 ± 6.5	57.21 ± 6.8	56.91 ± 5.9	**0.043****
		4 weeks	45.73 ± 5.5	49.38 ± 5.6	52.38 ± 5.3	
		8 weeks	36.41 ± 4.3	41.73 ± 5.1	49.67 ± 4.5	$\eta p^2 = 0.102$
		6 months	31.19 ± 3.8	39.54 ± 4.5	48.37 ± 4.1	
**4**	**Neck pain frequency (no of days per 4 weeks)**	Base line	23.9 ± 3.2	23.6 ± 3.4	22.9 ± 3.6	**0.007****
		4 weeks	15.8 ± 2.2	18.5 ± 2.4	20.4 ± 2.3	
		8 weeks	9.3 ± 1.6	12.3 ± 1.8	18.2 ± 1.5	$\eta p^2 = 0.086$
		6 months	3.4 ± 0.4	8.2 ± 0.9	16.1 ± 1.5	
**5**	**Neck pain intensity (0 -10)**	Base line	7.2 ± 0.6	7.1 ± 0.6	6.9 ± 0.5	**0.002****
		4 weeks	4.8 ± 0.5	6.2 ± 0.6	6.1 ± 0.5	
		8 weeks	2.6 ± 0.4	4.2 ± 0.5	5.1 ± 0.4	$\eta p^2 = 0.096$
		6 months	0.6 ± 0.2	1.9 ± 0.4	3.7 ± 0.4	
**6**	**Neck pain threshold**	Base line	262.1 ± 21.6	261.3 ± 22.3	262.7 ± 22.5	**0.015****
		4 weeks	268.5 ± 20.2	266.3 ± 21.8	263.5 ± 21.6	
		8 weeks	274.5 ± 18.3	272.4 ± 19.4	267.8 ± 19.6	$\eta p^2 = 0.104$
		6 months	288.2 ± 17.3	278.3 ± 18.2	269.5 ± 17.3	
**7**	**Flexion rotation test (Right side)**	Base line	25.18 ± 7.4	25.12 ± 7.3	26.01 ± 7.2	**0.008****
		4 weeks	31.76 ± 6.3	30.19 ± 6.3	27.12 ± 6.5	
		8 weeks	39.73 ± 5.6	35.16 ± 5.4	31.42 ± 6.1	$\eta p^2 = 0.091$
		6 months	45.21 ± 5.3	38.22 ± 5.1	32.31 ± 5.8	
**8**	**Flexion rotation test (Left side)**	Base line	24.93 ± 6.2	24.72 ± 6.1	24.88 ± 6.3	**0.006****
		4 weeks	33.12 ± 5.9	27.93 ± 5.9	26.63 ± 5.8	
		8 weeks	37.81 ± 5.2	32.53 ± 5.5	29.92 ± 5.1	$\eta p^2 = 0.074$
		6 months	44.23 ± 5.1	36.23 ± 5.1	31.92 ± 4.9	
**9**	**Neck Disability Index**	Base line	51.01 ± 11.1	51.25 ± 10.8	50.97 ± 11.2	**0.003****
		4 weeks	37.36 ± 9.2	43.34 ± 9.8	45.32 ± 10.2	
		8 weeks	24.83 ± 6.1	31.32 ± 6.2	38.53 ± 6.9	$\eta p^2 = 0.082$
		6 months	11.28 ± 3.4	23.56 ± 5.4	34.92 ± 6.3	
**10**	**Quality of life**	Base line	24.9 ± 4.5	25.1 ± 3.9	24.8 ± 4.2	**0.004****
		4 weeks	48.3 ± 4.8	36.3 ± 4.8	34.9 ± 4.3	
		8 weeks	64.5 ± 5.5	55.3 ± 5.1	40.1 ± 4.6	$\eta p^2 = 0.112$
		6 months	79.2 ± 6.2	62.4 ± 5.9	46.7 ± 4.9	

** Significant, CSM – Cervical spine manipulation, TSM – Thoracic spine manipulation, CPT- Conventional physiotherapy, CgH – Cervicogenic Headache

Table 3: Pre and post mean difference and confidence interval (upper limit and lower limit) scores of CSM, TSM and CPT groups.

Variable / Time		Baseline	4 weeks	8 weeks	6 months
		**Mean difference (95% CI)**			
**CgH Frequency**	**CSM × TSM**	0.4 (-0.67 to 1.47)	2.9 (2.02 to 3.77)	3.3 (2.72 to 3.87)	3.2 (2.71 to 3.68)
	**P – value**	0.649	0.001	0.001	0.001
	**CSM × CPT**	0.6 (-0.47 to 1.67)	4.6 (3.72 to 5.47)	7.2 (6.62 to 7.77)	7.9 (7.41 to 8.38)
	**P – value**	0.381	0.001	0.688	0.874
	**TSM × CPT**	0.2 (-0.87 to 1.27)	1.7 (0.82 to 2.57)	3.9 (3.32 to 4.47)	4.7 (4.21 to 5.18)
	**P – value**	0.897	0.001	0.001	0.004
**CgH Pain Intensity**	**CSM × TSM**	-0.4 (-0.83 to 0.03)	1.1 (0.80 to 1.39)	1.3 (1.06 to 1.53)	0.9 (0.71 to 1.08)
	**P – value**	0.080	0.001	0.001	0.001
	**CSM × CPT**	-0.1 (-0.53 to 0.33)	2.1 (1.80 to 2.39)	2.3 (2.06 to 2.53)	2.9 (2.71 to 3.08)
	**P – value**	0.849	0.001	0.003	0.874
	**TSM × CPT**	0.3 (-0.13 to 0.73)	1.0 (0.70 to 1.29)	1.0 (0.76 to 1.23)	2.0 (1.81 to 2.18)
	**P – value**	0.237	0.001	0.001	0.108
**CgH Disability**	**CSM × TSM**	-0.6 (-4.48 to 3.14)	3.6 (0.39 to 6.90)	5.3 (2.55 to 8.08)	8.3 (5.88 to 10.81)
	**P – value**	0.908	0.024	0.001	0.001
	**CSM × CPT**	-0.9 (-4.78 to 2.84)	6.6 (3.39 to 9.90)	13.2 (10.49 to 16.02)	17.1 (14.7 to 19.6)
	**P – value**	0.817	0.000	0.001	0.001
	**TSM × CPT**	-0.3 (-4.11 to 3.51)	3.00 (-0.25 to 6.25)	7.9 (5.17 to 10.70)	8.8 (6.3 to 11.2)
	**P – value**	0.980	0.774	0.001	0.001
**Neck pain frequency**	**CSM × TSM**	-0.3 (-2.32 to 1.7)	2.7 (1.32 to 4.07)	3.0 (2.02 to 3.97)	4.8 (4.18 to 5.41)
	**P – value**	0.933	0.001	0.001	0.001
	**CSM × CPT**	-1.0 (-3.02 to 1.02)	4.6 (3.22 to 5.97)	8.9 (7.92 to 8.97)	12.7 (12.08 to 13.31)
	**P – value**	0.470	0.001	0.002	0.874
	**TSM × CPT**	-0.7 (-2.72 to 1.32)	1.9 (0.52 to 3.27)	5.9 (4.92 to 6.87)	7.9 (7.28 to 8.51)
	**P – value**	0.690	0.003	0.001	0.746
**Neck pain intensity**	**CSM × TSM**	-0.1 (-0.43 to 0.23)	1.4 (1.08 to 1.71)	1.6 (1.34 to 1.85)	1.3 (1.09 to 1.50)
	**P – value**	0.762	0.001	0.001	0.001
	**CSM × CPT**	-0.3 (-0.63 to 0.03)	1.3 (0.98 to 1.61)	2.5 (2.24 to 2.75)	3.1 (2.89 to 3.30)
	**P – value**	0.093	0.001	0.001	0.873
	**TSM × CPT**	-0.2 (-0.53 to 0.13)	-0.1 (-0.41 to 0.21)	0.9 (0.64 to 1.15)	1.8 (1.59 to 2.00)
	**P – value**	0.341	0.736	0.001	0.001
**Neck pain threshold**	**CSM × TSM**	-0.8 (-13.98 to 12.38)	-2.2 (-14.83 to 10.43)	-2.1 (-13.47 to 9.27)	-9.9 (-20.38 to 0.58)
	**P – value**	0.988	0.909	0.899	0.068
	**CSM × CPT**	0.6 (-12.58 to 13.78)	-5 (-17.63 to 7.63)	-6.7 (-18.07 to 4.67)	-18.7 (-29.18 to -8.21)
	**P – value**	0.993	0.614	0.343	0.000
	**TSM × CPT**	1.4 (-11.78 to 14.58)	-2.8 (15.43 to 9.83)	-4.6 (-15.97 to 6.77)	-8.8 (-19.28 to 1.68)
	**P – value**	0.965	0.857	0.602	0.118
**Flexion rotation test (Right side)**	**CSM × TSM**	-0.06 (-4.40 to 4.28)	-1.5 (-5.36 to 2.22)	-4.5 (-7.9 to -1.1)	-6.9 (-10.21 to -3.76)
	**P – value**	0.980	0.587	0.005	0.001
	**CSM × CPT**	0.83 (-3.51 to 5.17)	-4.64 (-8.43 to -0.84)	-8.3 (-11.7 to -4.91)	-12.9 (-16.12 to -9.67)
	**P – value**	0.892	0.012	0.001	0.001
	**TSM × CPT**	0.89 (-3.45 to 5.23)	-3.07 (-6.86 to 0.72)	-3.74 (-7.13 to -0.34)	-5.91 (-9.13 to -2.68)
	**P – value**	0.877	0.136	0.027	0.001
**Flexion rotation test (Left side)**	**CSM × TSM**	-0.21 (-3.90 to 3.48)	-5.19 (-8.68 to -1.69)	-5.2 (-8.41 to -2.14),	-8.0 (-10.99 to -5.00)
	**P – value**	0.989	0.001	0.001	0.001
	**CSM × CPT**	-0.05 (-3.74 to 3.64)	-6.4 (-9.98 to -2.99)	-7.8 (-11.02 to -4.75)	-12.31 (-15.30 to -9.31)
	**P – value**	1.032	0.001	0.001	0.001
	**TSM × CPT**	0.16 (-3.53 to 3.85)	-1.3 (-4.79 to 2.19)	-2.61 (-5.74 to 0.52)	-4.31 (-7.30 to -1.31)
	**P – value**	0.994	0.650	0.122	0.002
**Neck Disability Index**	**CSM × TSM**	0.24 (-6.3 to 6.81)	5.98 (0.17 to 11.78)	6.49 (2.67 to 10.30)	12.28 (9.19 to 15.36)
	**P – value**	0.993	0.041	0.001	0.001
	**CSM × CPT**	-0.04 (-6.61 to 6.53)	7.96 (2.15 to 13.76)	13.7 (9.88 to 17.51)	23.64 (20.55 to 26.72)
	**P – value**	0.803	0.004	0.001	0.001
	**TSM × CPT**	-0.28 (-6.85 to 6.29)	1.98 (-3.82 to 7.78)	7.21 (3.39 to 11.02)	11.36 (8.27 to 14.44)
	**P – value**	0.994	0.696	0.001	0.001
**Quality of life**	**CSM × TSM**	0.20 (-2.30 to 2.70)	-12.0 (-14.76 to -9.23)	-9.2 (-12.2 to -6.1)	-16.8 (-20.19 to -13.40)
	**P – value**	0.980	0.001	0.001	0.001
	**CSM × CPT**	-0.10 (-2.60 to 2.40)	-13.4 (-16.16 to -10.63)	-24.4 (-27.4 to -21.37)	-32.5 (-35.89 to -29.10)
	**P – value**	0.996	0.001	0.001	0.002
	**TSM × CPT**	-0.30 (-2.80 to 2.20)	-1.4 (-4.16 to 1.36)	-15.20 (-18.22 to -12.17)	-15.7 (-19.09 to -12.30)
	**P – value**	0.956	0.452	0.001	0.001

CSM – Cervical spine manipulation, TSM – Thoracic spine manipulation, CPT- Conventional physiotherapy, CgH – Cervicogenic Headache

Supporting files:

1. CONSORT 2010 Check list.
2. Study trial protocol.
3. Ethical approval certificate
4. Dataset

Dear Editor and reviewers,

Thank you for your recent correspondence regarding our paper on ‘Comparative effectiveness of cervical vs thoracic spinal-thrust manipulation for care of cervicogenic headache: a randomized controlled trial.” We would like to thank you for the opportunity to submit a revised manuscript. We appreciate all the comments, suggestions, and positive criticism raised by the Editor and the reviewers which were of great value, and certainly helped us to improve the quality of our manuscript. We made all possible efforts to properly address and/or reply to the comments raised. Additionally, a complete review of the article was done for grammar and readability. A significant number of modifications were made to the article after several reviews. No large disagreements were made with the reviewer’s comments. The authors of this paper greatly thank each of the reviewers for spending time and provided such a constructive comments and fantastic input. The authors greatly appreciate the work of all the reviewers as well as the editors on this manuscript. Many points were a great addition to the quality of this article. Here is a corrected manuscript with a point-by-point response to comments from the reviewers. If any additional corrections can be corrected in the further rounds of revisions.

Regards

Dr. Gopal

Reviewer 1

This is an interesting study and the protocol looks sound, however, the manipulation techniques, as described, do not look sound at all. Not contemporary manual therapy practice. See my comments in the pdf file.

With some major revisions, this could be a great paper for publication.

Author response: Thank you for this positive and constructive comment and as per your suggestion the required changes have been done in the manuscript and the changes were highlighted.

Reviewer 2

This was a novel study that gives first evidence for a difference between cervical and thoracic manipulation for cervicogenic headache, as well as an advantage for manipulation over massage used in conventional physical therapy. On the whole, the study is well designed with appropriate. primary and secondary outcomes for CGH. There was excellent adherence to care and compliance with data collection.

There are some limitations that need to be considered. The sample size was small albeit the study was powered. The standard for headache outcomes is a headache diary that uses technology to ensure that frequency and pain intensity are reported immediately on the day stipulated, not added in later. Also, the conventional physical therapy intervention appears to have visits of longer duration than the manipulation visits which could have introduced attention bias and reduced treatment effect estimates. The manuscript needs work. More details and language editing are required.

Author response: Thank you so much for accepting our article to review and suggested positive and constructive comments, which really enhance the quality of our article.

Reviewer comment: 1 Title: Try "Comparative effectiveness of cervical vs thoracic spinal-thrust manipulation for care of cervicogenic headache: a randomized controlled trial"

Author response: Thank you for this comment and as per your suggestion the title has been changed.

Reviewer comment: 2 Introduction: 1st paragraph: Give a citation for CGH having socioeconomic impact and being a significant public health burden.

Author response: Thank you for this comment and as per your suggestion the reference has been added.

Reviewer comment: 3 Reference 13: This was a pilot study. A full-scale RCT on dose-response was published in 2018 in The Spine Journal. This would be a better reference.

Author response: Thank you for this comment and as per your suggestion the reference number 13 has been updated.

Reviewer comment: 4 Material and Methods: Design: Blinding: This study was not "single blind." This designation would require that the participant or provider was blinded. The assessor was blinded for data collection, but for the primary outcome the assessor was the participant, so the study cannot properly be called assessor-blind either.

Author response: Thank you for this comment and as per your suggestion the required changes have been done in the blinding section.

Reviewer comment: 5 Participants: Include a reference for CONSORT.

Author response: Thank you for this comment and as per your suggestion the reference for the CONSORT guidelines was added.

Reviewer comment: 6 Interventions: State that all three groups received ten minutes of hydro collator heat prior to intervention (manipulation or massage).

Author response: Thank you for this comment and as per your suggestion the suggested statement was added in the intervention section.

Reviewer comment: 7 Spinal Manipulation Therapy: Include the reference number for "Peterson and Bergman." Was a "cracking" sound required for the manipulation to be considered successful?

Author response: Thank you for this comment and the reference for "Peterson and Bergman" was added and the information regarding the cracking sound is also included in the intervention section.

Reviewer comment: 8 Cervical Manipulation: Were multiple segment-specific manipulations permitted on each side based on segmental dysfunction or was a single general, nonspecific manipulation performed on each side?

Author response: Thank you for this comment and the information regarding the cervical spine manipulation was added.

Reviewer comment: 9 CGH Disability: Briefly explain how the HIT questionnaire is scored: scale for each of the six items and direction of scoring, totaling the score, and total scale range.

Author response: Thank you for this comment and the details regarding the HIT questionnaire was added in the CGH disability section.

Reviewer comment: 10 Sample size: Why was a four day's difference between groups in CGH frequency chosen for the sample size calculation? Is this a clinically important difference between groups?

Author response: Thank you for this comment. Yes, this is the expected minimum clinically important difference (MCID) score between the two groups.

Reviewer comment: 11 Analysis: Baseline comparisons: Baseline statistical comparisons should not be performed. This is because in a randomized trial, baseline group differences are attributable to chance by definition, due to random allocation of participants. Remove baseline comparisons from text and tables.

Author response: Thank you for this comment and as per your suggestion the baseline comparisons have been removed from the manuscript and tables.

Reviewer comment: 12 Analysis: LMM: Further explain the model used. Was this a random-intercept model? If appropriate, identify which covariance structure was used.

Author response: Thank you for this comment and as per your suggestion the required information have been added in the statistical analysis part.

Reviewer comment: 13 Analysis: Post hoc analysis: Include here that post hoc Bonferroni analysis was use following the omnibus 3x4 LMM model to compare the three study groups pair-wise.

Author response: Thank you for this comment and as per your suggestion the required changes have been added in the statistical analysis part.

Reviewer comment: 14 Analysis: Intention-to-Treat: Include this under Analysis, not Results Participants. Explain how you define intention-to treat because there are different definitions. Were participants with missing follow-up data included in the analysis?

Author response: Thank you for this comment and as per your suggestion the intention to principle statement was moved to statistical analysis part and detail information was included.

Reviewer comment: 15 Results: Participants: Baseline characteristics: Make a general statement that the three groups were uniform in baseline characteristics and baseline outcome variables. CGH was defined as unilateral, yet there were participants with bilateral headache. Explain why these were included?

Author response: Thank you for this comment and as per your suggestion the required changes have been done in the manuscript. We are sorry for this typographical mistake and the changes have been done in the clinical definition.

Reviewer comment: 16 Partial eta-squared: Remove this statistic from the text and tables. This effect size statistic is uncommon in biomedical research (usually found in the social sciences literature). It is also not defined or interpreted in this manuscript, so has no use.

Author response: Thank you for this comment and as per your suggestion the Partial eta-squared was removed from the results part and also from the tables.

Reviewer comment: 17 Table 2: Show score range for NDI (0-100 with 100 worst) and EQ-5D (0-100 with 100 best) in the second column.

Author response: Thank you for this comment and as per your suggestion the required changes have been done in the table 2.

Reviewer comment: 18 Table 3: CGH frequency: There are errors in the p-values for CSM x CPT at 8 weeks and 6 months.

Author response: Thank you for this comment and as per your suggestion the changes have been done in the table 3.

Reviewer comment: 19 Discussion: Organization: I suggest you start by stating this is the first powered study to compare cervical and thoracic manipulation for CGH and that the study shows that cervical manipulation was superior to thoracic manipulation and conventional PT (massage). Then compare studies and speculate about mechanisms.

Author response: Thank you for this comment and as per your suggestion the required changes have been done in the first paragraph of the discussion.

Reviewer comment: 20 2nd Paragraph: This paragraph is confusing. Statistically significant changes: This means a within-groups analysis of change over time. No within-groups analysis is identified under Methods or reported under Results. I think you are talking about between groups differences in improvement favoring CSM over TSM and CPT.

You report mean differences between groups from Table 3, not MCID. You can say that the differences between groups are clinically important, if you can give references that supports such claims. Also, start with the primary outcome, CGH frequency. Then discuss pain. This study did not investigate thrust duration. You do not know if the thrust is less than 2ms in this study.

Perhaps change this paragraph into two separate paragraphs: one discussing clinical outcomes and the second discussing mechanism and theory.

Author response: Thank you for this comment and as per your suggestion the required changes have been done in the 2nd paragraph of the manuscript.

Reviewer comment: 21 Limitations: Differences in the length of study visits between groups adds attention bias and could reduce differences between manipulation groups and the massage group.

Author response: Thank you for this comment and as per your suggestion the above mentioned limitation was added in the limitation part.

Reviewer comment: 22 Conclusion: The conclusion in the abstract is more accurate and better written. You can use it again here and add a sentence or so to recommend a future research trajectory.

Author response: Thank you for this comment and as per your suggestion the conclusion part was modified.

Reviewer comment: 23 Strong evidence: This is a technical term used in systematic reviews. Strong evidence usually means multiple, high-quality trials with similar results.

Author response: Thank you for this comment and as per your suggestion the term “strong” was replaced in the conclusion part.
