# Supplementary material for: Comparative effectiveness of cervical vs thoracic spinal-thrust manipulation for care of cervicogenic headache: A randomized controlled trial
Source: PLoS One. 2024 Mar 29;19(3):e0300737. doi: 10.1371/journal.pone.0300737 (PMC10980233; doi:10.1371/journal.pone.0300737)
Supplement: Attachment — Submitted filename: Response to reviewer - 1.docx [file pone.0300737.s005.docx]

Dear Editor and reviewers,

Thank you for your recent correspondence regarding our paper on ‘Comparative effectiveness of cervical vs thoracic spinal-thrust manipulation for care of cervicogenic headache: a randomized controlled trial.” We would like to thank you for the opportunity to submit a revised manuscript. We appreciate all the comments, suggestions, and positive criticism raised by the Editor and the reviewers which were of great value, and certainly helped us to improve the quality of our manuscript. We made all possible efforts to properly address and/or reply to the comments raised. Additionally, a complete review of the article was done for grammar and readability. A significant number of modifications were made to the article after several reviews. No large disagreements were made with the reviewer’s comments. The authors of this paper greatly thank each of the reviewers for spending time and provided such a constructive comments and fantastic input. The authors greatly appreciate the work of all the reviewers as well as the editors on this manuscript. Many points were a great addition to the quality of this article. Here is a corrected manuscript with a point-by-point response to comments from the reviewers. If any additional corrections can be corrected in the further rounds of revisions.

Regards

Dr. Gopal

**Reviewer 1**

This is an interesting study and the protocol looks sound, however, the manipulation techniques, as described, do not look sound at all. Not contemporary manual therapy practice.
See my comments in the pdf file.

With some major revisions, this could be a great paper for publication.

**Author response:** Thank you for this positive and constructive comment and as per your suggestion the required changes have been done in the manuscript and the changes were highlighted.

**Reviewer 2**

This was a novel study that gives first evidence for a difference between cervical and thoracic manipulation for cervicogenic headache, as well as an advantage for manipulation over massage used in conventional physical therapy. On the whole, the study is well designed with appropriate. primary and secondary outcomes for CGH. There was excellent adherence to care and compliance with data collection.

There are some limitations that need to be considered. The sample size was small albeit the study was powered. The standard for headache outcomes is a headache diary that uses technology to ensure that frequency and pain intensity are reported immediately on the day stipulated, not added in later. Also, the conventional physical therapy intervention appears to have visits of longer duration than the manipulation visits which could have introduced attention bias and reduced treatment effect estimates. The manuscript needs work. More details and language editing are required.
**Author response:** Thank you so much for accepting our article to review and suggested positive and constructive comments, which really enhance the quality of our article.

**Reviewer comment: 1** Title: Try “Comparative effectiveness of cervical vs thoracic spinal-thrust manipulation for care of cervicogenic headache: a randomized controlled trial”

**Author response:** Thank you for this comment and as per your suggestion the title has been changed.

**Reviewer comment: 2** Introduction: 1st paragraph: Give a citation for CGH having socioeconomic impact and being a significant public health burden.

**Author response:** Thank you for this comment and as per your suggestion the reference has been added.

**Reviewer comment: 3** Reference 13: This was a pilot study. A full-scale RCT on dose-response was published in 2018 in The Spine Journal. This would be a better reference.

**Author response:** Thank you for this comment and as per your suggestion the reference number 13 has been updated.

**Reviewer comment: 4** Material and Methods: Design: Blinding: This study was not “single blind.” This designation would require that the participant or provider was blinded. The assessor was blinded for data collection, but for the primary outcome the assessor was the participant, so the study cannot properly be called assessor-blind either.

**Author response:** Thank you for this comment and as per your suggestion the required changes have been done in the blinding section.

**Reviewer comment: 5** Participants: Include a reference for CONSORT.

**Author response:** Thank you for this comment and as per your suggestion the reference for the CONSORT guidelines was added.

**Reviewer comment: 6** Interventions: State that all three groups received ten minutes of hydro collator heat prior to intervention (manipulation or massage).

**Author response:** Thank you for this comment and as per your suggestion the suggested statement was added in the intervention section.

**Reviewer comment: 7** Spinal Manipulation Therapy: Include the reference number for “Peterson and Bergman.” Was a “cracking” sound required for the manipulation to be considered successful?

**Author response:** Thank you for this comment and the reference for “Peterson and Bergman” was added and the information regarding the cracking sound is also included in the intervention section.

**Reviewer comment: 8** Cervical Manipulation: Were multiple segment-specific manipulations permitted on each side based on segmental dysfunction or was a single general, nonspecific manipulation performed on each side?

**Author response:** Thank you for this comment and the information regarding the cervical spine manipulation was added.

**Reviewer comment: 9** CGH Disability: Briefly explain how the HIT questionnaire is scored: scale for each of the six items and direction of scoring, totaling the score, and total scale range.

**Author response:** Thank you for this comment and the details regarding the HIT questionnaire was added in the CGH disability section.

**Reviewer comment: 10** Sample size: Why was a four day’s difference between groups in CGH frequency chosen for the sample size calculation? Is this a clinically important difference between groups?

**Author response:** Thank you for this comment. Yes, this is the expected minimum clinically important difference (MCID) score between the two groups.

**Reviewer comment: 11** Analysis: Baseline comparisons: Baseline statistical comparisons should not be performed. This is because in a randomized trial, baseline group differences are attributable to chance by definition, due to random allocation of participants. Remove baseline comparisons from text and tables.

**Author response:** Thank you for this comment and as per your suggestion the baseline comparisons have been removed from the manuscript and tables.

**Reviewer comment: 12** Analysis: LMM: Further explain the model used. Was this a random-intercept model? If appropriate, identify which covariance structure was used.

**Author response:** Thank you for this comment and as per your suggestion the required information have been added in the statistical analysis part.

**Reviewer comment: 13** Analysis: Post hoc analysis: Include here that post hoc Bonferroni analysis was use following the omnibus 3x4 LMM model to compare the three study groups pair-wise.

**Author response:** Thank you for this comment and as per your suggestion the required changes have been added in the statistical analysis part.

**Reviewer comment: 14** Analysis: Intention-to-Treat: Include this under Analysis, not Results Participants. Explain how you define intention-to treat because there are different definitions. Were participants with missing follow-up data included in the analysis?

**Author response:** Thank you for this comment and as per your suggestion the intention to principle statement was moved to statistical analysis part and detail information was included.

**Reviewer comment: 15** Results: Participants: Baseline characteristics: Make a general statement that the three groups were uniform in baseline characteristics and baseline outcome variables. CGH was defined as unilateral, yet there were participants with bilateral headache. Explain why these were included?

**Author response:** Thank you for this comment and as per your suggestion the required changes have been done in the manuscript. We are sorry for this typographical mistake and the changes have been done in the clinical definition.

**Reviewer comment: 16** Partial eta-squared: Remove this statistic from the text and tables. This effect size statistic is uncommon in biomedical research (usually found in the social sciences literature). It is also not defined or interpreted in this manuscript, so has no use.

**Author response:** Thank you for this comment and as per your suggestion the Partial eta-squared was removed from the results part and also from the tables.

**Reviewer comment: 17** Table 2: Show score range for NDI (0-100 with 100 worst) and EQ-5D (0-100 with 100 best) in the second column.

**Author response:** Thank you for this comment and as per your suggestion the required changes have been done in the table 2.

**Reviewer comment: 18** Table 3: CGH frequency: There are errors in the p-values for CSM x CPT at 8 weeks and 6 months.

**Author response:** Thank you for this comment and as per your suggestion the changes have been done in the table 3.

**Reviewer comment: 19** Discussion: Organization: I suggest you start by stating this is the first powered study to compare cervical and thoracic manipulation for CGH and that the study shows that cervical manipulation was superior to thoracic manipulation and conventional PT (massage). Then compare studies and speculate about mechanisms.

**Author response:** Thank you for this comment and as per your suggestion the required changes have been done in the first paragraph of the discussion.

**Reviewer comment: 20** 2nd Paragraph: This paragraph is confusing. Statistically significant changes: This means a within-groups analysis of change over time. No within-groups analysis is identified under Methods or reported under Results. I think you are talking about between groups differences in improvement favoring CSM over TSM and CPT.

You report mean differences between groups from Table 3, not MCID. You can say that the differences between groups are clinically important, if you can give references that supports such claims. Also, start with the primary outcome, CGH frequency. Then discuss pain.
This study did not investigate thrust duration. You do not know if the thrust is less than 2ms in this study.

Perhaps change this paragraph into two separate paragraphs: one discussing clinical outcomes and the second discussing mechanism and theory.

**Author response:** Thank you for this comment and as per your suggestion the required changes have been done in the 2^nd^ paragraph of the manuscript.

**Reviewer comment: 21** Limitations: Differences in the length of study visits between groups adds attention bias and could reduce differences between manipulation groups and the massage group.

**Author response:** Thank you for this comment and as per your suggestion the above mentioned limitation was added in the limitation part.

**Reviewer comment: 22** Conclusion: The conclusion in the abstract is more accurate and better written. You can use it again here and add a sentence or so to recommend a future research trajectory.

**Author response:** Thank you for this comment and as per your suggestion the conclusion part was modified.

**Reviewer comment: 23** Strong evidence: This is a technical term used in systematic reviews. Strong evidence usually means multiple, high-quality trials with similar results.

**Author response:** Thank you for this comment and as per your suggestion the term “strong” was replaced in the conclusion part.
