# Supplementary material for: Comparative effectiveness of cervical vs thoracic spinal-thrust manipulation for care of cervicogenic headache: A randomized controlled trial
Source: PLoS One. 2024 Mar 29;19(3):e0300737. doi: 10.1371/journal.pone.0300737 (PMC10980233; doi:10.1371/journal.pone.0300737)
Supplement: Attachment — Submitted filename: Response to reviewer - 2.docx [file pone.0300737.s007.docx]

**Editor comments:**

Dear authors, thank you for your answers. You have compared the clinical effects of cervical over thoracic spine manipulation and conventional physiotherapy in patients with CgH. Please, when you review the article, in addition to highlighting the helped text, you must inform the page (and lines) of each of the adjustments. This will help you to get an opinion quickly.

**Author response:** Thank you for the comments and we have carefully considered all your comments and corrected the revised manuscript.

**Editor comment 1:** —Regarding minimum clinically important difference (MCID), please report the MCID for each of your outcomes and report it in the results. The study limitations should highlight outcomes that do not have an MCID established in the literature.

**Author response:** Thank you for the comments and as per your suggestion the MCID values of all the outcome measures were added.

**Editor comment 2:** —Please report the effect size value for all comparisons performed—the p-value does not indicate clinical significance ([https://pubmed.ncbi.nlm.nih.gov/36325112/](https://www.rediffmail.com/cgi-bin/red.cgi?red=https%3A%2F%2Fpubmed.ncbi.nlm.nih.gov%2F36325112%2F&isImage=0&BlockImage=0&rediffng=0&rdf=VnMHbVcpUScBNQA7AQ1TYFpmVyMAOlI7&rogue=a19a39ef5c5217b0fc717222f034f90cef09c347)). You should calculate the effect size using Cohen's d-value for quantitative variables; and using Cohen's w-value for % (use this online calculator: [https://www.psychometrica.de/effect_size.html](https://www.rediffmail.com/cgi-bin/red.cgi?red=https%3A%2F%2Fwww.psychometrica.de%2Feffect_size.html&isImage=0&BlockImage=0&rediffng=0&rdf=VnMHbVcpUScBNQA7AQ1TYFpmVyMAOlI7&rogue=81d8ad0e18dfb884a8c9d411e26aefaea5923e25)). By the way, this article may help you classify the d and w values of the effect size: [https://pubmed.ncbi.nlm.nih.gov/37971135/.](https://www.rediffmail.com/cgi-bin/red.cgi?red=https%3A%2F%2Fpubmed.ncbi.nlm.nih.gov%2F37971135%2F.&isImage=0&BlockImage=0&rediffng=0&rdf=VnMHbVcpUScBNQA7AQ1TYFpmVyMAOlI7&rogue=ba0d9355764159d4800a087bdfec9ec9e34a57ca)

**Author response:** Thank you for the comments and as per your suggestion the effect size Cohen’s d values of all the outcome measures were added.

**Editor comment 3:** —Table 1: The reported p-value refers to which comparison? This table needs to present 3 p-values; where are they? First p-value: CSM x TSM; Second p-value: CSM x CPT; third p-value: TSM x CPT. Remember to also report the effect size for each of these comparisons. Furthermore, correct the terms weight (the correct term is body mass [kg]) and height (the correct term is stature [cm or m]).

**Author response:** Thank you for the comments and as per your suggestion this issue was discussed with our research statistician. He suggested that this is the demographic and clinical characteristics of the study participants’ analysis between the three groups. As per his suggestion, the effect size is not required for the above in this table. Also, the terms weight and height were modified in table 1.

**Editor comment 4:** —Table 2: The p-value reported refers to which comparison (CSM x TSM, CSM x CPT, TSM x CPT)? This table also needs to present 3 p-values; where are they? In the legend you must detail the information in the table, inform the tests that generated the p-value, and the alpha established for statistical significance. Tables must be intuitive (remember to also inform the effect size for each of these comparisons).

**Author response:** Thank you for the comments and as per your suggestion the p values for (CSM x TSM, CSM x CPT, TSM x CPT) are mentioned in Table 3.

**Editor comment 5:** —Table 3: In addition to the p-value, report the effect size for each of these comparisons.

**Author response:** Thank you for the comments and as per your suggestion the effect size for each of these comparisons was mentioned in Table 3.

**Reviewer #1:**

**Author response:** Thank you for the comments and we are extremely grateful to you for providing suggestions to improve our manuscript quality. Also, as per your direction we have changed all the required modifications in the manuscript.

**Reviewer #2:**

This manuscript has been greatly improved by the authors. In the process, however, several changes that the authors said they made are missing from the revised manuscript.

I also made some suggestions for language improvements to the revisions in the Discussion and Conclusions.

**Author response:** Thank you for the comments and we are extremely sorry and apologize for the missing items in the revised manuscript. This time we have carefully considered all your comments and corrected the revised manuscript.

**Reviewer comment 1:** Material and Methods

1. Sample size: State that 4 CgH days is considered the MCID between groups and include a citation.

**Author response:** Thank you for the comments and as per your suggestion the required changes have been made in the statistical analysis part. The MCID and SD are obtained from the previous pilot study, which was mentioned in the section and the study was not published.

**Reviewer comment 2:** 2. Analysis: Baseline comparisons (Comment 11 from the first review):
The authors state that they removed the statistical comparisons of baseline variables between groups. This is not the case. Statements of no significance and/or p-values still appear in the following and these must be removed:

Abstract: Results: First sentence. - Removed

Results: Participants: Two sentences on demographics and clinical variables. - Removed

(The new last sentence is supposed to replace the statistical comparisons with p-values). - Modified

Table 1: last column - Removed

Table 3: Baseline column - Removed

**Author response:** Thank you for the comments and as per your suggestions the required changes have been done in the manuscript.

**Reviewer comment 3:** 3. Analysis: Intention-to-Treat:

This has parts:

You added that all patients were included in the analysis even if data were missing.
Also add that participants were analyzed in the group to which they were randomized.

**Author response:** Thank you for the comments and as per your suggestions the required changes have been made in the manuscript.

**Reviewer comment 4:** Results 4. Partial eta-squared: (comment 16 from first review):

As per the response to reviewer comments, partial eta-squared has been removed from the text.

However, it remains to be removed from Table 2.

**Author response:** Thank you for the comments and as per your suggestions the required changes have been made in the table 2.

**Reviewer comment 5:** 5. Table 2: (Comment 17 from first review):

This has not been done as stated by the authors in the response to reviewer comments.
“Show score range for NDI (0-100 with 100 worst) and EQ-5D (0-100 with 100 best) in the second column.”

**Author response:** Thank you for the comments and as per your suggestions the required changes have been made in the table 2.

**Reviewer comment 6:** Discussion 6. 1st Paragraph:

Start the paragraph like this: “This was the first powered randomized trial to compare the effects of cervical and thoracic manipulation for patients with CgH. Cervical manipulation was found to be superior to thoracic manipulation and conventional PT (massage) for improving days with CgH, as well as headache and neck pain and disability, to 6 months.”

Make sure to check the grammar.

Briefly elaborate the contradiction with Borusiak et al. What did these authors find?

**Author response:** Thank you for the comments and as per your suggestions the required changes have been made in the 1^st^ paragraph of discussion.

**Reviewer comment 7:** 7. 2nd Paragraph:

Start the paragraph like this: “The mechanism of action has yet to be determined. Manipulation of the cervical spine may promote afferent nerve fiber activity through stimulation of the cervical

joint receptors. It may improve…”

**Author response:** Thank you for the comments and as per your suggestions the required changes have been made in the 2^nd^ paragraph of discussion.

**Reviewer comment 8:** 8. 3rd Paragraph: Start the paragraph like this: “Thoracic manipulation was also found to be more effective than conventional PT in improving both the primary and secondary outcomes.”

**Author response:** Thank you for the comments and as per your suggestions the required changes have been made in the 3^rd^ paragraph of discussion.

**Reviewer comment 9:** Conclusion 9. Try this wording: “The current randomized controlled trial found that cervical spine manipulation was more effective in improving pain parameters (intensity, frequency and threshold), functional disability and quality of life in patients with cervicogenic headache than thoracic spine manipulation and conventional physiotherapy. Future studies are recommended to identify the biomechanical and biochemical mechanisms underlying the clinical and functional changes engendered by manipulation in the treatment of CgH.”

**Author response:** Thank you for the comments and as per your suggestions the required changes have been made in the conclusion.
