# Supplementary material for: Comparative effectiveness of cervical vs thoracic spinal-thrust manipulation for care of cervicogenic headache: A randomized controlled trial
Source: PLoS One. 2024 Mar 29;19(3):e0300737. doi: 10.1371/journal.pone.0300737 (PMC10980233; doi:10.1371/journal.pone.0300737)
Supplement: Attachment — Submitted filename: Response to reviewer - 3.docx [file pone.0300737.s008.docx]

**Editor comments:**

**Editor comment:** Dear authors, thank you for your replies. In the last review, I kindly asked you to indicate the page and lines of each change, but you submitted the article without this information. This complicates and delays the review process. Next time, I will consider this a reason to reject your article. In this review, in addition to highlighting the helped text, you need to report the page (and lines) of each of the changes.

Please follow the last adjustment of the article indicated by the peer-reviewer and me and submit it.

**Author response:** Dear Editor, we are extremely sorry and apologize for the above issue happened from our side and we know how much difficult it is to review the comments suggested by you and other reviewers. Therefore, in this review we have included the page number and line number for all the corrections we have made and thank you for giving this opportunity to resubmit our article.

**Editor comment 1:** —Regarding minimum clinically important difference (MCID), please report the MCID for each of your outcomes and report it in the results. Outcomes that do not have an MCID established in the literature should be highlighted in the study limitations.

**Author response:** Thank you for the suggestion and as per your recommendation the MCID values of all the outcomes were added in the results section and highlighted with red color.

**Page Number:** 13	**Line number:** 27-31

**Editor comment 2:** —Delete the "Sr. No." column in Tables 1 and 2.

**Author response:** Thank you for the suggestion and as per your recommendation the "Sr. No." column in Tables 1 and 2 was removed.

**Page Number:** 20, 21	**Line number:** 1

**Editor comment 3:** —Table 1: Remove the p-value column.

**Author response:** Thank you for noticing the above mistake, and as per your suggestion the last column in the table 1 has been removed.

**Page Number:** 20	**Line number:** 1

**Editor comment 4:** —Table 2: This table remains problematic and unintuitive. I'll give an example: the p-value of 0.012** in the first row indicates that the groups have a significant difference, but the table describes three groups, and we know that the TSM and CPT groups have similar scores at baseline (TSM: 17.2 ± 1.9 vs. CPT 17.4 ± 1.7 [p-value?]), while CSM is different from them (16.8 ± 1.8). there are 3 independent comparisons (e.g., 1: TSM vs. CPT [p-value]; 2: TSM vs. CSM [p-value]; 3: CPT vs. CSM [p-value]), so it is necessary to report 3 p-values (one for each comparison). What is important for the RCT is not the overall variance, but the difference between the groups at different times (as in Table 3).

How do you fix this error? First, delete the p-value column from Table 2 (leaving only the descriptive data); second, add a column to Table 3 and do the same analysis for the baseline time. I have made an example for you (available for download), go to this link and use this model and its headers+legends (Tables 1, 2, and 3):

< [https://docs.google.com/document/d/1NdsUtg3x0BSAmpJ3kKI9rDYy_8VL6slb/edit?usp=sharing&ouid=104821689851272179944&rtpof=true&sd=true](https://links.rediff.com/cgi-bin/red.cgi?red=https%3A%2F%2Fdocs%2Egoogle%2Ecom%2Fdocument%2Fd%2F1NdsUtg3x0BSAmpJ3kKI9rDYy%5F8VL6slb%2Fedit%3Fusp%3Dsharing%26ouid%3D104821689851272179944%26rtpof%3Dtrue%26sd%3Dtrue&isImage=0&BlockImage=0&rediffng=0&rogue=a9421ab29772c0d03cb0e69661eb5c8087546b79&rdf=XntUPgZ4AnRcaAA7V1sCMVVpXytaYANq) >

**Author response:** Thank you for this important suggestion and as per your recommendation the p-values in the table 2 has been removed.

The p values for each comparison (CSM vs TSM, CSM vs CPT and TSM vs CPT) is highlighted in the Table -3.

As per the recommendation of second reviewer the column for the baseline comparison was removed from the Table 3 during the second review.

The header and footer of the Tables 1, 2, and 3 were modified as per your recommendation.

**Page Number:** 20-22	**Line number:** 1

**Editor comment 5:** —Finally, if you need to adjust the results session wording because of the table changes, do so.

**Author response:** Thank you for the suggestion and required changes have been done in the results section.

**Page Number:** 12	**Line number:** 27

**Reviewer #2:**

**Reviewer comment 1:** Not all the p-values for baseline comparisons have been removed. Please remove them: Results: Participants: The p-value language has not been removed from the end of the paragraph" p > .05". Remove it.

**Author response:** Thank you for noticing the above mistake, and as per your suggestion the sentence has been removed.

**Page Number:** 12	**Line number:** 25

**Reviewer comment 2:** Table 1: last column. The p-values column has not been removed. Remove it.

**Author response:** Thank you for noticing the above mistake, and as per your suggestion the last column in the table 1 has been removed.

**Page Number:** 20	**Line number:** 1

**Reviewer comment 3:** Table 3: At the bottom of the table, identify d: “d – Cohen’s d (effect size)”

**Author response:**

**Page Number:** 22	**Line number:** 4
